# A structural analysis of U.S. homelessness through Markov random fields: Identifying predictors and potential causal links through permutation tests

**DOI:** 10.1371/journal.pone.0343842

**Published:** 2026-11-04

**Authors:** Curtis Smith, Moinak Bhaduri

**Affiliations:** 1 Sociology Department, Bentley University, Boston, Massachusetts, United States of America; 2 Mathematical Sciences Department, Bentley University, Boston, Massachusetts, United States of America; Tsinghua University, CHINA

## Abstract

Many researchers who study homelessness argue that traditional frameworks are often too narrow, failing to account for groups outside the reach of social services. Despite historic calls to broaden the scope of sampling to encompass structural forces, only recent advancements have enabled the development of a robust framework for accurate measurement and analysis. This study utilizes Markov random fields (MRFs) to explore the influences of key structural indicators on homelessness from 2007 to 2024. By employing partial correlations, we investigate the interplay among geographic locations at the state-level among the following variables: the job market, median rent, eviction rates, occupancy rates, median income, the construction of new homes, the cost of one-bedroom units, funding from Housing and Urban Development (HUD), and policies under democrat or republican elections (by state). Our findings reveal that rent, income, and the availability of single-bedroom units are the foremost predictors of homelessness rates, underscoring the significance of housing affordability and availability as central determinants. The analysis also distinguishes variation in conditional dependence patterns between states governed by different political parties, even as they change, spotlighting substantial differences in the partial association rates across partisan lines. The results emphasize the need for policy reforms targeted at increasing housing affordability over more long-term strategies, such as the construction of new homes, and suggest that political contexts distinctly shape the effectiveness of structural interventions. These insights offer a valuable framework for policymakers aiming to address the root causes of homelessness through targeted structural reforms. An exploratory lag-1 directed temporal network reveals that the HUD↔homelessness feedback cycle is empirically present especially in Republican-led states; we report this as suggestive (cluster-permutation p = 0.12) and as a hypothesis for future panel-VAR work. We frame the conditional-dependence structure as descriptive rather than causal, state the assumptions a causal reading would require, and provide four supplementary specifications — cluster bootstrap, two-way state-and-year fixed-effects residualisation, nonparanormal transformation, and Fast Causal Inference — that probe each assumption.

## Introduction

Despite being studied for over a hundred years in the United States [1,2], homelessness remains an unresolved and contentious issue among public discussion, policy makers, and academic circles. For instance, from 2020 to 2025, homelessness increased over 30% after the economic decline initiated by COVID-19 [3]. Given the pressing nature of the issue, several studies have attempted to examine whether homelessness rates are impacted by structural indicators, such as the housing market and job market [4,5]. Typically, such studies deploy some variation of a correlational analysis from which insights on causal links are difficult to extract. In addition, to the best of our knowledge, a thorough study involving a large collection of potential structural predictors of homelessness over the span of many years has not been conducted, especially with the inclusion of variables that are only recently available.

An historic context shows that situations of homelessness underwent a transitionary period following the 1980s when President Ronald Regan’s administration reduced federal budgetary allocations for social services in the United States. Budget reductions included 4.8 billion to Aid to Families with Dependent Children (AFDC), now Temporary Assistance for Needy Families (TANF), 6.8 billion to Supplemental Nutrition Assistance Program (SNAP), and 700 million to Low Income Home Energy Assistance Program (LIHEAP) [6]. Perhaps most notably, there was an 85% reduction in Housing and Urban Development (HUD) allocations from just over $65 billion to around $10 billion (resulting in a 85% reduction), and it was proposed to be slashed to just 500 million but did not pass [7] (p111). The decreased overall funding among literally all welfare for the poor during the 1980s resulted in drastic increases in nationwide homelessness—an 800% increase in cities like Chicago [2]—and a shift broadening the range of demographics among homeless populations in the U.S. ever since [7–9]. Whereas the former typical image for someone experiencing homelessness was a single male who is working age, it became common in most major cities to notice homeless families, women, children, and others, which resembles contemporary homelessness [8,10]. One meta-analysis [8] describes the transformation as “The New Homelessness,” summarizing a litany of work on the phenomenon and tying the shift in homeless populations to the welfare reductions during the 1980s.

As another meta-analysis [9] put it:

*Debate over the causes of homelessness is caught up in whether the focus of research should be on structural forces that permit homelessness to occur or the immediate reasons why people become homeless. (p130)*

Advocacy following the 1980s has long called to recognize how structural variables impact homelessness in a broader scope of the issue rather than focusing on the personal attributes of individuals who experience homelessness [8,11,12]. Although many studies find high prevalence of mental health and chronic health conditions [13–15],, researchers throughout the previous forty years have advocated not to lose sight of structural explanations. Following the economic downturn in 2020, there is increased acceptance and attention toward expanding the scope of data analysis to include structural indicators in explaining homelessness beyond only those who interact with social services [5,16–25].

In their book, *Homelessness is a housing problem: How structural factors explain U.S. patterns*, Colburn and Aldern [4] examine why rates of homelessness vary between geographic locations. They elaborate by saying:

*Housing market conditions explain why Seattle has four times the per capita homelessness of Cincinnati. Housing market conditions explain why high-poverty cities like Detroit and Cleveland have low rates of homelessness. (p10)*

They liken the limited housing stock to a game of “musical chairs,” where the inadequacy of available chairs serves as a more substantial explanation for losing access to housing than does any personal issue or physical disability (p14). Their metaphorical comparison of the housing market to a game of musical chairs underlines that the scarcity of resources (not individual deficiencies) is central to understanding the issue of homelessness.

However, we believe that housing availability is not the *sole* metaphor for structural indicators in this analogy. Many additional factors can offer explanations for the fluctuations in homelessness rates across geographic location and time, such as the job market, median rent, eviction rates, occupancy rates, median income, the construction of new homes, the cost of one-bedroom units, funding from Housing and Urban Development (HUD), and policies under democrat or republican elections (by state). We aim to consider a broad collection of structural factors contributing to homelessness. We also understand that these variables play some role in homelessness rates and that they are not uniform across geographic space or time. Therefore, we consider such variables across the United States from 2007 to 2024.

### Conceptual framework

We organize the eight analytic variables along three substantive axes (Fig 1) that the structural-homelessness literature has identified as channels through which state-level conditions affect homelessness. The first axis is housing affordability, which includes median household income, median rent, eviction rate, and the cost of single-bedroom units (SBUs). The second is housing supply, which includes occupancy rate, with new-construction sales available 2018-present as a sensitivity variable. The third is the public safety net, which is represented by HUD funding (per capita; see Methods, Data preprocessing). The outcome variable is the homelessness rate (HUD Point-in-Time count divided by state population). Time appears as a network node to absorb secular trends. This grouping is a heuristic to organize variable interpretation, not a structural causal model: the Markov random field we estimate (Methods) does not assume any a priori direction among the axes; instead, it identifies which variable pairs remain associated after conditioning on the others.

**Fig 1:**
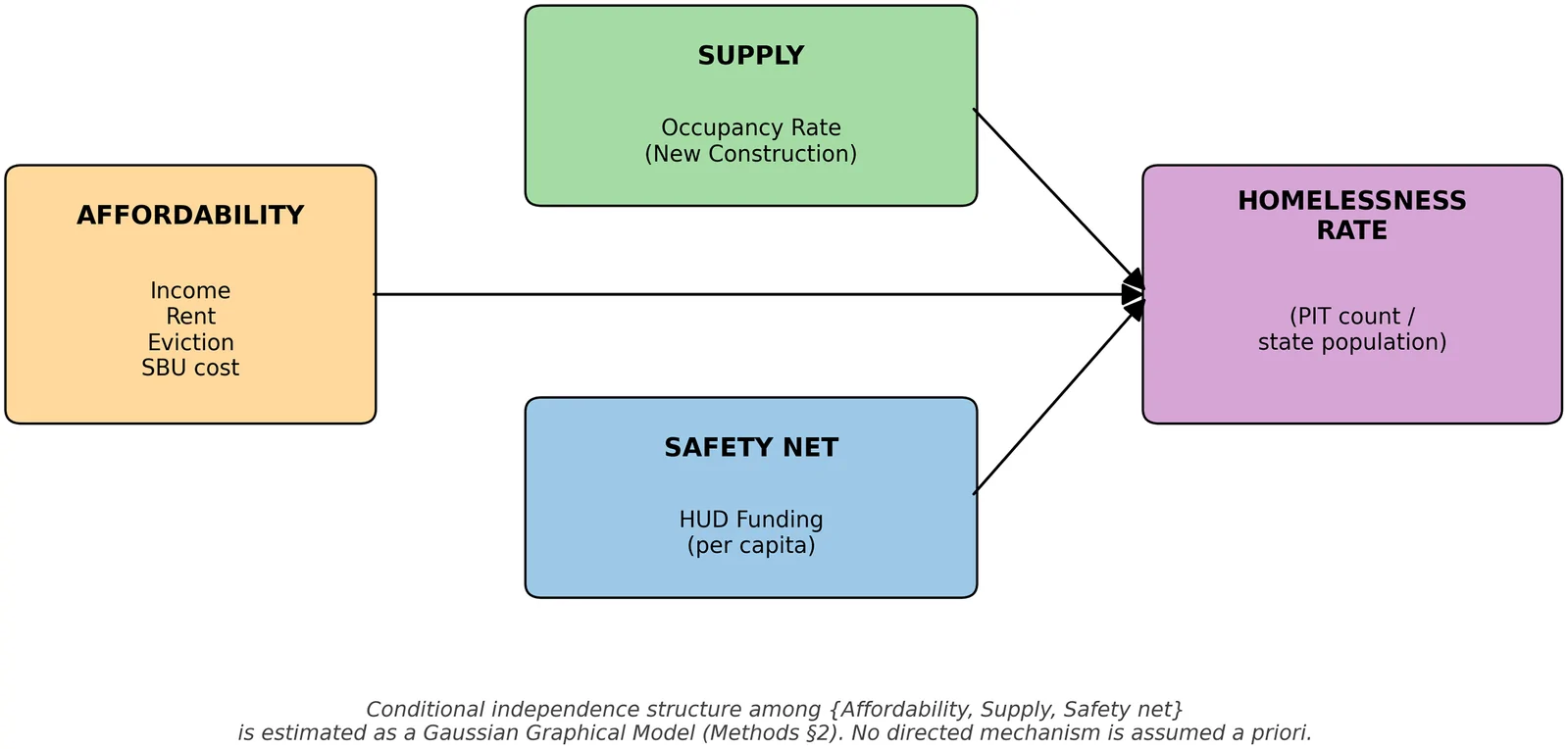
Conceptual framework: structural variables organized along three axes. The seven analytic variables are grouped into housing affordability (Income, Rent, Eviction, Single-Bedroom unit cost), housing supply (Occupancy Rate, with New Construction available 2018-present as a sensitivity variable), and the public safety net (HUD funding per capita). The outcome variable is the homelessness rate (HUD Point-in-Time count divided by state population). Time appears as a network node to absorb secular trends. The grouping is a heuristic for variable interpretation, not a structural causal model.

## Literature review

Despite the extensive history of research on the subject, homelessness levels continue to rise. In a 10-year forecast, Almquist et al. [26] say that homelessness could reach over 3 million by 2035. That would be around a 400% increase from 771,480 (see Note 1 in Appendix in S1 Appendix) in January of 2024 [3] and was calculated before President Trump’s Big Beautiful Bill reduced governmental funding for social services [27]. The Organization for Economic Co-operation and Development (OECD) says that low-income earners in the U.S. spend around 49% of their income on rent (or housing), which is the highest in the world [28]. These figures emphasize the severe housing affordability crisis contributing to homelessness in the U.S.

Research has long advocated the issue of homelessness be recognized as a byproduct of extreme poverty [12] while more recent literature on the subject has given such claims more focus. For example, recent literature finds that indicators, such as the housing market bear strong consequences on the amount of homelessness [4,18], and poverty rates are the only significant predictor of homelessness [29]. In this article, we explore potentially causal connections among such indicators through the use of Markov random fields. To the best of our knowledge, such a large-scale analysis over a substantial time horizon has not been conducted before.

Byrne et al. [30] considers how social inequalities can predict homelessness, comparing the relationship across 239 U.S. communities over a twelve-year period. They tested both “income channel” and “price channel” mechanisms, finding that a one-point increase in the Gini coefficient associates with 7.6 additional homeless persons per 10,000 population [30]. Heston [31] investigated 25 potential socioeconomic variables across all 50 states from 2020–2023 to identify the primary drivers of homelessness at the state level. They identified a cost-of-living index, which included the costs of housing, transportation, groceries, and the cigarette excise tax rate, as the dominant factor associated with homelessness (accounting for 61.4% of explanatory power), with unemployment (16.4%), alcohol use (10%), taxes (6.7%), and poverty (3.9%) as secondary structural contributors [31].

Quigley and Raphael [21] tested whether economic conditions in housing markets rather than social pathologies or mental health issues are the primary drivers of homelessness rates in the U.S. They spotlight the hypothesis that the deinstitutionalization of mental patients to the streets, an often misunderstood phenomenon that took place from 1945 to the early 1970s [32] (p3), cannot fully explain the contemporary framework on homelessness saying,

*Concerning the de-institutionalization hypothesis, the decline in mental hospital populations has been largely offset by increases in the numbers of the mentally ill confined in other institutional settings…[i.e.,] prisons and jails, and state and county mental hospital. (pp324)*

They go on to find that a 10% increase in rents in the U.S. associates with a 6.5% increase in the rate of homelessness while a 10% increase in vacancy rates correlates with a 6% decrease in homelessness, emphasizing rent as a primary cause of homelessness [21]. They analyzed four distinct datasets on U.S. homelessness, including U.S. Census data (270 metropolitan areas), shelter counts (116 metropolitan areas), official estimates for California counties, and administrative records from California's welfare system [21]. Through regression analyses, they test relationships between homelessness rates and housing market variables such as vacancy rates, median rents, and rent-to-income ratios. They found that higher rent-to-income ratios were significantly correlated with increased homelessness, and that modest changes in housing markets (increasing vacancy rates by one percentage point and decreasing rent-to-income ratios from 17.5% to 16.8%) could potentially reduce homelessness by 25% [21]. It is crucial to note these were correlational insights. In our analyses below, we control for the effect of several potential confounders and examine, how, given details on the remaining variables, two variables could impact each other in a *conditional* relationship. We explicitly state the assumptions under which a causal reading is appropriate (Methods, “Assumptions and Robustness”).

The cost of rent also plays a factor in homelessness. Glynn et al. [33] developed a Bayesian nonparametric regression model to compare homeless rates, housing affordability, and community-levels of poverty to find that homeless rates “quickly increase once median rental costs exceed 30% of median income” (p1037). Others blame the cost of living, in general. Cleveland [34] considers increasing economic disparity overall rather than housing alone, finding that the growing income gap impacts homelessness more indirectly than just focusing on housing. Eun-Gu [29] utilized multiple regression analysis to consider poverty, employment rate, recipients of Supplementary Security Income, and Fair Market Rents, 52 cities (6307 service locations offering 11,983 homeless assistance programs) in the U.S. finding poverty to have an independent and significant impact on homelessness.

The discourse on economic dimensions and labor conditions presents another layer to this complex issue. While many associate income with jobs, the relationship between the two and their impact on homelessness varies. Possessing a job does not always ensure that people can cover expenses or afford a home. Goldstone [5] addresses “the working homeless,” or those who are employed and experience homelessness. Detailing the struggles of five families in Atlanta, Goldstone focuses on how inadequate employment and low wages impact homelessness. He summarizes by saying:

*Today there isn't a single state, metropolitan area, or county in the U.S. where a full-time worker earning the local minimum wage can afford a two-bedroom apartment. Currently, 11.4 million low-income households are classified as "severely cost burdened," spending, on average, an astounding 78 percent of their earnings on rent alone. But strikingly, it's in the nation's richest, most rapidly developing cities—-the ones "doing well"— that the threat of homelessness has become particularly acute. New York, whose economy soared over the last three decades, has watched its shelter population more than triple. Washington, D.C., boasts some of the country's highest median incomes; it also has one of the greatest per capita homeless rates. Seattle is close behind. In Austin and Phoenix, Denver and Nashville, the pattern is similar. Unemployment is at a record low; corporate profits have surged; the signs of growth are everywhere. Yet the delivery drivers, daycare workers, supermarket cashiers, and home health aides who help sustain our cities are being relentlessly priced out of them—and often out of housing altogether. Unlike earlier periods of widespread immiseration, such as the recession of 2003, what we're seeing today is an emergency born less of poverty than prosperity. Families are not "falling" into homelessness. They're being pushed (xvii).*

People who work and also experience homelessness is a concept that emerges as an under researched topic. Ehrenreich [35] also studied restaurant servers and other service workers, finding that many who had formal employment were also experiencing homelessness but conceal it by sleeping in their cars between shifts, a phenomenon described as “hidden” populations of homelessness [36]. More broadly, in his seminal work *When Work Disappears: The World of the New Urban Poor*, Wilson [37] portrays how employment has disproportionately impacted people of color, particularly Black populations, due to the mass exodus of employment opportunities away from urban areas in the mid to late 20th century, which helps explain the disproportionately high rates of homelessness among black populations, and people of color [38,39].

However, even if someone has housing and can pay the rent, they may lose it still. In another seminal work, *Evicted: Poverty and Profit in the American City*, Matthew Desmond [16] shows how heavily the role of evictions play in explaining issues of homelessness in Milwaukee, WI, and across the U.S. Desmond spotlights the power imbalance between landlords and tenants, finding that tenants often accept substandard housing conditions and face minimal legal rights as they are evicted, perpetuating a cycle of “disposable” social ties and support systems. He argues that investing in public legal aid to prevent evictions can help sustain tenants housing and save public funding, substantially [16]. Since then, Treglia et al. [40] utilized eviction filings to predict homelessness in fixed effect regression analysis to find a positive association between evictions and shelter inhabitants, but not homelessness rates, overall.

The housing stock available to the population also plays a significant role in influencing housing demand and determining which segments of the population can secure housing. This is particularly evident in the impacts made by large companies, such as Blackstone, on the housing market [41]. These companies have acquired significant amounts of housing stock with the aim of realizing profits. However, these profits may only materialize after extended periods, if at all, leading to negative impacts across various types of housing options [41]. In fact, Single Room Occupancy (SRO) dwellings have historically played a crucial role in poverty and homelessness [42]. These dwellings resembled one-bedroom apartments with a shared bathroom or kitchen at a fraction the cost of a single bedroom apartment [42]. In the 1950s, however, communities across major U.S. cities sought to rid themselves of blighted buildings and undesirable tenants by revising zoning laws to eliminate SROs by prohibiting the development of new ones, which also diminished the availability of studios, and “single bedroom units” (SBUs) [42]. As SROs and SBUs continue to dwindle ever since, advocates are focusing on tenants who struggle with rent and housing, including “not being able to find enough studio and one-bedroom apartments” [43] (p1). People experiencing housing insecurity are forced to either pay increased rates for newer and more expensive SBUs or share rent in a housing market that contains an excessive number of bedrooms [43–45].

HUD has historically constructed just over 400k new units each year until the mid-1980s (around the same time as the emergence of The New Homelessness) in attempt to account for housing stock, but congress has neglected to reenact such efforts since then [46,47]. From 1994 to 2008, there has been a 19% reduction in the overall housing stock in the U.S [48]. From 2000 to 2024, housing stock also reduced by 20% [49]. As a remedy, a proposed Tax Relief for American Families and Workers Act of 2024 suggested the construction of residential units as a means to counteract the existing housing deficiency, which is subsequently contributing to the elevated prices in the contemporary market in both the rental sector and the purchasing of houses [50]. However, the problem persists because this bill did not pass in the senate [51,52]. If HUD had continued its trajectory since the decline since the 1980s, it is projected that an estimated 2.5 million SBUs would have resulted, more than accounting for the homeless population currently in the U.S. several times over [42].

Despite unsuccessful efforts in congress, the issue of housing stock has drawn increased attention. United Way [53] conducted an analysis by state of vacant units to homelessness finding that there are 28 vacant units per single person experiencing homelessness in the U.S. They found Detroit, MI, to have the most vacant homes per person experiencing homelessness, followed by Syracuse, NY, and San Jose, CA [53]. Often these units are dilapidated, or otherwise unsuitable for people to live there, but we later discuss how the owners of vacant units draw substantial tax benefits, especially compared to HUD allocations.

The construction of new homes persists as an issue in the U.S. Tamla Rai, V [54]. found that higher vacancy rates and the overcrowding of rental spaces tend to be associated with homelessness rates among families, but not individuals or youth. The cost of rent was inconclusive though [54]. Dawkins [55] focused on geographic limitations and land use policies finding that restricted housing availability, which is strongly linked to rent increases, underscores the interconnected relationship between housing supply, rent, and homelessness. Chapple and Song [56] found that in Los Angeles and San Fransisco, CA, the construction of new homes increased the likelihood of low-income renters moving into the neighborhood by 10% in Los Angeles and 15% in San Francisco for every 100 new homes constructed. Conversely, the construction of new homes also decreased displacement by 2% in Los Angeles and 14% in San Francisco [56].

Contemporary research seeks to measure homelessness beyond the shelter and service settings, thusly finding high significance in their results. In a similar spirit, we consider measuring across multiple structural indicators by state in the U.S, such as median wages, evictions, vacancy rates, new construction of homes, and homeless rates to explain which variable(s) impact homelessness the most.

## Methods

In this section, we aim to achieve two goals. We look at the mutual interplay among seven variables (which is more than several prior state-level analyses (see Table 1) that could impact homelessness rates. Additionally, we bring out potential causal links among key variables that policymakers can use to combat the problem by utilizing the latest statistical tools, along with highlighting which links vary substantially across political aisles, so further questions can be raised on why strengths of certain links differ substantially across states.

**Table 1:** Comparison with prior state-level and community-level studies of homelessness.

Study	Spatial unit	Period	Method	Contribution
Quigley & Raphael (2001) [21]	50 metros	1990	OLS regression	Housing-market conditions as primary drivers of MSA-level homelessness
Byrne et al. (2013) [30]	381 CoCs	2007-2010	Multilevel regression	Community-level determinants; role of unemployment and rent
Heston (2019) [31]	50 states	single cross-section	OLS with 25 predictors	Broad state-level covariate scan; correlation with PIT count
Glynn et al. (2021) [33]	381 CoCs	2007-2020	Bayesian nonparametric regression	Homelessness, affordability, poverty relationship; CoC-level
This study	50 states + DC	2011-2024 (varies)	Gaussian Graphical Models; NCT; cluster permutation; lag-1 DAG	Conditional-dependence mapping; political stratification with panel-respecting inference

Several studies have examined structural predictors of homelessness at various levels of aggregation. Table 1 situates the present analysis alongside four of the most relevant. Our contribution is not primarily the breadth of covariates (Heston, for instance, considers 25 state-level variables, and Glynn et al. examine 381 Continuums of Care) but the use of conditional-dependence networks (Gaussian Graphical Models) to map the joint structure of the variables, the Network Comparison Test with cluster permutation to compare political strata, and the exploratory lag-1 directed specification to probe temporal ordering.

We have found Markov random fields (described below) to be useful with the goals above. Markov random fields (MRFs) are undirected networks revealing connections among variables (taken as nodes) whose edges may be taken as showing the extent of conditional dependence between two nodes assuming the other nodes do not interfere with this relationship. We used the tool in some of our earlier works in a different context. Bhaduri [57], Zahirodini and Bhaduri [58], for instance, use MRFs to test whether people's attitudes towards certain business practices (making profits, reducing wage gaps between CEOs and other employees, etc.) are causally or conditionally affected by their attitudes on other practices (caring about the climate, workers’ welfare etc.), which reactions are conditionally independent, whether the strengths of connections change as the survey-takers’ political leaning switches. These contexts closely mirror our current setting, once we agree to update the operating variables to match our homelessness study. Therefore, we devote some energy next to understand how these random fields work.

### Conditional Dependence

In any study, when several variables interact, the impact on any one is generally observed as the joint result of all others. It is difficult to separate out the pure dependence one variable has on another from this entangled effect that emerges. Conditional dependence between the two variables, after we *control for the effect of the other variables* is called for. In certain applications, what is more worrying is the presence of lurking variables which, if not controlled for, may reverse the expected relationship between the two study variables generating counterintuitive hypotheses. Partial correlations (as opposed to ordinary ones) help estimate these conditional dependencies and, in this section, we examine a small-scale data to clarify what we mean by “controlling for the effect of the other variables.”

Fig 1a shows, on the horizontal axis, how long 200 students studied the day before a major exam, and on the vertical, how many points they scored on the test the next day. One would normally expect these two steady variables “hours studied” and “points scored” to be positively correlated. Higher scores typically accompany more hours spent on studies. Fig 1a seems to suggest an opposite pattern. In fact, if one calculates the ordinary (Spearman's) correlation coefficient between “hours” and “scores” it will be −0.066. On investigation, the presence of one or more *lurking* variables may be suspected – whose details were ignored on the previous graph and the correlation calculation. One could be the inherent ability of the students we are studying. Students with, say, familiarity with the topic or who are otherwise, in general, more adept at the subject – shown with green dots on panel B – may study less and still score well while the rest – shown with red dots – may study more but still receive lower scores on an average. Panels a and b otherwise, show the exact same dots. Interestingly, however, if we look into the green and red groups separately, the “right” answer comes out from each: that studying more is generally accompanied by increased scores. The act of moving from panel a (when we were unaware of the lurking variable) to panel b (when we are aware of it) is called “conditioning on the lurking variable”, ability, in this case, or controlling for it [59]. The exercise demonstrates how conditioning could help with our confusion around counterintuitive correlations like −0.066 by offering more intuitive ones: 0.829 from the green group, and 0.1189 from the red (Fig 2).

**Fig 2:**
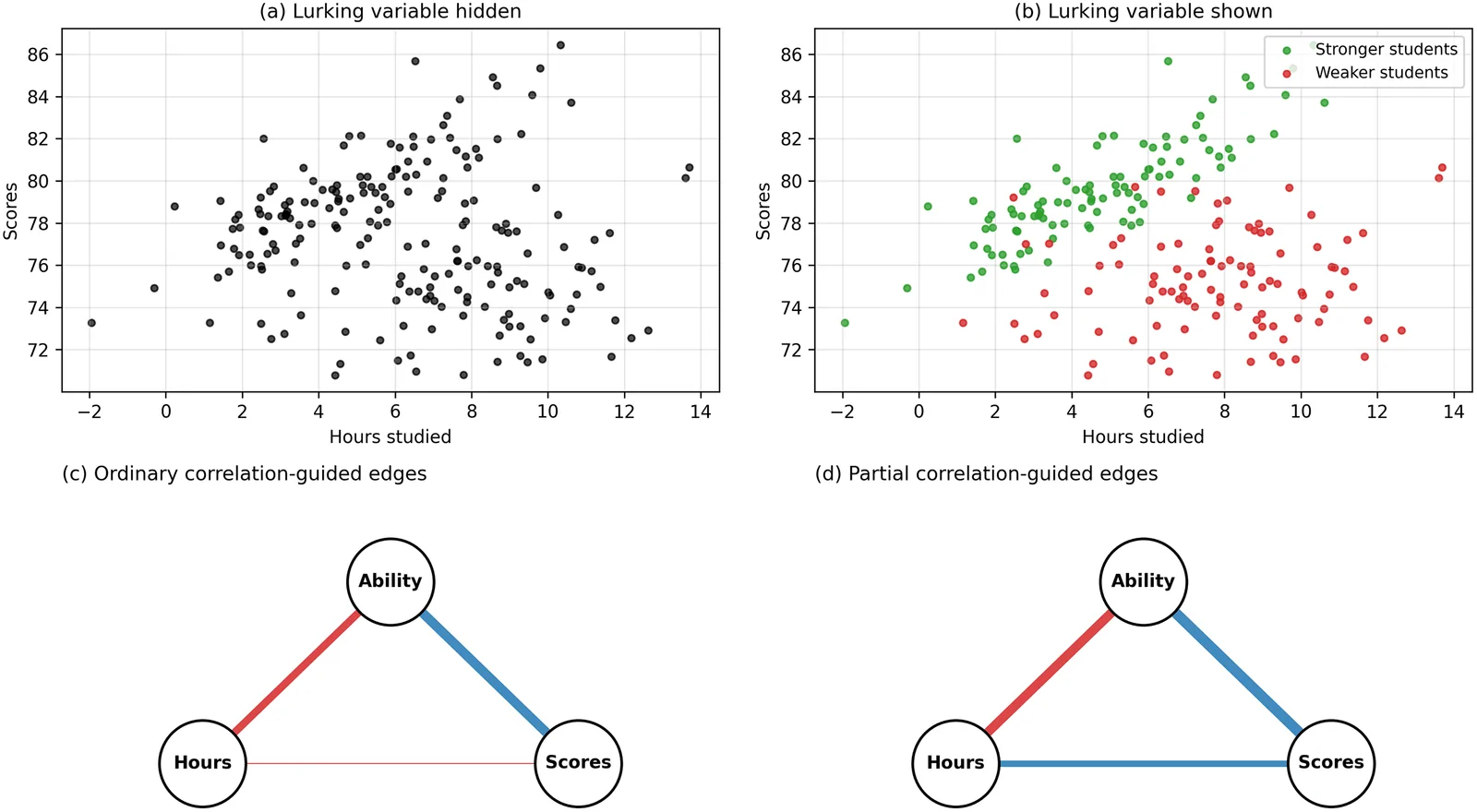
Effect of conditioning on a lurking variable: a synthetic example. (A) Marginal scatterplot of study hours versus test scores for 200 students; the marginal correlation is −0.066, contrary to substantive expectation. (B) Same data colour-coded by student ability; within each ability group the relationship is positive. (C) Network drawn from ordinary correlations: Hours and Scores appear faintly inversely related. (D) Network drawn from partial correlations: once Ability is conditioned on, Hours and Scores are strongly positively related, and the spurious negative edge disappears.

The partial correlation between two variables is the Pearson correlation between the residuals obtained by regressing each of them on all the others. Equivalently, if K is the precision matrix (the inverse of the covariance matrix), the partial correlation between variables i and j is -K_ij / sqrt(K_ii K_jj). For jointly Gaussian variables the two definitions coincide, and an absent edge in the network corresponds to K_ij = 0, which is the conditional-independence statement. In our case, scores from a previous tests are taken to represent the ability of these students and the partial correlation between each pair is shown in the right panel of Table 1 once the other variable is controlled for. This matrix is converted into the network – an MRF – shown in Fig 1d with positive partial correlations shown in blue, negatives in red; the thickness growing in relation to the strength. It shows how, for instance, “hours” and “scores” are strongly positively dependent once we control for the effect of “ability”. For comparison, a similar network constructed with ordinary correlations (left panel of Table 2 or Fig 1c) would – as seen previously – give out a wrong impression of “hours” and “scores” being inversely connected through the faint red edge joining these two variables. In our main analysis on homelessness, we construct networks similar to Fig 1d using partial correlations to examine true connections between pairs of study variables controlling for the effect of several others.

**Table 2:** Ordinary and partial correlation values to create the MRFs shown in Fig 2. Ordinary pairwise correlations are counterintuitive, while partials are logical.

	A) Ordinary correlations			B) Partial correlations		
	Hours	Score	Ability	Hours	Scores	Ability
Hours	0	−0.066	−0.527	0	0.421	−0.63
Score	−0.066	0	0.665	0.421	0	0.729
Ability	−0.527	0.665	0	−0.63	0.729	0

### Data sources and variables

This study utilizes a variety of publicly available datasets spanning from 2007 to 2024, subject to availability constraints for specific metrics. The data have been systematically organized into a publicly accessible dashboard, along with a data dictionary that walks readers through the process to select each variable via each respective web source. Due to the variation of sources, the temporal scope for certain variables is limited, as U.S. Census data from the source we utilized extends back to 2010 (see Note 2 in Appendix in S1 Appendix) while New Construction data is only available post-2018.

Census Data: The analysis employs datasets extracted from the American Community Survey (ACS) [60]. The variables under consideration—Total Housing Units, Occupied Housing Units, Vacant Housing Units, Median Rents, Population, Single-Bedroom Units (SBU) (see Note 3 in Appendix in S1 Appendix) unit costs, and Median Household Income—are organized by state using single-year estimates for comparison of each year, individually (see Note 4 in Appendix in S1 Appendix). The extraction process involved filtering variables, such as Median Household Income, by state, categorized under “income and poverty.” In instances where data scraping was time-intensive (i.e., population and median income), Python scripts facilitated the process (please see Appendices A through C), with manual verification conducted across all states and years to ensure accuracy. SBU cost was obtained by geographic type (state) “housing characteristics”, then 1-year estimates.

New Construction (Sales Count): The data comes from Zillow [61], listed under “New Construction” section. Data was organized according to state by township for each month, so we aggregated each available state that was listed and month to get a sum for the total year. Sales were utilized, encompassing information from 2018 onwards, aligning with the methodological constraints identified earlier.

Eviction Rates: Data relevant to eviction rates were sourced from the Evictions Lab at Princeton University, as reported by Desmond [62]. These rates are already calculated by state and year.

HUD Funding and Homeless Count: Data on Housing and Urban Development (HUD) funding followed a comprehensive scraping of the HUD Exchange website [3] for all HUD funds by state as of 2025. Although technically allocated by the federal government, these financial data points are integral to understanding the fiscal landscape impacting homelessness at the state level.

HUD PIT data. All data comes from the HUD Exchange website. Data was scraped from each year at the state level, except 2021 when HUD stipulated that states’ participation in the annual PIT was optional due to health concerns regarding COVID-19.

We used income to be a more informative variable than jobs for a few reasons. Utilizing employment variables is problematic and complex for many reasons. Regardless of one’s job, their income may not be enough to pay the rent. For example, Ehrenreich [35] and Goldstone [5] spotlight workers who experience homelessness because they cannot afford rent, despite having a job. Hudson [63] found that since 1975, one third of the workforce has shifted from full-time work, including benefits, to part-time or nonstandard work, which has only worsened since the gig economy [64]. In Kalleberg’s [65] book, *Good Jobs, Bad Jobs: The Rise of Polarized and Precarious Employment Systems in the United States 1970s to 2000s*, Kalleberg recognized the litany of jobs that may be available to the public, while also acknowledging how employment does not equate to decent livelihood or the prevention of homelessness. There are additional issues with utilizing traditional employment rates, such as acknowledging the growing prison population, which omits prisoners from analysis despite being forced to work for little to no income and understates Black-White employment discrepancies by as much as 45% [66]. Hacker [67] also shows that there is decreased stability among the workforce and that employment since the 1970s has been more precarious, particularly among lower-level workers in relationship to top management positions. Traditionally, general employment measures also neglect to delineate between part-time and full-time work. For these reasons, we see income as a better indicator than employment.

Political Context: Information on state-level political party affiliations and any transitions therein were deduced from data from Kaggle.com called “US Governor Dataset” (Conrady, Brandon [68]. This information encompasses Party Switch Check and Party Switch Date metrics, facilitating an analysis of political impacts across the temporal spectrum. After checking the data via the National Governors Association [69] to find that is was mostly clean (only one governor had the wrong date: William James Samford), we then added 2 columns, one called TookOffice_Year that extracted the year from the original and LeaveOffice_Year to extract the year from original LeftOffice date column, which supplied our dashboard with the terms of each governor and the dates that the political party changed.

We standardized the collection and analysis of data across these varied sources, employing comprehensive validation techniques to ensure the reliability and accuracy of derived insights. ACS data for one year in 2020 does not report single-year estimates due to COVID-19, so we utilized five-year estimates for housing units for this year only. The same is true for median rents and population in 2020. These are the best data available for 2020. Our data came from various sources but are all measured at the state-level in the United States for consistency. The variables are as follows: median household income, HUD funding, new construction, eviction rate, SBU dwellings, and were compiled into a spreadsheet in Microsoft Excel.

### Data preprocessing

To avoid confusion between the several sub-samples used across the paper, Table 3 lists each network specification, its sample window, sample size (n = state-years), the number of unique states contributing, and the variables entering the specification. The abstract's 2007–2024 range refers to the outer bounds of data availability across all analyses; individual specifications use narrower windows determined by variable coverage. In particular, the seven-variable main network is restricted to 2011–2018 because state-level eviction rates are systematically recorded only through 2018 in the source database.

**Table 3:** Analytic samples across the paper.

Analysis	Window	n	States	Variables	Reported in
Pooled headline GGM	2011-2018	399	50	7 (Income, HUD per cap, Eviction, SBU, Occ, Rent, Homeless)	Fig 3, Table 4
FE-residualised, pooled	2011-2018	399	50	7 (as above, residualised on state + year FE)	Fig 4A, Appendix D
FE-residualised, Republican-led	2011-2018	245	38	7 (as above)	Fig 4B
FE-residualised, Democratic-led	2011-2018	147	26	7 (as above)	Fig 4C
Lag-1 directed network	2011-2018	348 pairs	50	7 x 2 (contemporaneous + lag-1)	Fig 5, Fig 6, Appendix G
6-variable extended (drop Eviction)	2007-2024	458	51	6 (Income, HUD per cap, SBU, Occ, Rent, Homeless)	Appendix E
8-variable with New Construction	2018-2019	86	43	8 (headline 7 + NC; Eviction dropped for 2019 obs)	Appendix E
Republican vs Democratic NCT	2011-2018	245 + 147	38 + 26	7 (headline set)	Table 5

**Fig 3:**
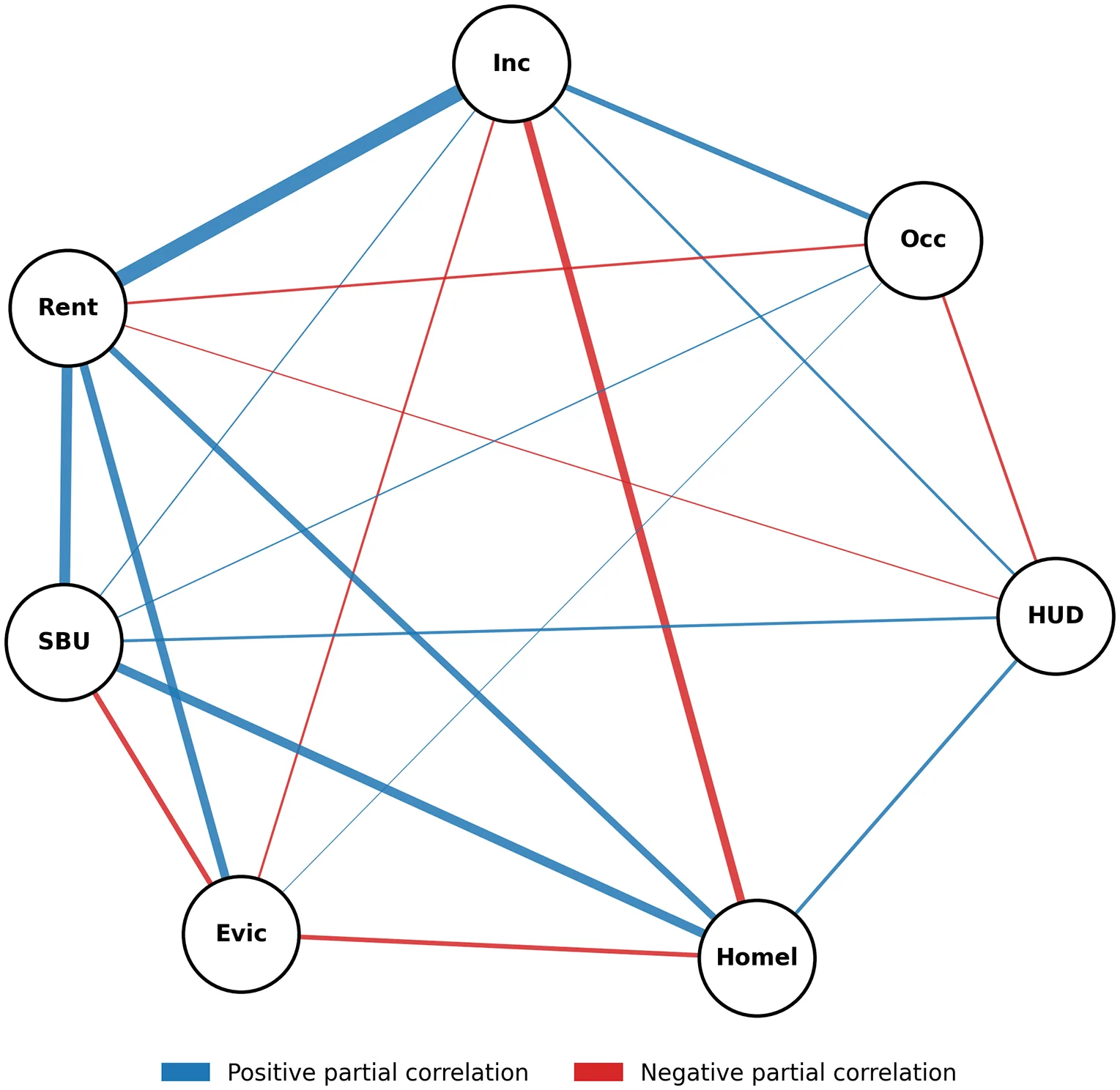
Pooled Gaussian Graphical Model, all states, 2011-2018. EBIC-tuned graphical lasso on seven standardised variables (Income, HUD funding per capita, Eviction, Single-Bedroom unit cost, Occupancy Rate, Rent, Homelessness Rate); n = 399 state-years from 50 states+DC. Blue edges denote positive partial correlation, red denote negative; edge thickness is proportional to |partial correlation|.

**Fig 4:**
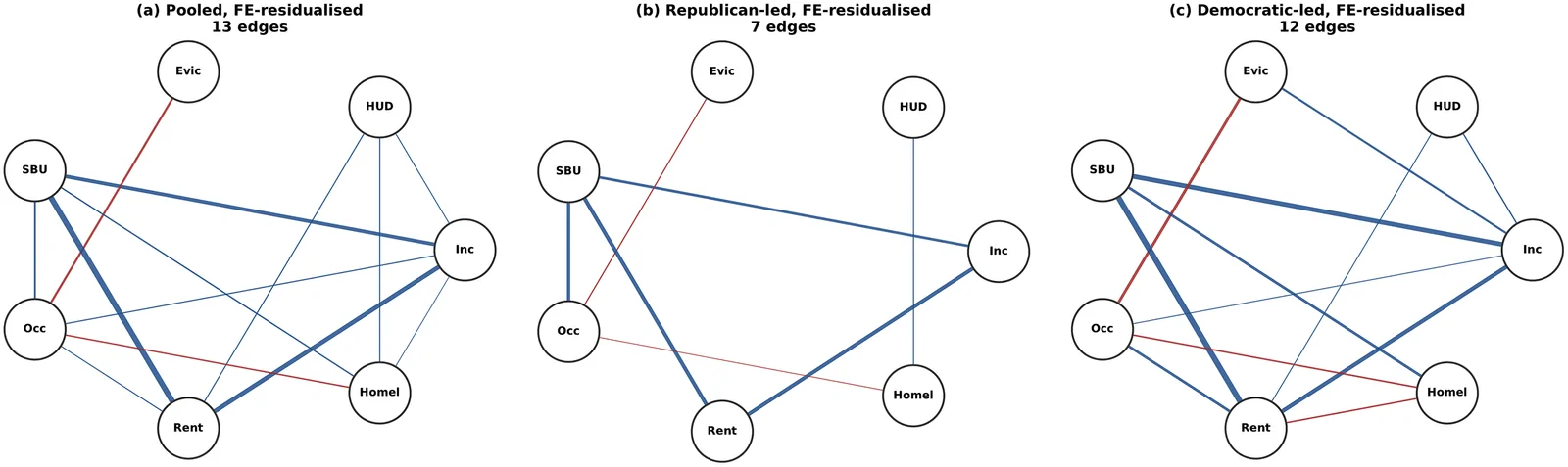
Headline FE-residualised networks, three strata. (A) Pooled 2011-2018 panel (n = 399, 13 edges). (B) Republican-led state-years (n = 245 from 38 states, 7 edges). (C) Democratic-led state-years (n = 147 from 26 states, 12 edges). Each variable is residualised on state and year fixed effects before estimation; edges therefore describe within-state, year-adjusted partial associations rather than cross-sectional level differences.

**Fig 5:**
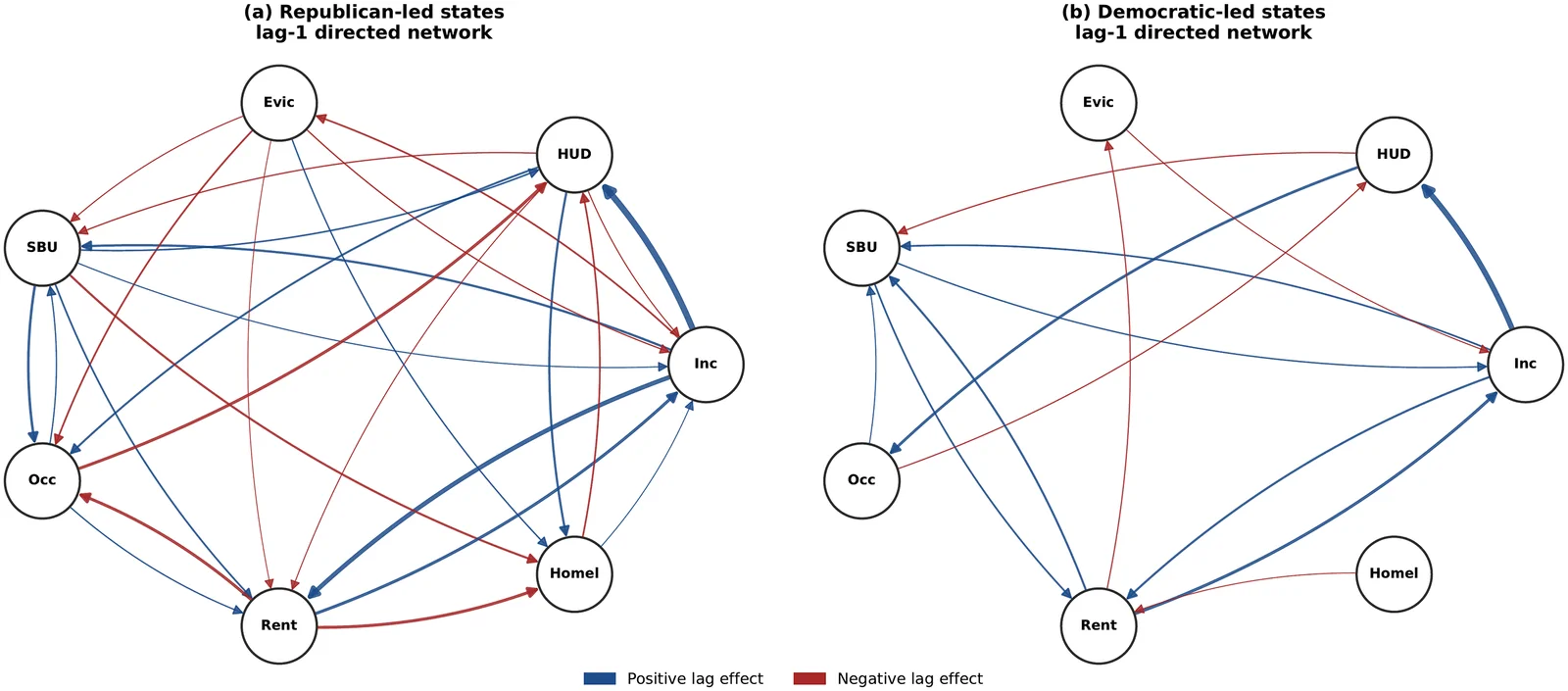
Lag-1 directed temporal network by governor party. (A) Republican-led states: nine feedback cycles, with a closed HUD < - > Homelessness loop. (B) Democratic-led states: four feedback cycles; the HUD < - > Homelessness loop is absent. Each directed edge represents a within-state lag-1 partial regression coefficient at |beta| > 0.01, BIC-tuned LASSO. Edge thickness is proportional to |coefficient|; blue = positive, red = negative.

**Fig 6:**
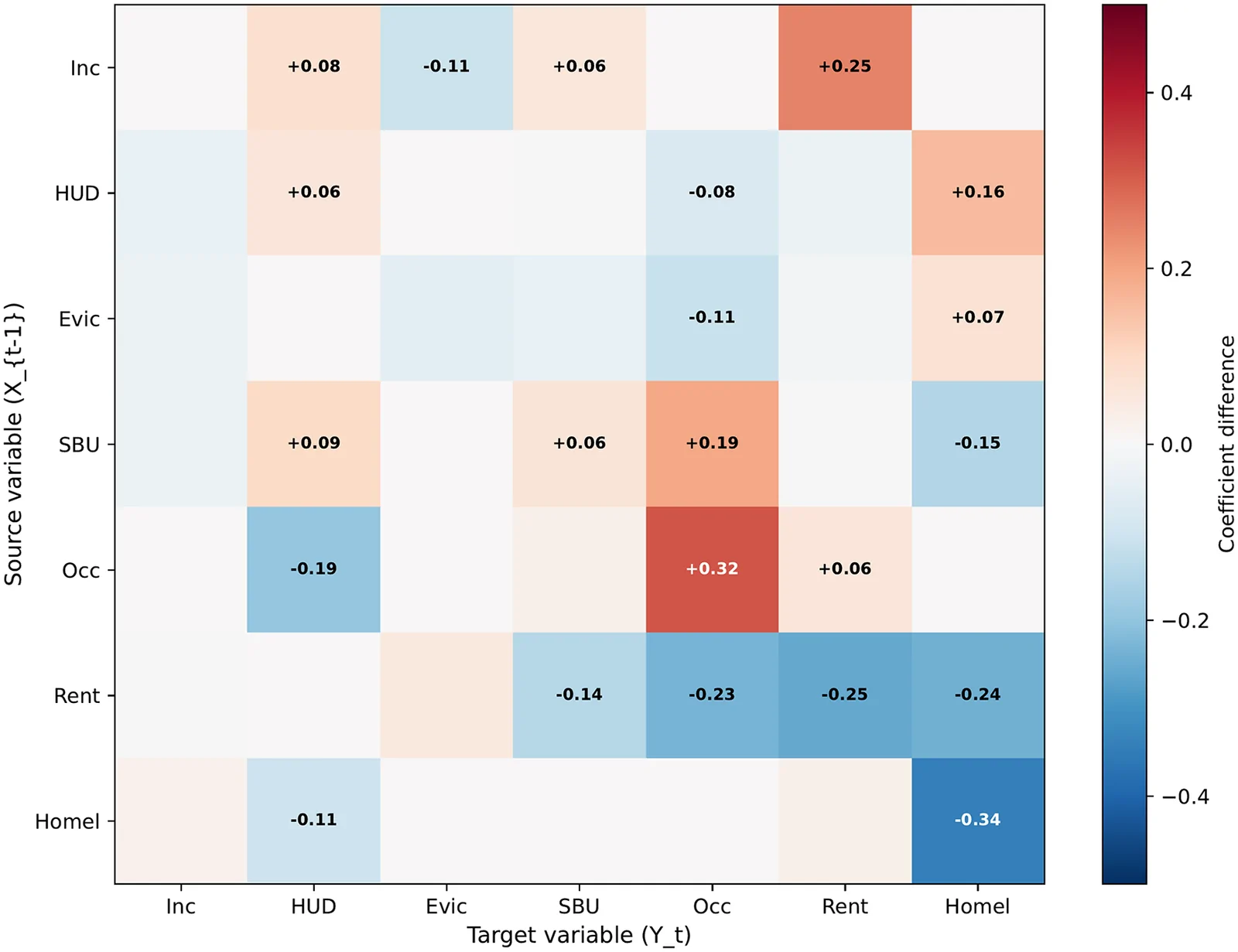
Republican-led versus Democratic-led lag-1 coefficient difference. Each cell shows the difference in the lag-1 effect of variable i at year t-1 on variable j at year t between the two strata (Republican minus Democratic). Red cells indicate stronger effects in Republican-led state-years; blue cells indicate stronger effects in Democratic-led state-years. Cluster-permutation p-value for the joint difference is 0.12 (suggestive, not conventionally significant).

Two preprocessing steps deserve explicit mention. First, HUD funding is reported per capita (HUD dollars divided by state population) rather than as raw dollars. The raw allocation correlates with state population at r = 0.94 in our panel, so the original raw-dollar specification is dominated by state-size confounding; per-capita rescaling materially changes the partial correlation between HUD and the homelessness rate (it becomes a small positive edge after rescaling, reversing a near-zero result obtained with raw dollars) and changes the sign of the HUD–occupancy edge. Per-capita rescaling is propagated through every specification (baseline, FE-residualised, nonparanormal, FCI, NCT). Second, all variables are standardised to zero mean and unit variance prior to estimation; this matters because HUD per capita (~$50/person) and median rent (~$1,000/month) operate on radically different numerical scales. Standardisation puts the partial correlations on a common scale and is required for the EBICglasso tuning to behave sensibly.

Listwise deletion is used in the headline 2011–2018 seven-variable specification (n = 399 complete state-years from 50 states); a six-variable specification dropping Eviction extends the panel to 2011–2024 (n ≈ 700), and multiple-imputation results for the eviction series 2019–2024 are reported as a sensitivity check. Reproducibility materials — a single self-contained R script (HousingMRF.R) that regenerates every figure, table, p-value, and bootstrap interval reported in this paper from the publicly archived input files — are provided as Supporting Information.

### Temporal structure

Pooled state-year observations are not independent: states reappear in multiple years, and most of our variables are highly persistent. We compute within-state lag-1 autocorrelations and Augmented Dickey-Fuller [70] stationarity tests for each variable. Income, rent, and single-bedroom-unit cost have mean lag-1 ρ ≈ 0.95 and almost no within-state series is stationary at 5%; eviction rate and homelessness rate are persistent in most states; HUD funding (per capita) is the only variable that is volatile from year to year. We also decompose the variance of each variable into between-state and within-state components and find that 84% to 99% of variation in every variable is between states. The pooled descriptive network is therefore overwhelmingly identified by between-state heterogeneity rather than within-state covariation.

Two consequences follow. First, treating state-years as exchangeable observations — the implicit assumption of the ordinary bootstrap and of the pooled GGM — will overstate the effective sample size. We address this by reporting the cluster bootstrap by state (resampling whole states, not state-years) as our preferred uncertainty quantification (see “Assumptions and Robustness” below). Second, partial correlations estimated on the raw pooled panel measure level differences between states rather than within-state covariation. We therefore re-estimate the network on residuals from a regression of each variable on a full set of state dummies and a full set of year dummies; this two-way fixed-effects residualisation absorbs time-invariant state-level confounding (geography, climate, zoning history, demographic composition) and common annual shocks. The resulting FE-residualised network is reported as our primary specification; the pooled descriptive network is reported alongside as context. The Time node is dropped automatically when FE residualisation is applied because year fixed effects subsume it.

### Estimation and implementation

We estimate the precision matrix and the partial-correlation network via the graphical lasso [71] implemented in R via qgraph::EBICglasso, called from bootnet::estimateNetwork(default = “EBICglasso”) [59]. The regularisation parameter λ is selected by minimising the Extended Bayesian Information Criterion of Foygel and Drton [72]; we use the bootnet default tuning parameter γ = 0.5. The Network Comparison Test of van Borkulo et al. [73] is implemented as NetworkComparisonTest::NCT() with it = 1000 iterations. The cluster bootstrap by state uses B = 100 replicates per stratum. All seeds are fixed in HousingMRF.R; results are reproducible to the bit.

## Findings

Fig 3 shows the Markov random fields spanned by partial correlations among these eight study variables for all states from 2007 to 2024. As in the earlier section, the partial positive connections, once we control for the effects of the remaining variables, are shown in blue. The “Income-Rent” connection is a case in point. For all states, we observe these two variables conditionally covary: higher values of one usually accompany higher values of the other. The high partial correlations contribute to the heavy thickness of the “Income-Rent” edge, while the positive nature of dependence is the reason they are shown in blue. In contrast, negative partial correlations, after the controlling, are shown in red. “HUD funding received - Homelessness rates” is an example. For all states, they conditionally anti-vary (or an inverse relationship): higher values of one usually accompany lower values of the other. The more HUD allocations are distributed to states, the lower their homelessness rate.

Our novel analysis shows various impacts on homelessness rates and compares each of our collective structural indicators. Fig 3 shows all relationships and their impact on each other with thicker lines meaning the most impact. Focusing on homelessness rates, we see that there are six main relationships with other variables, which are in order of their highest impact (see Fig D2 (Appendix D in S1 Appendix)): single bedroom units (SBUs; see Note 5 in Appendix) (+.5 significance), rent (+.1), time (−.25), eviction (−.2), HUD funding (−.2), and income (−.2) (in Fig D3 (Appendix D)). The significance between homeless rate and SBUs is the second highest only behind the relationship between income and rent. Therefore, we can also say that rent *among* SBUs is also among the highest impact (see Fig D3 (Appendix D)). In Fig 3, we can see that as rent increases, evictions increase, occupancy rates decrease, homelessness rates increase, and the need for HUD funding increases. SBUs’ impact on rent is the second highest significant relationship overall, behind the relationship between income and rent, and has the highest impact on the homeless rate.

The relationships in Fig 3 that have invisible lines between variables show us that they have no real impact on each other given the impacts of the other variables in our sample. For example, under this conditionality, we show that homeless rate and occupancy rate have no relationship (compared to the other variables we have considered). Evictions also bear no direct relationship with occupancy rates. It seems that what landlords charge for rent has more *direct* impact.

As we focus on more granular analysis under “subset analysis: states by political party”, we show how these connections update as we bring in details on which political party was in power. In general, on diagrams such as 3 and 4, we report which factors, in general, strongly impact the rest and how reliable connections among variables are: the narrower the gray bands the more reliable.

For instance, the dependence between income and rent or between eviction and rent are reliable, while the one between occupancy rate and homelessness rate is not so. These gray areas enable one to use caution while interpreting one-step conditional dependencies. For example, although the partial correlation between occupancy rates and homelessness rates is marginally positive, a wide gray area points out we are not as sure with this result as we are as with eviction-rent.

We now elaborate further to explore additional findings in our analyses, then delineate subset analyses pertaining to the construction of new housing units and political party.

### Subset Analysis: “New Construction”

As stated previously, the data available for “new construction” was limited to two years (2018–2019, the coverage available in the source) and we cannot do a subtler analysis as we have done in the following section because there would be a dearth of data to give reliable estimates for the causal links. However, we believe that four years of data across all 50 states adequately captures the new construction variable, which significantly impacts (0.792 significance) discussions on the topic, which is higher than HUD funding (0.756), evictions (0.571), and occupancy rate (0.61) but not as high as rent (1.731), income (1.253), and SBUs (1).

We can see that over the two years under survey in this section, conditional on the rest (i.e., given details on the other variables), new construction makes rent increase rather than decrease.

### Subset Analysis: States by Political Party

In this section, using methods outlined in the previous on a smaller, intuitive example, we record the interplay between eight socio-temporal variables: eviction rates, SBUs, rent, homelessness rates, occupancy rates, time, HUD funding received, and income to check (using statistical hypothesis tests) whether the true dependence between pairs of variables vary substantially between Republican and Democrat-run states. The true dependence, i.e., the pure connection between two variables will be calculated by controlling for the effects of the other six, just as in the partial correlation between “hours” and “scores” in the previous section, we controlled for the effect of “ability” to recover the correct nature of dependence between hours studied and scores received. While these other six conditioning variables, in each case, will represent a substantial and vital chunk of crucial socio-economic indicators, we acknowledge there could be more. In our follow-up studies, we can examine whether any of these others merit inclusion or, if – which they believe to be true – many of these others are already correlated with those included here (see Note 6 in Appendix).

Several observations are in order: (i) Fig E2 (Appendix E) shows the Markov random fields spanned by partial correlations among Republican (left panel) and Democrat (right panel) run states. For both types of states, we observe these two variables conditionally covary: higher values of one usually accompany higher values of the other. Again, the high partial correlations contribute to the heavy thickness of the “Income-Rent” edge for both types of states, while the positive nature of dependence is the reason they are shown in blue. In contrast, negative partial correlations, after the controlling, are shown in red. “HUD funding received – Homelessness rates” is an example. For both types of states, they conditionally anti-vary (or an inverse relationship): higher values of one usually accompany lower values of the other. In this preliminary observation, we notice agreement: “Income-Rent” dependencies are positive for both state types and “HUD funding received – Homelessness rates” dependencies are negative for both state types. However, we point readers to observation below where we quantify the degree of these agreements through permutation tests. We will find there is no substantial difference in the blue-ness of the “Income-Rent” edges across political parties, but there is a statistical difference in the red-ness of the “HUD funding received – Homelessness rates” edges. At this level, it is enough to interpret this as the deepness of the blue in the former comparison is similar, while the deepness of the red in the latter is dissimilar. For Democrat-run states, HUD funding received and homelessness rates anti vary even more strongly (deeper red) than they do in Republican-run states (fainter red), though in both cases, they do and anti-vary (shown through reds). Some of the results are intuitive. “Time-Income” connections are positive for both state types (and we will see how this is an agreement in the statistical sense as well) which confirms over the passage of time, in general, income levels have gone up. Empty connections represent conditional independence.

Secondly, under specific assumptions stated below in Assumptions and Robustness, these Markov random fields may be taken as tools representing causal (as opposed to correlational) connections (Epskamp et al. [59]. This is especially true if the conditioning is done on the large or relevant set of potential confounders and the investigators query which nodes exert the strongest pull (blue) or repulsion (red). These are the factors to which the deepest colors are attached (blue or red) and changing those will create the largest (causal) ripple effect on the other indicators. Fig D2 (Appendix D) records those crucial nodes for Republican (left panel) and Democrat (right panel)-run states. In decreasing order of their causal impact-generating potentials, these are

*Republican states: {Rent, Income, Homelessness rates, HUD funding, Time, Occupancy rates, Eviction, SBUs}*

*Democrat states: {SBUs, Income, Homelessness rates, Rent, HUD funding, Time, Eviction, Occupancy rates}.*

We note high agreement between the state types over the top four or five nodes with the exception of SBUs: for Republicans, it exerts the least impact; for Democrats, the most. Income, Homelessness rates, HUD funding, and Rent routinely feature among the top causal variables regardless of the party.

We note these MRFs do not describe the *direction* of causal flows. For instance, from Fig 3, we cannot claim either higher “Income” conditionally causes higher “Rent”, or the other way around; only that there exists a potential direct conditional causal relationship between the two. We note the MRFs are constructed out of collected data and hence are subject to sampling fluctuations. We can quantify the volatilities or uncertainties associated behind each edge strength through Fig D3 (Appendix D). The solid red dots represent the partial correlations found from the observed data with which the edges in Fig 3 were constructed. The gray areas represent the limits of these statistics found through bootstrap replicates (data similar to the observed) with the understanding that the wider the gray area, the more unsure we become about the partial correlation that got estimated, the narrower, the surer. The lengths may be a function of the sample size. We note for Democrat-run states, this estimation is often stronger (that is, triggered narrower gray areas) than for Republican states. This is true, for instance, for “Income-Occupancy Rates” or “SBUs- Homelessness Rates”, though for both states types the connections are positive. We take these random fields to be conditional networks where the interaction between two nodes are viewed given the details of the others.

Frequently, we intend to check whether the total amount of blue and red connections match across the two networks. This is part of testing the (statistical) equality of the two networks, one formed out of Republican and another formed out of Democrat-run states. This is, in spirit, similar to testing the inequality of IQ scores across genders. However, in non-Euclidean settings such as networks, these are implemented through permutation tests (van Burkulo et al. [73]. We begin by recording an appropriate statistic – the absolute difference in this case between the “HUD funding-Homelessness rates” edge weights from the Republican and Democrat sides. The conditional edge dependency described in the previous section between these two nodes was estimated (through lasso regression) to be −0.091 for the Republican states (the fainter red color on Fig 2a or Table 4) and −0.325 for the Democrat side (notice the deeper red on Fig 2b or Table 3). The absolute difference between the edge weights therefore becomes |−0.091 – (−0.325)| = 0.235. serving as the test statistic to test for the quality of this edge strength across politics. Next, a benchmark, i.e., a null distribution is made by randomly assigning states to parties and we measure the “HUD funding-Homelessness rates” edge weight differences. Only 1.98% of those values exceeded the observed statistic which makes this 0.235 unlikely to be a chance difference (please see Table 5). Conservative and liberal states genuinely seem to disagree on how automatic the “HUD funding-Homelessness rates” connection is. What we checked is known in literature as the hypothesis for *invariant edge strength*. Formally, the null hypothesis here is

**Table 4:** Partial correlations from Republican-run states.

Baseline EBICglasso (raw) – edges = 25, lambda = 0.0077								
	Time	Income	HUDFunding	Eviction	SingleRoom	OccRate	Rent	HomelessnessRate
**Time**	0	0.235	*−0.0724*	*−0.1818*	0	*−0.2321*	0.1523	*−0.4364*
**Income**	0.235	0	*−0.2716*	*−0.0944*	0.2622	0.4604	0.5713	0
**HUDFunding**	*−0.0724*	*−0.2716*	0	*−0.1271*	*−0.1668*	0.1724	0.4775	*−0.1414*
**Eviction**	*−0.1818*	*−0.0944*	*−0.1271*	0	*−0.1738*	0	0.4657	*−0.2937*
**SingleRoom**	0	0.2622	*−0.1668*	*−0.1738*	0	*−0.1544*	0.33	0.013
**OccRate**	*−0.2321*	0.4604	0.1724	0	*−0.1544*	0	*−0.1508*	*−0.2964*
**Rent**	0.1523	0.5713	0.4775	0.4657	0.33	*−0.1508*	0	0.3551
**HomelessnessRate**	*−0.4364*	0	*−0.1414*	*−0.2937*	0.013	*−0.2964*	0.3551	0

**Table 5:** Significantly different edges across political splits are highlighted in red. The p-values are derived from permutation tests.

	Variable 1	Variable 2	p-value	Test statistic E
9	Time	Income	0.514851	0.053041
17	Time	HUDFunding	0.485149	0.063028
18	Income	HUDFunding	0.267327	0.097823
25	Time	Eviction	0.346535	0.104482
***26***	***Income***	***Eviction***	***0.009901***	***0.283903***
27	HUDFunding	Eviction	0.178218	0.125859
33	Time	SingleRoom	0.485149	0.071696
***34***	***Income***	***SBUs***	***0.019802***	***0.207999***
***35***	***HUDFunding***	***SBUs***	***0.009901***	***0.441071***
36	Eviction	SingleRoom	0.534653	0.059836
41	Time	OccRate	0.138614	0.13231
42	Income	OccRate	0.158416	0.144324
43	HUDFunding	OccRate	0.158416	0.103384
44	Eviction	OccRate	0.673267	0.022562
45	SBUs	OccRate	0.138614	0.094617
49	Time	Rent	0.356436	0.096371
50	Income	Rent	0.960396	0.003064
***51***	***HUDFunding***	***Rent***	***0.009901***	***0.405332***
***52***	***Eviction***	***Rent***	***0.009901***	***0.274969***
***53***	***SBUs***	***Rent***	***0.009901***	***0.299826***
***54***	***OccRate***	***Rent***	***0.049505***	***0.19171***
57	Time	H.less.Rate	0.148515	0.145425
58	Income	H.less.Rate	0.336634	0.100703
***59***	***HUDFunding***	***H.less.Rate***	***0.019802***	***0.233332***
***60***	***Eviction***	***H.less.Rate***	***0.09901***	***0.158226***
***61***	***SBUs***	***H.less.Rate***	***0.009901***	***0.460739***
***62***	***OccRate***	***H.less.Rate***	***0.009901***	***0.429864***
***63***	***Rent***	***H.less.Rate***	***0.019802***	***0.340935***


$$\begin{array}{c} H_{\text{0}}:w_{ij}^{C}=w_{ij}^{L}, \end{array}$$

where $w_{ij}^{C}$ and $w_{ij}^{L}$ are the (true) edge-strengths joining nodes i and j on the conservative and liberal sides and the test statistic used here was $E\left(w_{ij}^{C},w_{ij}^{L}\right)=\left|w_{ij}^{C}-w_{ij}^{L}\right|.$Other hypotheses may be formed and tested, too. Since a network is fundamentally a collection of edges, all of them, deals with edges – one at a time, or many. Another hypothesis, the hypothesis of *invariant network structure* is stricter, claiming that *all* the edge strengths are statistically equal across the networks. The test statistic changes naturally, and we look at the maximum of the edge-weight differences and reject the null assumption in case this maximum is too extreme. A strategy is to first check the hypothesis of invariant network structure, in case it is rejected (indicating at least one edge strength varies across the networks), move on to test the hypothesis of invariant edge strength to work out which edges are substantially different, much akin to the global F-test and the separate t-tests, to borrow a regression analogy. In our case, the invariant network structure statistic is 0.4607, leading to a p-value of 0.009 which suggests the existence of at least one edge strength that is substantially different across the two political networks. The page “Edge-Master” on our Robustness Analysis file (Supplementary Material) shows the fuller results of all p-values across all possible edges. P-values less than 0.1 or 0.05 suggest substantially different edge-strengths across political divides at 10% or 5% level of significance.

Another kind of test may be thought about – one that lifts our focus from a narrow, local power-edge analysis to a larger collective plane. One that checks whether the *overall* (that is, the total level of) connectivity has remained more or less similar across the two networks. This is called the hypothesis of *invariant global strength.* Using similar notations and assuming there are p nodes, the null assumption here may be framed as


$$\begin{array}{c} H_{\text{0}}:\sum_{i=\text{1}}^{p}\sum_{j>i}\left|w_{ij}^{C}\right|=\sum_{i=\text{1}}^{p}\sum_{j>i}\left|w_{ij}^{L}\right| \end{array}$$

with the test statistic being $S\left(w_{ij}^{C},w_{ij}^{L}\right)=\left|\sum_{i=\text{1}}^{p}\sum_{j>i}(\left|w_{ij}^{C}\right|-\left|w_{ij}^{L}\right|\right|$, and the p-values found through measuring the proportion of times – as discussed before through the other tests – the “null” values exceed the observed value of the statistic. This global invariant statistic, for this case is 0.7675, leading to a p-value of 0.386 which suggests the total amount of partial correlations (positive and negative) flowing in the Republican and Democrat networks are statistically similar.

***Technical note on multiple comparisons.*** Table 5 (appendix) reports permutation p-values for each pairwise edge comparison. Because 21 edges are tested simultaneously, we additionally report Benjamini-Hochberg false-discovery-rate-adjusted p-values and Holm-adjusted p-values alongside the raw values. Under cluster (state-level) permutation with unregularised precision matrices, edges may not survive either FDR or Holm correction at alpha = 0.05, which reflects the limited power available from only 50 state-level clusters relative to the number of edges tested, rather than an absence of political-stratum differences. We therefore treat the edge-level political differences reported here as exploratory rather than conclusively confirmatory, and rely on the joint cluster-permutation test (reported above) for the primary statistical inference about political-stratum differences.

### Assumptions and robustness

The Markov random fields described above estimate a conditional-independence structure rather than a causal one. To read an edge as a direct dependency that is not mediated by another measured variable, four assumptions must hold: (i) causal sufficiency (every common cause of any pair of measured variables is itself in the network), (ii) faithfulness (no exact cancellation of dependencies), (iii) correct distributional specification (Gaussianity of the variables), and (iv) acyclicity (no directed feedback loops). For state-level housing data the first is the most demanding — time-invariant state characteristics such as geography, climate, zoning history, labor-market structure, demographic composition, and political-cultural inheritance are plausible common causes that the pooled specification omits.

### Conceptual limits of the estimated networks

We restress that MRF edges do not distinguish confounders, mediators, colliders, and policy responses; nor do they license mechanistic causal interpretation. Some of the plausible causal structures underlying our variables would generate exactly the patterns we observe under different readings. Eviction, for instance, likely mediates part of the rent-homelessness relationship, so a network in which the direct Rent-Homelessness edge is weak (as in our FE-residualised specification, Fig 4) is compatible either with a genuinely small direct effect or with mediation through Eviction absorbing the association. HUD funding, similarly, is plausibly a policy response to prior homelessness levels rather than an exogenous cause of them; the lag-1 directed network in Fig 5 provides suggestive evidence in this direction, with both HUD - > Homelessness and Homelessness - > HUD coefficients estimable and non-trivial in Republican-led state-years. Formal disambiguation of these roles would require a directed acyclic graph framework with either domain-informed edge orientation or targeted instrumental variables. We flag this as important future work. In the present analysis, we restress, each network edge should be read as a conditional dependency structure worth investigating further, not as a causal claim.

We report four robustness specifications. First, we resample states (rather than state-years) under a cluster bootstrap with B = 100 replicates per stratum; this respects the within-state dependence noted in Temporal structure above and produces wider but better-calibrated confidence bands. Edges with bootstrap stability ≥ 0.70 across replicates are flagged as robust [74].

Second, we re-estimate the network on residuals from a regression of each variable on a full set of state dummies and a full set of year dummies. Edges describe within-state, year-adjusted partial associations. Of the 25 edges in the pooled raw-data network, 13 survive FE residualisation; the surviving edges are reported as our headline result. The asymmetry across strata is striking: 72% of edges in the Republican network do not survive FE residualisation (25 down to 7), while only 50% drop in the Democratic network (24 down to 12). The apparent richness of the pooled Republican network is therefore identified largely by between-state level differences rather than within-state covariation.

Third, we apply the nonparanormal transformation of Liu, Lafferty and Wasserman [75] (a rank-based normal-scores transform implemented via huge::huge.npn) to relax the Gaussianity assumption. This barely changes the edge structure — mild non-Gaussianity is not what is driving the network.

Fourth, we apply Fast Causal Inference [76] on the FE-residualised data via pcalg::fci with Fisher’s Z test at α = 0.05. FCI explicitly admits the possibility of latent confounders and returns a Partial Ancestral Graph in which bidirected edges flag pairs that are likely confounded by an unmeasured common cause. Across all three strata, FCI returns 6–7 edges and zero bidirected edges. This does NOT prove causal sufficiency holds (FCI is conservative on bidirected marks), but it does mean we find no positive evidence of remaining latent confounding once state and year fixed effects are partialled out. We report this as an assumption-supportive but not assumption-confirming finding.

Per-capita rescaling of HUD funding (described in Data preprocessing above) materially changes the headline HUD-Homelessness edge. With raw HUD dollars, the partial correlation is approximately zero (consistent with HUD allocations responding to, but being dominated by, state-size effects); with per-capita HUD funding, the partial correlation becomes +0.13 in the FE-residualised pooled network, and the HUD-Occupancy-rate edge changes sign. The per-capita specification is the headline result. The political asymmetry in the HUD-Homelessness edge magnitudes (Tables 4 and 6) is preserved under per-capita rescaling, FE residualisation, and the nonparanormal transformation.

**Table 6:** Partial correlations from Democrat-run states.

Baseline EBICglasso (raw) – edges = 24, lambda = 0.0086								
	Time	Income	HUDFunding	Eviction	SingleRoom	OccRate	Rent	HomelessnessRate
**Time**	0	0.0767	*−0.0542*	*−0.2202*	0.3576	*−0.1005*	0	*−0.4611*
**Income**	0.0767	0	*−0.316*	0.032	0	0.2787	0.7442	*−0.3051*
**HUDFunding**	*−0.0542*	*−0.316*	0	0	0.2434	0.4198	0.1562	*−0.1211*
**Eviction**	*−0.2202*	0.032	0	0	*−0.1281*	0	0.2086	*−0.1506*
**SingleRoom**	0.3576	0	0.2434	*−0.1281*	0	*−0.0566*	0.4722	0.6503
**OccRate**	*−0.1005*	0.2787	0.4198	0	*−0.0566*	0	*−0.0623*	0.0456
**Rent**	0	0.7442	0.1562	0.2086	0.4722	*−0.0623*	0	0.1262
**HomelessnessRate**	*−0.4611*	*−0.3051*	*−0.1211*	*−0.1506*	0.6503	0.0456	0.1262	0

#### Lag-1 directed temporal network and feedback cycles.

The cross-sectional MRF identifies conditional-independence structure but cannot, on its own, distinguish bidirectional feedback from unidirectional dependence. As an exploratory extension we estimate a lag-1 directed temporal network (Fig 5): for each target variable Y, Y at year t is regressed on lag-1 of all seven analytic variables, with state fixed effects (within-state demeaning) and BIC-tuned LASSO. The output is an asymmetric 7 × 7 coefficient matrix B in which B_ij is the lag-1 effect of variable i on variable j. A feedback cycle is a pair (i, j) with both B_ij and B_ji non-zero. Sample size: n = 349 state-year-pairs (Republican subsample n = 219, Democratic n = 130).

The directed networks differ sharply by political environment. The Republican lag-1 network contains 9 feedback cycles; the Democratic contains 4. The HUD-funding ↔ Homelessness-rate feedback loop — in which more HUD funding (per capita) in year t-1 predicts a higher homelessness rate in year t (β = +0.16), and a higher homelessness rate in year t-1 predicts lower HUD funding in year t (β = −0.11) — appears only in Republican-led states. In Democratic-led states, both lag effects are estimated at zero. A cluster-permutation test in which governor-party labels are reassigned at the state level (each state’s full time series receives a single label per replicate, B = 200 replicates) yields a one-sided p-value of 0.12. Sample sizes are small; we report the asymmetry as substantively suggestive and exploratory rather than as a confirmatory finding.

A note on the exchangeability of the political-stratum permutation test is in order. States that change party during the study window are assigned their modal party over the period, so each state's time series enters exactly one stratum. The cluster permutation preserves the within-state dependence structure that would be violated by naive edge-level permutation, at the cost of being conservative when only a subset of states switch. A complementary specification would be a unified panel model with party interactions, state and year fixed effects, and state-clustered standard errors; we did not pursue this in the present paper because the FE-residualised networks in Fig 4 already show how state and year fixed effects shape the conditional-dependence structure, and because such a model would test a different (parametric) null. The suggestive cluster-permutation p-value of 0.12 for the joint political difference is consistent with limited power from the 50 available state-level clusters.

Six additional politically-asymmetric findings emerge from the same lag-1 specification (Fig 6). (i) Income → Rent passthrough is 2.7 × stronger in Republican states (lag-1 β = +0.40 vs + 0.15 Democratic). (ii) Rent persistence is 1.6 × higher in Democratic states (autoregressive β = +0.70 vs + 0.45). (iii) Homelessness Rate persistence is 2.8 × higher in Democratic states (β = +0.53 vs + 0.19); Democratic-state homelessness has chronic-population character, Republican-state has transient-shock character. (iv) Eviction → Homelessness Rate next year is + 0.07 in Republican states; absent in Democratic states. (v) Occupancy → Rent next year is −0.23 in Republican states; absent in Democratic states. (vi) Republican states show five more total feedback cycles than Democratic. We report these as targets for future panel-VAR work on richer state-level data.

## Discussion

This study contributes to the existing literature arguing that the issue of homelessness be measured by expanding its framework beyond the contexts of the service system. We are in agreement with research that shows the issue of homelessness as a byproduct of extreme poverty [8,9,11]. Homelessness should be recognized as collateral of the larger issue of extreme poverty, and as such include structural indicators to explain the issue. The research we have reviewed previously shows that structural indicators bear more of a consequence than personal attributes in predicting homelessness, and that deinstitutionalization is inadequate in fully explaining contemporary homelessness [21,77]. Studies sampling from services are limited in their explanations of the issue to encompass only those who seek out such services [77].

Upon initial analysis, we discovered that as time increases since 2007, overall, homeless rates have decreased (see Note 7 in Appendix), along with evictions and occupancy rates. Although we show that homelessness has decreases overall from 2007–2024 [3], the increase in homelessness is substantial at 30% from 2020–2024 [3]. Over time, income also increased (Fig 3), but we want to emphasize that this does *not* control for inflation. Furthermore, rent, income, and evictions all impact each other positively (Fig 3). For example, the higher the income, rent also increases, and the cost of SBUs also increases. We see that as rent increases, evictions increase, and homelessness rates increase. Income has a slight inverse relationship with evictions, but this relationship is dwarfed by the hidden variable of rent, which shows a strong partial association between evictions and homelessness rates. We feel that this reflects higher instances of inadequacy to pay rent over evictions. We feel that this means people are being priced out of their homes over time.

Our findings reinforce Byrne et al. [30] cost-of-living index and Glynn et al. [33] in showing that rent and income are the strongest causal impact. This also aligns with Quigley and Raphael’s [21] findings that a 10% increase in rents results in a 6.5% increase in homeless rates and reducing rent-to-income ratios from 17.5% to 16.8% could result in a 25% decline in homelessness while focusing on vacancy rates only effected homelessness around 1%.

Income also plays the second largest role recognizing that rent is beyond what people can afford accounting for the existence of the working poor, which we feel reinforces the importance of considering the working homeless [5], and the important role of their insufficient income to pay rent. Glynn et al.’s [33] findings that homeless rates increase after median rental costs increase beyond 30% of median income also legitimize our findings and should be considered among policy intervention.

These findings collectively show that policymakers should prioritize addressing the cost of rent, specifically, and that focusing on other variables, such as increasing available units to impact housing markets long-term, may be less direct and distract from effective solutions. We are cautious about prescribing specific rent-policy instruments: the empirical literature on rent control reports mixed effects on tenants, landlords, and housing supply [78]. Our findings are consistent with the broad direction that policies addressing rent affordability merit attention, but our cross-sectional partial correlations do not by themselves identify which specific rent-policy instrument (rent control, voucher expansion, supply subsidy) would be most effective.

Rent stands out more among republican states (Fig F1 (Appendix F)), making it more of a straightforward causal indicator, whereas democrat states are more complex. The two variables with the highest impact among democrat states are the cost of SBUs, followed by income, then rent (Fig F1 (Appendix F)). We show that high rent followed by low income are the main predictors of homelessness, along with the cost of SBUs (Fig 4) among blue states. Among republican states, rent is more of a predictor of homelessness than the availability of SBUs (Fig F2 (Appendix F)). These variables also collectively have a strong relationship when we included the construction of new units in Fig F1 (Appendix F).

We feel that many of the differences between republican and democrat states can be attributed to larger populations residing in democrat states. For example, occupancy rates have an inverse relationship with time in both democrat and republican states. Democrat states show that as occupancy rates increase, so do homelessness rates, which we feel means that the larger populations have a more difficult time keeping up with the demand of the housing market. However, in republican states, this also has an inverse relationship with homelessness rates, which we feel means that rural areas could better focus efforts on lowing rent to reduce their homelessness rates over time. We see that when income increases, such as in republican states, then the cost of SBU dwellings increases as occupancy rates also decline (Fig 4).

Our findings on SBUs is reinforced by Rossi’s [12] explanation that many cities deliberately reduced their SBU stock throughout the 1990s to drive away poor populations via zoning changes, housing code enforcement, and urban renewal projects. Landlords have also converted their SBUs into multiroom units to increase rent. Our findings reinforce the seminal work of Rossi’s [12] historical analysis, advocating for the increased availability of increased SBUs across the U.S. to remedy widespread homelessness. We also interpret these findings to play a role in the geographic spaces that people live among cities in the U.S. Considering the persistence of how racially segregated areas in the U.S. are [79], focusing on reduced rent could improve issues of social inequality across racial lines, as well.

By comparing these variables to other popular structural explanations for homelessness, we show that occupancy rates, eviction rates, and the construction of new homes have less of an impact than rent, income, and SBUs (Fig D2 (Appendix D)). We feel this can inform policy debate moving forward.

### Social policy and landlord responses

Bradford and Bradford [80] tested the relationships between housing policies, evictions, and rent at the county-level across the U.S. finding the most common housing policies that impact likelihood of eviction is “disavowing eviction processed through small claims courts, prohibiting landlord retaliation, and requiring some wait time for eviction filing” (p24). These preventative measures can significantly reduce evictions. However, we feel that landlords can still raise the rent when the leases expire as a more direct (and easier) option because they do not need to go through official eviction processes. Since most tenants’ leases are likely to be only a year or less, they simply let the lease expire, then increase rent with the same tenant or a new one. If the tenant cannot pay the increased rent then they are forced to move rather than being evicted.

Research also shows that when policies restrict landlords, units can be removed from the market. Pastor and Abood [81] say:

Evidence suggests there is little negative impact on new construction, which is logical given that newly produced units have no initial rent targets. However, there is also research that suggests that rent regulations may lead landlords to reduce maintenance or take units off the market through conversion into condos or owner move-in evictions. Some proponents of rent regulations have argued for limiting what they see as loopholes by, for example, making condo conversion more difficult. (p4)

While there are efforts to mitigate changes in the housing stock, it should also be noted that the entire homeless population in the U.S., 771,480 [10] is less than the amount of vacant units in *one* the following states, for example: Florida, California or Texas [60]. The excessive vacant units in the U.S. should be considered when policies focus on adjusting for housing stock, which we discuss further for future research below.

The construction of new homes increases rent, which we find interesting because the increased housing stock should bring rent down. These findings also disagree with some existing research [82]. We believe that new construction increasing rent shows how new construction is not going toward low-income housing (as is intended in the planning phase of many projects) but instead resulting in new units going to higher rent-paying tenants. New construction’s impact on the homeless rate is not recognizable as a factor at all in Fig E2 (Appendix E). Given the inclusion of the other variables in this study, it also has no bearing on SBU dwellings. We interpret this to mean that although many projects start with the intention of implementing low-income housing (i.e., lower rent), the result is that they are renting as more expensive units (mainly because they are new) and have more bedrooms, which explains why people option to share units with roommates or live in units with more bedrooms than they actually need [43–45]. Similarly, rent has a direct relationship with income, which we interpret to reinforce that new construction is happening in states with higher income (see Note 8 in Appendix). This could be explained in many ways, considering *how* the construction of new units are implemented (governmental assistance or not), resulting in their utilization by higher income tenants.

Conversely, we show that investing in HUD results positively in both red and blue states, supporting the continuance of HUD funding despite efforts to significantly reduce it (Department of Housing and Urban Development [83]. Yet, our findings cannot support efforts to increase the housing stock, alone.

We also see that the more HUD funding is granted nationally to each state, their homeless rate decreases (Fig F1 (Appendix F) and 9)—that is, there is an inverse relationship between these variables. HUD funding decreased among republican states over time (Fig 4). HUD funding does not cause income to increase, but when increased occupancy rates increase, income follows. As states receive more HUD funding, the occupancy rate also increases. We can also see that the more income in a particular state, the less HUD funding is provided. We speculate that this could mean that HUD funding prevents occupancy rates from falling (because people are placed into housing via HUD), thusly housing stability provides a strong conditional association on income increasing. Knowing that homeless rates decline with the more HUD funding that is granted to each state legitimizes the continued funding of HUD and its programs, nationally. We feel this legitimizes the efforts of social service workers along with the programs that employ them.

These findings are reinforced by Desmond [84] who says, “Public initiatives that provide low-income families with decent housing they can afford are among the most meaningful and effective anti-poverty programs in America” (p302).

Additionally, the U.S department of treasury says

Since 2000, housing costs have been rising faster than median household income. Inflation-adjusted rents have grown steadily, to more than 20 percent above their 2000 level. Inflation-adjusted prices for single-family homes have grown even faster [49].

Bearing this in mind, we should be aware that rising cost of housing makes us all have increased risk of homelessness and tax dollars should be invested widely to mitigate the problem.

We also want to emphasize that just because our findings point to a few variables that have the most impact, other variables in our dataset should be given attention because they too can impact the issue of homelessness. Neglecting any other variables in the dataset could also increase homelessness in the long-term.

We feel that future research should explore the relationship between increased taxes, increased utilities that landlords may pay, or other factors that can help explain the high cost of rent. While these indicators have increased in many areas across the U.S., we feel a dearth of explanations for why rent has doubled or more in many areas compared to taxes points more to the opportunistic discretion of landlords.

### Limitations

Debate persists in finding an accurate national number of those who experience homelessness in the U.S., resulting in a total population that continues to go undercounted. In their annual analysis, Housing and Urban Development (HUD) estimates the total number of those experiencing homelessness in the United States to be 771,480 in 2024 [3], but many criticisms persist regarding the legitimacy of their numbers despite efforts for improvement [17,85]. For instance, many people go uncounted, yet still others are wrongfully assumed to be experiencing homelessness just because they appear disheveled [17,86]. The number of those experiencing homeless is widely understood to be undercounted, but the degree to which they are undercounted is unclear [87].

A further scope limitation is that the dependent variable throughout is the HUD Point-in-Time (PIT) homelessness count, which by design excludes many doubled-up, temporarily housed, and otherwise hidden populations. Our findings therefore describe the structural correlates of PIT-measured homelessness rather than homelessness in some broader or vague sense. Complementary measures such as McKinney-Vento student homelessness counts or ACS doubled-up estimates would be a natural extension (or bagged averages, thereof). We note the systematic comparison of structural correlates across measurement definitions, would be, on its own, a promising direction for follow-on work. As Smith notes [87],

*Readers may only consider, for example, those who sleep in public parks or are visibly congregating around services when speaking about the general homeless population. Others may limit their consideration to populations that stay at shelters or seek services, which neglects populations who do not seek such assistance. (p54)*

For this reason, discrepancies exist when we consider homelessness rates, as these rates are likely underestimates. Additionally, we show homeless rates decreasing over time in Fig 3. This is due to our expanded measurement from 2007 to 2024, which dilutes the increased homelessness due to economic downturns since 2020 (see Note 9 in Appendix). Despite their limitations, however, HUD’s Point-in-time Counts are considered to be the most accurate data at our disposal, so any critiques on this should account for all research that uses HUD data, including Congressional policy work for the Annual Homeless Assessment Report (AHAR) (see Note 10 in Appendix). We also only used data that are available and comparable, meaning that we do not include all years because some data were unavailable.

Despite the presence of numerous other funding sources at state and local government levels, as well as private funding sources, the majority of national funding for homelessness *housing* and shelter initiatives comes from HUD. We chose to use HUD for its broad impact, but future research should consider a more detailed analysis of measuring the impact of funding more expansively.

When considering the limited housing stock, it should be noted that there is an impending population cliff. It’s one thing to assert that there is a limited housing stock, but it needs to be acknowledged that the population will fall substantially in the coming decades at the current rate.

Five additional methodological limitations warrant explicit note. (i) Spatial scale: the analysis is at the state level, while housing-market dynamics operate at sub-state spatial scales (city, metro, neighborhood). Replication on Continuum-of-Care or census-tract data is a priority for future work, and our state-level partial associations cannot be attributed to any particular metro within a state. (ii) Ecological inference: state-level partial associations cannot be attributed to individuals or households. The classic ecological fallacy applies. (iii) Governor party as proxy: governor party is a coarse summary of the policy environment; legislative control, local zoning, state-level eviction protections, and the set of states in each stratum at any one moment all matter independently. (iv) Temporal scope of the eviction series: the Eviction Lab series is unavailable from 2019 onward at the time of writing, restricting the headline seven-variable panel to 2011–2018. A six-variable specification dropping eviction extends to 2011–2024 and produces qualitatively similar results. (v) Causal interpretation: the four assumptions described in Assumptions and Robustness (causal sufficiency, faithfulness, distributional specification, acyclicity) cannot all be verified from observational state-level data alone. We have probed each via a supplementary specification, but a graphical model on observational panel data is at most a hypothesis-generating tool for causal mechanisms, not a confirmatory one.

### Future research

The interpretations of the links between variables in Figs 2 and 5 (i.e. the link between homeless rates and rent) from one variable to another in a line play an important role in understanding the narrative of what is happening, and we invite other researchers to study how these relationships impact each other in more detail, as shown in our review of the literature. We offer an overall framework to observe a narrative between variables. However, future researchers should consider further explanation of the specific relationships among the variables.

We would also like to consider the current tax treatment of rental units that sit vacant. Our own calculations are as follows. Vacant units can write off $19,800–$26,300 (see Note 11 in Appendix) per year, per unit on average from taxpayers, totaling $54-$70 Billion annually in 2023 and 2024 (see Note 12 in Appendix in S1 Appendix) [88,89]. When compared to the entire annual HUD budgets in recent decades, which average only around $39.6B/yr from 2000–2009, $53.7B/yr from 2010–2019, and $72.4B/yr from 2020–2024 [90] we feel this is an understudied comparison, and future research should prioritize studying this phenomenon more. We feel that policymakers should focus on limiting the amount that landlords earn from allowing their units to be vacant over the construction of new homes and as a means to promote access to people who need to utilize these units.

We also feel that there should be more of a focus on why landlords increase rent, specifically. Is it taxes, utility bills, or something else?

Finally, additional variables should be considered in the analyses, such as with the inclusion of TANF, SNAP, and EITC at the state level. Researchers should compare these indicators and how they relate to what we have available in our dashboard. While we provide findings and discuss national trends here, we invite each state to run their own analyses from our online dashboard to understand their own structural trends over time.

## Supporting information

S1 File Robustness Analyses. (XLSX)

S1 Appendix Appendices_A_through_H and NOTES [91–115]. (DOCX)
