# Supplementary material for: A structural analysis of U.S. homelessness through Markov random fields: Identifying predictors and potential causal links through permutation tests
Source: PLoS One. 2026 Nov 4;21(11):e0343842. doi: 10.1371/journal.pone.0343842 (PMC13734467; doi:10.1371/journal.pone.0343842)
Supplement: Supplementary file 1 [file pone.0343842.s002.docx]

**Appendices A–H**

*Supplementary material to “Markov Random Fields for State-Level Homelessness Analysis”*

# Appendix A. Data Pipeline and Variable Construction

This appendix documents end-to-end how the analytic panel is constructed from raw public sources.

## A.1 Sources

All raw inputs are publicly archived. Annual files are named h{YYYY}.xlsx and are organized state-by-row, variable-by-column.

| **Variable** | **Source** | **Years** | **Construction** |
| --- | --- | --- | --- |
| Median household income | ACS 1-yr (Census 2025a) | 2011–2024 | Direct |
| HUD funding (per capita) | HUD Exchange (2025) | 2011–2024 | HUD$ / Population |
| Eviction rate | Eviction Lab (Desmond 2024) | 2011–2018 | Direct (state-year) |
| Single-Bedroom unit cost | ACS 1-yr (Census 2025a) | 2011–2024 | Direct (proxy for SRO) |
| Occupancy rate | ACS | 2011–2024 | Occupied / Total housing units |
| Median rent | ACS 1-yr (5-yr in 2020) | 2011–2024 | Direct |
| Population | ACS 1-yr (5-yr in 2020) | 2011–2024 | Direct |
| Homelessness rate | HUD PIT (2025) | 2011–2024 | PIT count / Population |
| New construction | Zillow (2025) | 2018–present | Aggregate of monthly state sales |
| Governor party | Kaggle US Governor (Conrady 2025) | All | Validated against NGA (2025) |

## A.2 Per-capita rescaling (NEW in revision)

HUD funding is now reported per capita (HUD dollars / state population). Raw HUD dollars correlate with state population at r = 0.94, so the original specification is dominated by state-size confounding. The per-capita rescaling changes the partial-correlation entries involving HUD substantially — in particular, the HUD–Homelessness Rate edge changes from approximately zero to a small positive partial association, and the HUD–Occupancy Rate edge flips sign. We propagate per-capita HUD through every specification (baseline, FE-residualised, nonparanormal, FCI, NCT).

## A.3 Missing-data treatment

Two structural missingness patterns affect the analysis:

- Eviction rate is unavailable from 2019 onward in the Eviction Lab archive at the time of writing. Listwise deletion on the seven-variable network therefore restricts the headline panel to 2011–2018 (n = 399 complete state-years from 50 states).
- New construction (Zillow) is available 2018-present. We use this variable only in the New-Construction subset analysis (8 variables, 2018, 2019, 2021, 2022; 2020 excluded due to ACS irregularity).

As sensitivities (see Appendix E): (i) a six-variable specification dropping Eviction extends the panel to 2011–2024; (ii) multiple imputation by chained equations on the eviction series extends the seven-variable specification through 2024.

## A.4 Standardisation and scale

After per-capita rescaling, all variables are standardised to mean 0 and variance 1 prior to estimation. Partial correlations are reported on the standardised scale. The standardisation matters: HUD per-capita (~$50/person) and Median Rent (~$1000/month) operate on radically different numerical scales.

# Appendix B. Temporal-Structure Diagnostics

This appendix reports lag-1 within-state autocorrelation, ADF stationarity, and within-vs-between variance decomposition.

## B.1 Within-state lag-1 autocorrelation and ADF stationarity

| **Variable** | **Mean ρ₁** | **Median ρ₁** | **% stationary (ADF)** | **Diagnosis** |
| --- | --- | --- | --- | --- |
| Income | +0.96 | +0.98 | 0% | Highly persistent / non-stationary |
| HUD Funding (per cap) | +0.08 | +0.06 | 2% | Volatile (allocation cycles) |
| Eviction Rate | +0.67 | +0.74 | 2% | Persistent, non-stationary |
| Single-Bedroom | +0.94 | +0.98 | 4% | Highly persistent |
| Occupancy Rate | +0.40 | +0.53 | 4% | Moderately persistent |
| Rent | +0.95 | +0.97 | 0% | Highly persistent |
| Homelessness Rate | +0.62 | +0.78 | 28% | Persistent in most states |

**Table B1.** *Within-state temporal-structure diagnostics computed across all 50 states. ρ₁ is the lag-1 autocorrelation. ADF column reports the percentage of state series for which the null of unit root is rejected at p < 0.05.*

Implication. Six of seven variables are highly persistent (lag-1 ρ ≥ 0.4 in median state) and almost no series is stationary at conventional levels. Treating state-years as exchangeable observations — the implicit assumption of the ordinary bootstrap and of the pooled GGM — will overstate the effective sample size. This motivates the cluster bootstrap (Appendix D), the FE-residualised primary specification (Appendix D), and the first-difference sensitivity (Appendix C).

## B.2 Variance decomposition: between vs within state

| **Variable** | **Between R²** | **Within** | **Implication** |
| --- | --- | --- | --- |
| Income | 0.844 | 0.156 | Mostly between-state |
| HUD Funding (per cap) | 0.981 | 0.019 | Almost entirely between-state |
| Eviction Rate | 0.993 | 0.007 | Almost entirely between-state |
| Single-Bedroom | 0.886 | 0.114 | Mostly between-state |
| Occupancy Rate | 0.953 | 0.047 | Almost entirely between-state |
| Rent | 0.904 | 0.096 | Mostly between-state |
| Homelessness Rate | 0.920 | 0.080 | Mostly between-state |

**Table B2.** *ANOVA-style variance decomposition. R² from regression of each variable on a full set of state dummies measures the fraction of total variance attributable to between-state differences.*

This is the most consequential diagnostic for the entire analysis. Eighty-four to ninety-nine percent of variation in every analytic variable is between states — i.e., reflects level differences across states rather than year-to-year movement within states. The pooled descriptive network is therefore overwhelmingly identified by between-state heterogeneity. The FE-residualised primary specification (Appendix D) sweeps this between-state component out and recovers within-state, year-adjusted partial associations.

# Appendix C. First-Difference and Lag-1 Sensitivity

Beyond the FE-residualised specification (Appendix D), we report two additional time-aware specifications

## C.1 First-difference specification

Each variable is replaced by its within-state first difference ΔX_{s,t} = X_{s,t} − X_{s,t-1} before standardisation and EBICglasso fitting. This eliminates persistent state-level levels at the cost of one year of data per state.

| **Specification** | **Pooled edges** | **Republican** | **Democratic** |
| --- | --- | --- | --- |
| Pooled (raw, baseline) | 25 | 25 | 24 |
| FE-residualised | 13 | 7 | 12 |
| First-difference | 9 | 6 | 8 |

**Table C1.** *Edge counts. First-differencing produces sparser networks than FE residualisation but yields qualitatively similar dependency patterns: the surviving edges are a subset of the FE-survivors. Reduced edge counts reflect both the harder identification (within-state changes only) and the smaller effective n.*

## C.2 Lag-1 directed network

As a final time-aware sensitivity, we estimate a lag-1 directed network: for each target Y, regress Y_t on lag-1 of all seven variables, with state fixed effects (within-state demeaning) and BIC-tuned LASSO. Results form the centrepiece of Appendix G below; here we note only that this specification provides explicitly directed (rather than undirected) inferences and is the natural complement to the contemporaneous GGM.

# Appendix D. Robustness Tables and Cluster-Bootstrap Stability

Note: in the revised numbering, the centrality bar-chart of the pooled MRF (formerly Fig 3 of the main paper) is referenced as Figure D2 in the body text, and the bootstrap-interval forest plot between variable pairs (formerly Fig 4) as Figure D3. Both of these figures elaborate the bootstrap-stability material described above; the underlying numbers are reported in Tables D1-D8.

The four robustness specifications consolidated. Detailed partial-correlation matrices, with edges color-coded by sign, are in the companion spreadsheet RobustnessAnalyses.xlsx (sheets Pol_Networks, Rep_Networks, Dem_Networks, Edge_Master).

## D.1 Edge counts

| **Stratum** | **Baseline** | **+ NPN** | **+ State × Year FE** | **+ FE & NPN** | **FCI (PAG)** |
| --- | --- | --- | --- | --- | --- |
| Pooled (n=399) | 25 | 27 | 13 | 14 | 7 |
| Republican (n=245) | 25 | 26 | 7 | 13 | 6 |
| Democratic (n=147) | 24 | 24 | 12 | 14 | 7 |

**Table D1.** *Edge counts under each robustness specification (yellow column = headline FE-residualised primary). Note the asymmetric drop in the Republican stratum (25 → 7 = 72% loss) versus the more modest drops in the Pooled and Democratic strata.*

## D.2 Cluster-bootstrap stability

The 50 states are resampled with replacement and the EBICglasso refitted; B = 100 replicates per stratum. Edge stability is the proportion of replicates in which an edge appears. Stability ≥ 0.70 is treated as robust evidence (Epskamp et al. 2018). Per-edge stability matrices are tabulated in RobustnessAnalyses.xlsx (sheet Bootstrap_Stability).

## D.3 FCI (Fast Causal Inference) on FE-residualised data

FCI returns a Partial Ancestral Graph (PAG) in which edge marks distinguish definite causal direction (‘->’) from latent-confounder-implicated bidirected edges (‘<->’), partially oriented edges (‘o->’), and uninformative edges (‘o-o’). Across all three strata, FCI returns 6–7 edges and zero bidirected edges. The interpretation: after state and year fixed effects are partialled out, the data do not positively imply remaining latent confounders among the seven measured variables. This does NOT prove causal sufficiency holds (FCI is conservative on bidirected marks), but it does mean the FE specification appears to capture the dominant latent structure. PAG edge listings are tabulated in RobustnessAnalyses.xlsx (sheet FCI_PAG).

# Appendix E. Imputation and Six-Variable Sensitivity

Note: the body text references Figure E2 (the eight-variable MRF with New Construction included, formerly Fig 5 of the main paper) and Figure E3 (the centrality reading of the same network, formerly Fig 6) for the New Construction sensitivity analysis. Note that Figure E1 of the previous numbering is now Figure 4 of the main paper.

Two robustness checks address the eviction-data hole from 2019 onward

## E.1 Six-variable specification (drop Eviction; 2011–2024)

We re-fit the EBICglasso on the six-variable panel (Time, Income, HUDFunding-per-capita, SingleRoom, OccRate, Rent, Homelessness Rate; Eviction excluded), extending the panel to 2011–2024. Sample size grows to n ≈ 700 complete state-years. Results: edges among the six retained variables are stable across the two specifications — of the 12 edges in the six-variable pooled network, 11 are present in the seven-variable headline; the lone difference is a marginal Income–OccRate edge that appears only in the seven-variable specification.

## E.2 Multiple imputation on Eviction (2019–2024)

As an alternative to dropping Eviction, we impute the Eviction-rate values for 2019–2024 via multiple imputation by chained equations (mice in R; m = 20 imputations; conditional models include Income, Rent, HUD per-capita, year, and state dummies). Pooled estimates of the EBICglasso edges are obtained by averaging the partial-correlation matrices across the m imputed datasets. Results are qualitatively identical to the headline 2011–2018 listwise specification, with edge magnitudes attenuated by ~15% on average (consistent with imputation noise).

# Appendix F. Political-Stratification Robustness

Note: the body text references Figure F1 (centrality across political parties, formerly Fig 8 of the main paper) and Figure F2 (edge-strength bootstrap intervals across the political split, formerly Fig 9). These are the political-stratification readings of the same data shown in Figure 4 of the main paper.

## F.1 State trifecta (governor + state house + state senate)

We replace governor-party with state-trifecta status: states classified as Republican-trifecta (governor + both legislative chambers Republican), Democratic-trifecta (all three Democratic), or split. The trifecta classification is sourced from Ballotpedia state-party-control records and applied year by year. Trifecta states comprise a smaller subsample (Republican trifecta: ~22 states across the panel; Democratic trifecta: ~13), so power is reduced. NCT on the trifecta-stratified networks: invariant network structure p = 0.04 (still rejected at 5%), invariant global strength p = 0.31 (not rejected), HUD–Homelessness per-edge p = 0.06 (marginal). Qualitatively, the political asymmetry survives the stricter classification.

## F.2 Continuous policy index

As a final check, we replace the binary party variable with a continuous “housing-policy progressivity index” constructed from three indicators — (i) presence of statewide just-cause-eviction protection, (ii) statewide rent-control authority, (iii) supplementary state housing-assistance programs — sourced from the Urban Institute’s state housing policy database. Edge magnitudes between Rent–Homelessness and HUD–Homelessness shift continuously with this index, consistent with the binary-party stratification finding.

# Appendix G. Lag-1 Directed Temporal Network and Feedback Cycles

Note: in the revised manuscript, the headline lag-1 directed networks (Figures 5 and 6) and the corresponding heatmap of Republican-versus-Democratic differences appear in the main paper. This appendix retains the methodological details, diagnostics, and cycle-substructure analysis (Figure G3, the cycle decomposition) that were not promoted to the main paper.

This appendix reports a lag-1 panel-VAR-style analysis that directly tests the cyclical-feedback hypothesis raised in our Limitations section: that *Rent → Homelessness → Policy → Rent* may be a closed feedback loop on annual data, and that the loop may differ by political environment. The analysis goes beyond the contemporaneous GGM by using temporal ordering to identify directed lag effects.

## G.1 Specification

For each target Y ∈ {7 network variables}, we estimate

*Y_{s,t} − Ȳ_s = α + Σ_v β_{v→Y} (X_{s,t-1}^v − Ȳ_s^v) + ε_{s,t}*

where the within-state demeaning absorbs state fixed effects and β_{v→Y} are estimated jointly by LASSO with BIC tuning (Liu, Lafferty & Wasserman 2009 nonparanormal; Friedman et al. 2008 graphical lasso adapted to univariate-target case). All variables are standardised post-demeaning so coefficients are comparable in magnitude. The output is an asymmetric 7×7 coefficient matrix B in which B_{ij} is the lag-1 effect of variable i on variable j. A

**feedback cycle** is a pair (i, j) with both B_{ij} ≠ 0 and B_{ji} ≠ 0 — i.e., bidirectional lag-1 dependence.

Sample size: n = 349 state-year-pairs (a state-year observation is included if both year t and year t−1 are complete-case). Republican subsample: n = 219; Democratic subsample: n = 130.

## G.2 Cycle counts and the HUD–Homelessness asymmetry

| **Stratum** | **n** | **# non-zero lag edges** | **# feedback cycles** | **HUD↔Homeless cycle?** |
| --- | --- | --- | --- | --- |
| Pooled | 349 | 23 | 7 | Weak (β = +0.11, −0.13) |
| Republican | 219 | 27 | 9 | YES (β = +0.16, −0.11) |
| Democratic | 130 | 21 | 4 | ABSENT (β = 0, 0) |

**Table G1.** *Cycle structure by political stratum. The HUD↔Homelessness feedback loop — in which more HUD this year predicts more homelessness next year (β = +0.16) AND more homelessness this year predicts less HUD next year (β = −0.11) — appears only in Republican-led states.*

## G.3 The political asymmetry: HUD ↔ Homelessness loop

In Republican-led states, the lag-1 effect of HUD funding (per capita) on the homelessness rate is +0.159, and the lag-1 effect of homelessness rate on HUD funding is −0.105. Both are non-zero under BIC-tuned LASSO, forming a closed feedback loop. In Democratic-led states, both lag effects are estimated at zero — the feedback loop is empirically absent.

Several mutually-non-exclusive interpretations are consistent with this pattern:

- (i) HUD allocations to Republican-led states are responsive to need with a one-year lag (the +0.16 HUD → Homelessness next year is consistent with allocations targeting persistent rather than transient need; the −0.11 Homelessness → HUD next year is the harder direction — it would suggest HUD allocations DECREASE in years following high-homelessness years, which would be unusual unless administrative discretion at the state level is differently exercised by Republican administrations).
- (ii) Democratic-led states have larger and more bureaucratically-stable HUD allocation processes that do not respond to short-term homelessness fluctuations; the lag-1 link is broken because the allocation logic operates on longer or more institutional timescales.
- (iii) Selection: the set of states that elect Republican governors differs from the set that elect Democratic governors on dimensions (urbanisation, homeless-population composition, federalism dynamics) that themselves drive the differential lag structure, rather than party causing the structure.

We do not attempt to adjudicate among these. The robust empirical claim is that the HUD↔Homelessness feedback loop is a Republican-state-only phenomenon at the lag-1 within-state level.

## G.4 Cluster-permutation test for the asymmetry

To assess significance we perform a cluster-permutation test in which the governor-party labels are permuted at the state level (each state’s entire time series is reassigned a single label per replicate). The test statistic is the Republican-minus-Democratic difference in summed |HUD↔Homelessness lag coefficients|. Observed value: 0.264. Under B = 200 cluster permutations, the one-sided p-value is 0.12 (two-sided p = 0.21). Sample sizes are small; the asymmetry is suggestive rather than conclusive at conventional significance thresholds. We report the finding as exploratory and direct readers to the figure-level evidence (Figure G3) and the cycle counts (Table G1) as the primary qualitative basis.

## G.5 Other politically-asymmetric findings

Six additional asymmetries are visible in the lag-1 networks (Figures 5 and 6 (main paper)):

- Income → Rent passthrough is 2.7× stronger in Republican states (lag-1 β = +0.40 vs +0.15 Democratic). Republican-state income shocks transmit to rents within one year; Democratic-state passthrough is slower.
- Rent persistence is 1.6× higher in Democratic states (autoregressive β = +0.70 Dem vs +0.45 Rep). Democratic-state rents are stickier.
- Homelessness Rate persistence is 2.8× higher in Democratic states (autoregressive β = +0.53 Dem vs +0.19 Rep). Democratic-state homelessness has chronic-population character; Republican-state homelessness has transient-shock character.
- Eviction → Homelessness Rate next year is +0.07 in Republican states; absent in Democratic states. Within Republican states, evictions feed into homelessness with a one-year lag; in Democratic states the link is empirically broken at lag-1, possibly via tenant protections or longer eviction-to-displacement timelines.
- OccRate → Rent next year is −0.23 in Republican states; absent in Democratic states. Tighter housing markets in Republican states are followed by lower rents next year (regression-to-mean / out-migration / new construction supply response); no such effect in Democratic states.
- Republican network has 9 feedback cycles; Democratic has 4. Republican states’ within-state housing-economy dynamics are more interconnected at the lag-1 level.

## G.6 Caveat on causal interpretation

Lag-1 within-state directed effects are stronger evidence of causal direction than contemporaneous undirected partial correlations, but they are not definitive. They are consistent with directed mechanisms but also with omitted dynamic factors that drive both variables with different lag structures. We report Appendix G as exploratory and substantively suggestive, complementing the static GGM analysis but not replacing it.

# Appendix H.

Note: In Ference on edge strengths with p-values are derived from permutation tests (FDR controlled).

| **Edge** | **rho_Rep** | **rho_Dem** | **diff** | **p_raw** | **p_BH** | **p_Holm** |
| --- | --- | --- | --- | --- | --- | --- |
| SBU - Homeless | 0.015 | 0.517 | -0.502 | 0.072 | 0.686 | 1 |
| Income - SBU | 0.315 | -0.159 | 0.474 | 0.122 | 0.686 | 1 |
| SBU - Rent | 0.296 | 0.614 | -0.319 | 0.148 | 0.686 | 1 |
| Eviction - Rent | 0.488 | 0.279 | 0.21 | 0.174 | 0.686 | 1 |
| Income - Homeless | -0.132 | -0.407 | 0.275 | 0.186 | 0.686 | 1 |
| Occ - Homeless | -0.202 | 0.133 | -0.334 | 0.196 | 0.686 | 1 |
| Income - Rent | 0.613 | 0.79 | -0.177 | 0.296 | 0.888 | 1 |
| Eviction - Homeless | -0.257 | -0.063 | -0.193 | 0.408 | 0.988 | 1 |
| Income - Occ | 0.449 | 0.226 | 0.223 | 0.488 | 0.988 | 1 |
| Rent - Homeless | 0.337 | 0.166 | 0.171 | 0.512 | 0.988 | 1 |
| SBU - Occ | -0.191 | 0.012 | -0.203 | 0.542 | 0.988 | 1 |
| Income - HUD | 0.206 | 0.101 | 0.105 | 0.6 | 0.988 | 1 |
| Eviction - SBU | -0.176 | -0.281 | 0.105 | 0.71 | 0.988 | 1 |
| HUD - Rent | -0.139 | -0.046 | -0.093 | 0.756 | 0.988 | 1 |
| Occ - Rent | -0.169 | -0.062 | -0.107 | 0.774 | 0.988 | 1 |
| HUD - Eviction | 0.049 | 0.002 | 0.047 | 0.808 | 0.988 | 1 |
| HUD - Occ | -0.204 | -0.136 | -0.068 | 0.84 | 0.988 | 1 |
| HUD - Homeless | 0.177 | 0.136 | 0.041 | 0.86 | 0.988 | 1 |
| Income - Eviction | -0.159 | -0.054 | -0.105 | 0.902 | 0.988 | 1 |
| Eviction - Occ | 0.047 | 0.031 | 0.016 | 0.972 | 0.988 | 1 |
| HUD - SBU | 0.075 | 0.079 | -0.004 | 0.988 | 0.988 | 1 |

**Notes**

**Note 1.**

HUD has not released the 2025 homeless Point-in-Time (PIT) count numbers as of the writing of this paper. They still need to tally everything after each of the Continuums of Care (CoCs) submit their data through the Homelessness Data Exchange (HDX) 2.0. The deadline for CoCs to submit their data was June 13, 2025, at 8:00 PM EDT, but HUD has yet to post.

**Note 2.**

We are aware that Census data is available from other sources for pre-2010—For example please see [60]. However, we thought it was more desirable to pull from only a single data source rather than account for multiple sources for the same variables to preserve the uniformity of our study.

**Note 3.**

Data on SROs is difficult to attain because there is no federal registry for such efforts, only city-by-city and advocacy group initiatives. They can be impossible to enumerate given that hotels/motels often serve as pop-up SROs in temporary situations following natural disasters or when local shelters overflow, then they return to their typical function as being rented out nightly to paying customers. SROs also decreased drastically from the 1950s to the 1980s [42]. For this reason, we utilize Single-Bedroom Units (SBUs) rather than a shared housing unit among roommates. We also think this portrays an expanded purview of impact on the rental market than SROs, especially given that people who historically inhabited SROs are now placed in SBUs via a litany of voucher programs [62,63].

**Note 4.**

Although the Census website suggests using five-year estimates for more accuracy, single year is desirable here to compare across years.

**Note 5.**

Please see methods section for our reasoning to utilize single bedroom units (SBUs) instead of SROs.

**Note 6.**

Readers interested in mathematical technicalities behind the reliability of these networks (such as whether true edges are missed or spurious edges are shown with statistical regularizers such as the Lasso) are pointed to a series of works spanning diverse application fields: Bhaduri [57] and Zahirodini and Bhaduri [58] for business applications, Bhaduri [77] for legal, Bhaduri and Predescu [78] and Bhaduri [79] for health, Camp et al. [80] for pedagogical, Bhaduri [81,82], Bhaduri et al. [83] for sports, Setiawan and Bhaduri [84] for finance, Bhaduri [85], Bhaduri [86–88], Bhaduri and Ho [89], Ho and Bhaduri [90], Ho and Bhaduri [91] for weather science. Success in such varied fields bolsters confidence in the techniques. These applied works are inspired by the theoretical developments shown in Bhaduri [92], Ho et al. [93], Zhan et al. [94], Bhaduri and Zhan [95], Bhaduri et al. [96,97].

**Note 7.**

Our dataset measures trends from 2007 to 2024 and cannot account for year-to-year fluctuations, such as years following 2020.

**Note 8.**

These new construction data were from all states but spanned only 4 years, due to limitations of data.

**Note 9.**

During the Great Recession, policies such as the American Recovery and Reinvestment Act of 2009 implemented Homelessness Prevention and Rapid Re-housing Program funds which continue to keep people on the verge of homelessness housed via “rapid-rehousing” efforts [112].

**Note 10.**

Housing and Urban Development reports to U.S. Congress in an Annual Homeless Assessment Report (AHAR) providing nationwide estimates of homelessness to inform new federal policy on homelessness.

**Note 11.**

When a dwelling qualifies as a residential rental property, regardless if it is vacant, the owner is entitled to claim deductions for all typical and essential expenditures. These include, but are not limited to, interest on mortgages, taxes levied on the property, insurance premiums, costs of utilities, expenses for upkeep, as well as depreciation among other costs [113,114].

**Note 12.**

This estimate is our own calculation. Put simply, U.S. Census Bureau, “National Vacancy Statistics,” Housing and Vacancy Characteristics Survey, 2022 [61]. The report provides comprehensive data on housing unit status, including the following statistics: units for rent (3.1 million), units rented but not occupied (0.51 million), units for sale only (1.0 million), units sold but not occupied (0.36 million), units used seasonally such as vacation homes (4.4 million), migrant worker housing (4,000), and other vacant units (4.8 million), amounting to a total of 14.2 million vacant units. The IRS says that would total around $54-$70 Billion annually in 2023 and 2024 [113,114].
