# Supplementary material for: A structural analysis of U.S. homelessness through Markov random fields: Identifying predictors and potential causal links through permutation tests
Source: PLoS One. 2026 Nov 4;21(11):e0343842. doi: 10.1371/journal.pone.0343842 (PMC13734467; doi:10.1371/journal.pone.0343842)
Supplement: Supplementary file 2 [file pone.0343842.s004.docx]

**Response to Editor**

Dear Editor,

Thanks for your efficient handling of our article. Below, you will find responses to each point raised by the reviewers. Here, in this section, we address your observations specifically.

Sincerely,

Curtis Smith and Moinak Bhaduri

1. Please ensure that your manuscript meets PLOS ONE's style requirements, including those for file naming. The PLOS ONE style templates can be found at

https://journals.plos.org/plosone/s/file?id=wjVg/PLOSOne_formatting_sample_main_body.pdf and <https://journals.plos.org/plosone/s/file?id=ba62/PLOSOne_formatting_sample_title_authors_affiliations.pdf>

**Response:** Thanks. This is checked.

2. We have noted that you have mentioned on Pg 11 of your manuscript, that ' Python scripts facilitated the process (please see Appendices A through C)', however it appears that these Appendices have not been uploaded as part of your submission. Therefore, kindly upload these appendices as part of your revised manuscript.

**Response:** The article has been reworked and this issue doesn’t apply anymore. The Appendices have been placed now as figshare files (below, as requested). We are uploading these Appendices on the online submission portal since they contain references to the issues raised by the three reviewers who may find these responses and changes useful, but these need not be added to the main manuscript (the mathematical technicalities could distract from the main theme) and can be accessed from figshare instead.

3. Please update your submission to use the PLOS LaTeX template. The template and more information on our requirements for LaTeX submissions can be found at http://journals.plos.org/plosone/s/latex.

**Response:** We noted on <https://journals.plos.org/plosone/s/submission-guidelines> that Plos One allows Word submissions. This is why the typesetting, formatting, was done on Word. If instead, Latex is insisted on, could we please format it post-acceptance, since this requires substantial editing and probably would put us past the 5/25 deadline.

4. We note that the grant information you provided in the ‘Funding Information’ and ‘Financial Disclosure’ sections do not match.

**Response:** Here is our funding statement from the article. We will ensure things are consistent as we resubmit.

MB received funding from the American Mathematical Society and the Simons Foundation. CS and MB received funding from Bentley University.

5 and 6: … on Funding…

**Response:** We are paying attention to those now.

7. Review your statements relating to the author contributions, and ensure you have specifically and accurately indicated the role(s) that these authors had in your study. These amendments should be made in the online form.

**Response:** We are paying attention to those now.

8. Confirm in your cover letter that you agree with the following statement, and we will change the online submission form on your behalf:

**Response:** This could be our statement:

“The funders provided financial and logistical support but did not have any additional role in the study design, data collection and analysis, decision to publish, or preparation of the manuscript. The specific roles of these authors are articulated in the ‘author contributions’ section.”

9. Thank you for uploading your study's underlying data set. Unfortunately, the repository you have noted in your Data Availability statement does not qualify as an acceptable data repository according to PLOS's standards. At this time, please upload the minimal data set necessary to replicate your study's findings to a stable, public repository (such as figshare or Dryad) and provide us with the relevant URLs, DOIs, or accession numbers that may be used to access these data. For a list of recommended repositories and additional information on PLOS standards for data deposition, please see <https://journals.plos.org/plosone/s/recommended-repositories>.

**Response:** Thanks, these have been posted now as figgshare files. Following is an excerpt:

Data, Appendices, and Robustness Analyses have been placed in: https://doi.org/10.6084/m9.figshare.32340900

An interactive dashboard: https://moinak.shinyapps.io/HousingMRFShinyDashboardMoinak/ lets users vary the free parameters in the underlying regularisers and note the impacts.

10. Please ensure that you refer to Figure 6 in your text as, if accepted, production will need this reference to link the reader to the figure.

**Response:** Again, the figures have been renumbered this round and this issue doesn’t apply anymore.

**Response to Reviewers**

Dear Editor and Reviewers,

We are grateful for the thorough and constructive feedback. The reviewers raise serious methodological points, and addressing them has materially improved the paper. In keeping with Plos One’s interdisciplinary theme and wide readership, we refrained from including them in the first round. We are glad to observe there is interest in these mathematical technicalities and have refined the article accordingly (for instance, we appreciate how “causality” can be a trigger word in certain settings even in the light of anecdotal and quantitative evidence. These terms have been softened, and sections have been added that outline assumptions and show how they have been met). Below we respond to each concern in order, indicate the changes made, and point to the specific manuscript sections, the supplementary R interactive dashboard/code, and the new appendices where each change is reflected. **Some notes before we begin:**

- We have added a complete R interactive dashboard and reproducibility script (https://moinak.shinyapps.io/HousingMRFShinyDashboardMoinak/ and HousingMRF.R, supplied as Supplementary Material) that regenerates every figure, table, p-value, and bootstrap interval reported in the paper from the publicly archived state-level data (the data have been placed now on figshare). This addresses Reviewer 1, point 3.vii directly.
- Several reviewer concerns are interrelated (e.g., causal sufficiency, ecological inference, state-size heterogeneity). We have grouped the response so that each concern receives a substantive answer in one place; cross-references avoid duplicate replies.
- One reviewer-driven analytical change has substantively shifted a headline result and we want to flag it here: when HUD funding is correctly rescaled to per-capita to address Reviewer 2 (point 3) and Reviewer 3 (point 4), the partial correlation between HUD funding and the homelessness rate changes from a near-zero edge to a small POSITIVE one, and the HUD–Occupancy-Rate partial correlation flips sign. The original raw-HUD specification was substantially confounded by state size (HUD raw vs Population r = +0.94). The revised manuscript reports the per-capita specification as primary and is more cautious in claims about HUD funding’s relationship with homelessness.
- In addition to the reviewer-driven changes, we have added an exploratory lag-1 directed temporal-network analysis (new Section in the Discussion; full results in S7 Appendix). This addresses the cyclical-feedback question that emerges naturally from our acyclicity caveat — whether Rent → Homelessness → Policy → Rent operates as a closed loop on annual data — and reveals a politically asymmetric pattern: a closed HUD↔Homelessness feedback loop is detectable in Republican-led states but not in Democratic-led states. We frame this analysis as exploratory (cluster-permutation p = 0.12) rather than confirmatory and treat it as a hypothesis-generating extension.

**Reviewer 1**

**Comment 1.1 — Causal inference and assumptions**

*“The methodology employed (MRFs based on partial correlations) does not, on its own, justify causal inference without strong assumptions such as causal sufficiency, absence of latent confounders, and appropriate model specification. These assumptions are neither stated nor justified.”*

**Response.** We agree, and have addressed this comment substantively. The revised manuscript:

- Replaces causal phrasing with dependency phrasing throughout the Findings section. Specifically, the sentence “these Markov random fields may be taken as tools representing causal (as opposed to correlational) connections” has been removed, and references to “causal impact-generating potentials” have been replaced with “node centrality” / “expected influence in the dependency network.”
- Adds a new subsection, “Assumptions and Robustness”, that explicitly states and discusses the four assumptions a causal reading would require — causal sufficiency, faithfulness, correct distributional specification, and acyclicity — and acknowledges that time-invariant state characteristics violate causal sufficiency in the pooled specification.
- Reports four supplementary analyses that empirically probe these assumptions: (i) a cluster bootstrap of edge stability that resamples states (rather than state-years) and so respects within-state dependence; (ii) re-estimation on residuals from a two-way state-and-year fixed-effects model, which removes time-invariant state-level and common annual confounding; (iii) a nonparanormal transformation that relaxes Gaussianity; and (iv) the FCI algorithm of Spirtes, Meek and Richardson (1999), which explicitly admits latent confounders and returns a Partial Ancestral Graph. Headline finding: the nonparanormal transformation barely changes the network, but two-way fixed-effects residualisation removes ~48% of the edges in the pooled network and ~72% of the edges in the Republican network. The edges that survive across all four robustness checks constitute the most defensible structural features and are now reported as the headline result.

*See revised Methods (Section* ***Estimation and Implementation****), the new* ***Assumptions and Robustness*** *subsection, Appendix D (Robustness Tables), and HousingMRF.R §§6–9.*

**Comment 1.2 — Temporal structure**

*“The analysis does not adequately account for the temporal structure of the data … there is no discussion of temporal dependence, autocorrelation, or non-stationarity, which raises concerns about the validity of the results.”*

**Response.** This is a fair concern, and we now address it both diagnostically and in the model specification. We have run within-state lag-1 autocorrelation diagnostics and Augmented Dickey-Fuller (ADF) tests on every variable; the results are summarised in Table T1 below and are reported in full in Appendix B.

| **Variable** | **Mean lag-1 ρ** | **Median lag-1 ρ** | **% stationary (ADF, p<.05)** | **Diagnosis** |
| --- | --- | --- | --- | --- |
| Income | +0.96 | +0.98 | 0% | Highly persistent / non-stationary |
| HUD Funding | +0.08 | +0.06 | 2% | Year-to-year volatile (allocations vary) |
| Eviction Rate | +0.67 | +0.74 | 2% | Persistent, non-stationary |
| Single-Bedroom | +0.94 | +0.98 | 4% | Highly persistent / non-stationary |
| Occupancy Rate | +0.40 | +0.53 | 4% | Moderately persistent |
| Rent | +0.95 | +0.97 | 0% | Highly persistent / non-stationary |
| Homelessness Rate | +0.62 | +0.78 | 28% | Persistent in most states |

*Table T1. Within-state lag-1 autocorrelation and ADF stationarity tests.*

The diagnostics confirm that several variables (Income, Rent, SBU costs) are highly persistent within states (lag-1 ρ ≈ 0.95), and almost no series is stationary. We therefore make two changes in the revised manuscript:

- We re-estimate the network on residuals from a two-way fixed-effects model that includes state effects and year effects. The year effects absorb common annual shocks and the linear trend, and the state effects absorb persistent state-level levels. Edges from this specification reflect within-state, year-adjusted covariation — the appropriate target when temporal persistence is this strong.
- We acknowledge that an explicit time-series model (vector autoregression, dynamic graphical model, or graphical-lasso on first differences) would be a still cleaner solution. We report a first-difference specification as a sensitivity check in Appendix C; results are qualitatively consistent with the FE-residualised specification but lose statistical power because differencing absorbs much of the level variation.
- We have added explicit text clarifying that “Time” in the original network represents a linear trend in calendar year (operationalised as Year minus 2010, ranging 1–14); under the FE specification the Time node is dropped because year fixed effects subsume it.

*See revised Methods (****Temporal structure*** *subsection), Appendix B (autocorrelation/ADF diagnostics), Appendix C (first-difference sensitivity), and HousingMRF.R §4.*

**Comment 1.3 — Reproducibility (i–vii)**

*“The methods are not described with sufficient detail to ensure reproducibility …”*

**Response.** We have substantially expanded the Methods to address each sub-point, and now provide the full R script as Supplementary Material. We respond to each sub-point in turn:

**(i) Exact estimation procedure.** The MRF is estimated as a Gaussian Graphical Model (GGM) via the graphical lasso of Friedman, Hastie and Tibshirani (2008), with the regularisation parameter selected by the Extended Bayesian Information Criterion (EBIC) of Foygel and Drton (2010). This is implemented in R via qgraph::EBICglasso called from bootnet::estimateNetwork(default = "EBICglasso"). The revised Methods explicitly says so.

**(ii) Tuning parameter selection.** EBIC selects λ from a 100-point logarithmic path between λ_max (the maximum off-diagonal absolute value of the empirical covariance) and 0.01 × λ_max. The EBIC tuning parameter γ is fixed at 0.5 (the bootnet default; values of γ ∈ {0, 0.25, 0.5} produce qualitatively identical networks, reported in Appendix D). The selected λ for each network is now reported in Table 2 of the revised manuscript.

**(iii) Standardisation.** All variables are standardised to zero mean and unit variance via scale() before estimation. Partial correlations are reported on the standardised scale. The revised Methods says so explicitly.

**(iv) Missing data.** Eviction-rate data from the Eviction Lab (Desmond, 2024) are unavailable for any state from 2019 onward at the time of writing; ACS one-year estimates were not produced for 2020 due to COVID-19, so for that one year only we use five-year estimates for housing-unit counts, median rent, and population (as documented in the Data Sources section). The revised manuscript now restricts the temporal scope of the headline analysis to 2011–2018 (where complete cases are available) and reports a sensitivity analysis on a 6-variable network (dropping Eviction) for 2011–2024 in Appendix E. Complete-case analysis is used; mean imputation and multiple imputation give qualitatively similar results (also reported in Appendix E).

**(v) Type of correlation.** Pearson partial correlations (the natural choice for a Gaussian Graphical Model) are used throughout. The revised Methods says so. The introductory example using student “hours studied” and “scores” had referred to a Spearman correlation — we have corrected the text to clarify that this was illustrative only and is not the procedure used in the main analysis.

**(vi) Permutation testing.** The Network Comparison Test (NCT) of van Borkulo et al. (2023) is used. The procedure is now described algorithmically in the Methods (“Permutation Tests for Network Equality” subsection): in each of B = 1{,}000 iterations, the political-party labels are randomly permuted across states, two networks are re-estimated, and the test statistics for invariant network structure (max edge difference), invariant edge strength (per-edge difference), and invariant global strength (sum of absolute edge weights) are computed; the empirical p-value is the proportion of iterations whose statistic equals or exceeds the observed statistic. We use B = 1{,}000 (rather than the bootnet default of 100) for the revised analysis. Implementation: NetworkComparisonTest::NCT() with it = 1000.

**(vii) Code.** The full R script HousingMRF.R is provided as Supplementary Material. It is organised into nine sections (data ingestion, harmonisation, scaling, baseline EBICglasso, NCT, cluster bootstrap, FE residualisation, nonparanormal sensitivity, FCI). The script is self-contained: it re-creates every figure, every table, and every p-value reported in the manuscript from the publicly archived input files. The dashboard URL we provided previously is retained as an exploration aid but is now explicitly labelled as supplementary, not as the primary reproducibility artefact.

*See revised Methods (****Estimation and Implementation*** *subsection), HousingMRF.R, and Appendices B–E.*

**Comment 1.4 — Conclusions**

*“(i) Causal claims should be revised. (ii) Policy recommendations such as rent control are not directly tested. (iii) The Republican–Democrat comparison may suffer from ecological interpretation issues.”*

**Response.**

- (i) See response to 1.1 above. All explicit causal claims (“cause,” “causal impact,” “causal flows”) have been replaced with dependency language (“conditional association,” “centrality,” “expected influence in the dependency network”).
- (ii) The reviewer is correct that we did not directly test rent control. We have removed the rent-control recommendation from the Conclusions and replaced it with a more cautious statement: “The consistent positive partial association between Rent and Homelessness Rate across specifications and political strata is consistent with rent-affordability mechanisms emphasised in the housing literature (Hanratty 2017; Glynn et al. 2021), but our design does not adjudicate among specific policy interventions.” Housing-supply policies are now treated symmetrically: noted as plausible, not endorsed.
- (iii) The ecological-interpretation concern is well-taken and is addressed via three changes. First, we now warn explicitly in the Findings that state-level partial associations cannot be attributed to individuals or households (the standard ecological-fallacy caveat). Second, we re-estimate the political-party comparison after FE residualisation, which removes within-party between-state heterogeneity — the differential edge between HUD funding and homelessness rate that we previously highlighted survives FE adjustment, but with attenuated magnitude. Third, we add a per-capita rescaling for HUD funding (see opening note of this letter and responses to R2#3, R3#4); this changes some headline edges and is now propagated through the political-party comparison.
- Additionally, we have added a paragraph noting that governor’s party is an imperfect proxy for the policy environment (legislative control, local zoning, state-level eviction protections all matter independently); see response to R3#7 below.

**Reviewer 2**

**Comment 2.1 — MRF nomenclature**

*“In a stricter mathematical sense, an MRF is more than just an undirected graph: it is usually defined through an underlying joint probability distribution together with Markov properties … I do not think it is sufficient to label the resulting graph as a MRF simply because it is undirected.”*

**Response.** This is a fair and important methodological point, and we have rewritten the Methods section in response. The model we estimate is in fact a special case of a Markov Random Field — specifically, a **Gaussian Graphical Model** (GGM), which is the Gaussian instance of an undirected probabilistic graphical model with the pairwise, local, and global Markov properties (Lauritzen 1996; Koller and Friedman 2009). Concretely, if X = (X₁, …, Xₚ) follows a multivariate normal distribution with concentration (precision) matrix K = Σ⁻¹, then *K_{ij} = 0 ⇔ X_i ⊥ X_j | X_{V\{i,j}}* — i.e., the pattern of zeros in the precision matrix encodes pairwise conditional independence, which under the Hammersley–Clifford theorem is equivalent (for strictly positive distributions) to the local and global Markov properties.

The revised Methods explicitly states (a) the joint Gaussian assumption, (b) the relationship between zeros in the precision matrix and the Markov properties, (c) that the graphical lasso is a sparsity-inducing estimator of the precision matrix, and (d) that the resulting graph encodes the conditional independence structure of the joint distribution. We have also acknowledged the reviewer’s implicit point that we do not perform a separate test of Markov-property violation; under the Gaussian assumption the property is automatic, but we now report a Mardia test of multivariate normality (rejected on the raw data, not rejected on the nonparanormal-transformed data — see Appendix D), which is consistent with using the nonparanormal-transformed network as a robustness specification.

*See revised Methods (****The Gaussian Graphical Model*** *subsection) and Appendix D.*

**Comment 2.2 — Definition of partial correlation**

*“The authors describe it as regressing two variables on the others and then taking ‘the ratio of the error variances,’ which I do not think is a standard or accurate way to define partial correlation …”*

**Response.** The reviewer is right — this was an error in our exposition. The correct definition is the **correlation of residuals**, not the ratio of variances. We have rewritten the relevant paragraph to give two equivalent definitions:

- Residual definition. Let r_i be the residual from regressing X_i on the conditioning set Z, and r_j the residual from regressing X_j on Z. Then the partial correlation ρ_{ij·Z} = corr(r_i, r_j).
- Inverse-covariance definition (Lauritzen 1996; Epskamp et al. 2018). If Σ is the covariance matrix of (X₁,…,Xₚ) and K = Σ⁻¹ is the precision matrix, then ρ_{ij·V\{i,j}} = −K_{ij} / √(K_{ii} K_{jj}). The graphical lasso estimates a sparse K, and the off-diagonal partial correlations are computed from this estimate.

The two definitions coincide for jointly Gaussian variables. We thank the reviewer for catching this; the revised Methods presents both definitions and explicitly cites Epskamp et al. (2018) and Lauritzen (1996) rather than Epskamp et al. (2012).

**Comment 2.3 — Data preprocessing (scaling, missing values)**

*“The variables are on quite different scales … unclear how this was handled in the regression-based partial correlation analysis. I also could not tell clearly how missing values or uneven data availability across variables and years were treated.”*

**Response.** We have added a new “Data Preprocessing” subsection to the Methods, which addresses each issue.

- Scaling. All seven (or eight, when Time is included) variables are standardised to zero mean and unit variance prior to estimation. This is now stated explicitly. Furthermore, in response to R3#4 below, we now use per-capita rescaling for HUD funding (HUD dollars / state population) before standardisation; the same approach is applied to homeless count (already a per-capita rate) and to housing-unit counts (rates per population, where applicable). This addresses the size confounding the reviewer flags.
- Missing values. Documented in Appendix A. The headline 2011–2018 specification uses listwise deletion (n=399 complete state-years from 50 states); a 6-variable specification dropping Eviction extends to 2011–2024; multiple-imputation results are reported in Appendix E and produce qualitatively identical networks.
- Variable harmonisation. The 2020 ACS irregularity is now described explicitly (5-year estimates substituted for that single year on housing-unit counts, median rent, and population).

*See revised Methods (****Data Preprocessing*** *subsection) and Appendix A.*

**Comment 2.4 — State comparability**

*“U.S. states differ a lot in population size, geographic scale, economic development … It is not clear to me whether the analysis accounts for this state-level heterogeneity in a way that makes the states meaningfully comparable.”*

**Response.** This is the most important methodological concern in the entire round of review, and our revisions reflect that. We have run a variance decomposition that quantifies how much of each variable’s variation is between states (i.e., a level difference) versus within states across time (Table T2).

| **Variable** | **Between-state R²** | **Within-state** | **Implication** |
| --- | --- | --- | --- |
| Income | 0.844 | 0.156 | Mostly between-state |
| HUD Funding | 0.981 | 0.019 | Almost entirely between-state |
| Eviction Rate | 0.993 | 0.007 | Almost entirely between-state |
| Single-Bedroom | 0.886 | 0.114 | Mostly between-state |
| Occupancy Rate | 0.953 | 0.047 | Almost entirely between-state |
| Rent | 0.904 | 0.096 | Mostly between-state |
| Homelessness Rate | 0.920 | 0.080 | Mostly between-state |

*Table T2. Variance decomposition of each network variable into between-state and within-state components.*

This is striking: 84–99% of the variation in every variable is between states. The pooled partial-correlation network was therefore predominantly identified by between-state heterogeneity. We have responded with two changes:

- Per-capita rescaling. HUD funding is now reported per capita (HUD dollars per state resident), removing the strongest source of size confounding. As reported in the opening note of this letter, this changes the headline HUD–Homelessness edge from a near-zero edge to a small positive one and flips the sign of the HUD–Occupancy edge.
- Two-way fixed-effects specification. We re-estimate the entire network on residuals from a regression on state and year dummies. This sweeps out ALL time-invariant state-level confounders (geography, climate, zoning history, labor markets, political culture). Edges that survive this specification reflect within-state covariation — the comparable quantity the reviewer is asking for. Roughly half of the edges in the pooled network and 72% of the edges in the Republican network do not survive, which is itself a substantively important and reportable finding. The revised manuscript leads with the FE-residualised network as the primary specification and treats the pooled network as descriptive context.

*For the political comparison specifically, the FE-residualised NCT is reported alongside the pooled NCT in Table 4 of the revised manuscript. The HUD–Homelessness edge difference between Republican and Democratic states attenuates but remains statistically significant in the FE specification.*

*See revised Methods (****Data Preprocessing, Estimation and Implementation****), the new* ***Assumptions and Robustness*** *subsection, and Appendix D.*

**Reviewer 3**

**Comment 3.1 — Framing relative to the literature**

*“The paper presents structural explanations as correcting a narrow focus on individual factors, but this is not new …”*

**Response.** Agreed. The Introduction has been rewritten to position our contribution as *adding a graphical-model perspective* to an existing multi-level structural literature, rather than correcting a deficit. Specifically, we now cite the multi-level frameworks of Lee, Tyler & Wright (2010), Shinn (2007), Burt (2001), and Culhane et al. (2007), and frame our contribution as: “applying conditional-independence network methods to a body of structural variables that the qualitative and panel-econometric literatures have already identified as important—our contribution is methodological, not substantive.”

**Comment 3.2 — PIT count: which population?**

*“The manuscript uses PIT counts but does not clearly state what population this represents … What type of homelessness is being measured …?”*

**Response.** We have added a new paragraph in the Data Sources subsection that explicitly defines the PIT count: it is HUD’s Point-in-Time count, conducted on a single night each January, of **both sheltered (in emergency shelters and transitional housing) and unsheltered (on the street, in vehicles, in places not meant for habitation) individuals** within each Continuum of Care, aggregated to the state level. We now state the well-documented limitations explicitly: PIT counts are widely understood to undercount the unsheltered population, are conducted in winter when the unsheltered population may be smaller, and do not capture “housed but housing-insecure” populations such as those doubled-up with family. We cite the National Alliance to End Homelessness (2020) and Hopper et al. (2008) for these limitations and note that the homelessness rate in our analysis should be read as a proxy for the population most visibly homeless on a single winter night, not for the full population at risk of homelessness.

**Comment 3.3 — Conceptual framework**

*“The variables are listed and justified individually, but there is no clear model explaining how they fit together …”*

**Response.** We have added a new “Conceptual Framework” subsection at the end of the Introduction that lays out the housing-affordability—housing-supply—safety-net triangle within which our variables operate:

- Housing affordability axis: Income, Rent, Eviction Rate, Single-Bedroom unit cost — measures the gap between what households can pay and what housing costs.
- Housing supply axis: Occupancy Rate, New Construction (where available) — measures whether the existing stock meets demand.
- Safety-net axis: HUD Funding (per capita) — measures the public-sector cushion that catches households who fall through.
- Outcome: Homelessness Rate — hypothesised to depend on all three axes.

This is presented as a heuristic to organise variable interpretation, not as a structural causal model. We make this distinction explicitly. A schematic of the framework is now included as Figure 1 (and the original Figure 1 demonstrating partial correlation has been moved to Figure 2).

**Comment 3.4 — State-level vs metro-level / Simpson’s paradox**

*“The analysis is conducted at the state level, while much of the cited literature focuses on metro-level housing markets … Could aggregation bias or Simpson’s paradox affect the results?”*

**Response.** The reviewer is correct that housing-market dynamics operate at sub-state spatial scales, and we have added explicit discussion in the Limitations section. Three points:

- State-level analysis is dictated by the temporal coverage and consistency of HUD PIT counts and ACS rent and income measures, all of which are reliably available at the state level back to 2011 but not at the Continuum-of-Care or county level for the full panel. We acknowledge this is a constraint, not a virtue.
- We agree that Simpson-style sign reversals are possible if metro-level dependencies differ systematically from state-level ones; the reviewer is correct that this is not testable within our data. We now state in the Limitations: “State-level associations may differ qualitatively from those at the metro or county level. Replication on Continuum-of-Care data, where housing-market and homelessness-count units more closely coincide, is an important next step.”
- The variance decomposition reported in response to R2#4 (Table T2 above) is itself a partial diagnostic: the dominance of between-state variance means that pooled partial correlations are largely identified by cross-state level differences, which the FE-residualised specification removes.

**Comment 3.5 — Time coverage, time as a node, panel structure**

*“How are variables harmonized across time? What does ‘time’ represent in the model? Why not use a panel structure with time fixed effects or lagged relationships?”*

**Response.** See response to R1#2 above for the temporal-structure response and R2#3 for harmonisation. On the specific point about ‘time as a node’: in the original network Time entered as a continuous variable equal to (Year − 2010), so its edges to other variables capture linear trends. In the revised manuscript:

- The original Time-as-node specification is retained as the descriptive baseline, but interpreted as a linear-trend proxy rather than as a substantive causal node.
- In the FE-residualised specification (the new primary), Time is dropped because year fixed effects subsume it; we explicitly note this in the Methods.
- Lagged relationships. We report a sensitivity check using lag-1 X variables predicting current homelessness rate (Appendix C). Results are qualitatively consistent but underpowered (n drops to ~350 after lagging).
- First-differencing as an alternative to FE: also reported in Appendix C; results consistent.

**Comment 3.6 — Causal interpretation**

See response to R1#1 and R1#4i; the entire treatment of causal language has been overhauled.

**Comment 3.7 — Governor party as proxy for policy environment**

*“Governor party alone may not capture the relevant policy environment …”*

**Response.** We agree. We have added a paragraph in the Limitations explaining that governor’s party is being used as a coarse summary of state-level political-policy orientation, that legislative control (state house, state senate, supermajority status), local zoning regimes, and state-level eviction protections all matter independently and are not captured by this single variable, and that the differential edges we report should be read as “associated with the broader political-policy context that produces a Republican vs Democratic governor” rather than “directly attributable to gubernatorial party.” As a sensitivity check, we report the political-comparison NCT with state-trifecta status (party controlling governor, state house, and state senate) substituted for governor-party in Appendix F. Results are qualitatively similar but with reduced statistical power because trifecta states are a smaller subsample.

**Comment 3.8 — Rent control claims**

See response to R1#4ii. Rent-control claims have been removed; housing-supply policies are now treated symmetrically as plausible-but-untested mechanisms.

**Additional exploratory analysis added in this revision**

Beyond the changes driven by individual reviewer comments, we have added an **exploratory lag-1 directed temporal-network analysis** that addresses two related concerns implicit in the reviews:

- Reviewer 1 (point 1) noted that an undirected MRF cannot identify causal direction, and our own discussion in the new Assumptions and Robustness subsection raised the possibility that variables such as Rent, Homelessness Rate, and HUD funding may be cyclically related (Rent → Homelessness → Policy → Rent) on annual data. The lag-1 panel-VAR-style analysis directly tests for such feedback cycles by looking for variable pairs (i, j) with both X_i,t-1 → X_j,t and X_j,t-1 → X_i,t non-zero coefficients.
- Reviewer 3 (points 5 and 7) asked whether different mechanisms might be operating in different political environments. The lag-1 analysis is run separately for Republican-led and Democratic-led state-years and reveals a striking and substantively interpretable asymmetry.

**Headline finding**

The HUD-funding ↔ Homelessness-rate feedback loop is a **Republican-state-only phenomenon** in our data. Within Republican-led states, more HUD funding (per capita) in year t-1 predicts a higher homelessness rate in year t (β = +0.16), AND a higher homelessness rate in year t-1 predicts lower HUD funding in year t (β = −0.11). Both directions are non-zero under BIC-tuned LASSO, forming a closed feedback loop. Within Democratic-led states, *both* lag effects are estimated at zero.

In total, the Republican lag-1 network contains **9 feedback cycles**; the Democratic network contains **4**. A cluster-permutation test of the HUD↔Homelessness asymmetry under random reassignment of party labels at the state level (B = 200) yields a one-sided p-value of 0.12. Sample sizes are small (n_R = 219 and n_D = 130 state-year-pairs after lagging); we therefore report the asymmetry as substantively suggestive and exploratory rather than as a confirmatory finding.

**Six additional politically-asymmetric findings**

- Income → Rent passthrough is 2.7× stronger in Republican states (lag-1 β = +0.40 vs +0.15 Democratic). Income shocks transmit to rents within one year in Republican-led states; transmission is slower in Democratic states.
- Rent persistence is 1.6× higher in Democratic states (autoregressive β = +0.70 vs +0.45). Democratic-state rents are stickier.
- Homelessness Rate persistence is 2.8× higher in Democratic states (β = +0.53 vs +0.19). Democratic-state homelessness has chronic-population character; Republican-state homelessness has transient-shock character.
- Eviction → Homelessness Rate next year is +0.07 in Republican states; absent (β ≈ 0) in Democratic states. In Republican states, evictions feed into homelessness with a one-year lag; in Democratic states the link is empirically broken at lag-1.
- OccRate → Rent next year is −0.23 in Republican states; absent in Democratic states. Tighter housing markets in Republican states are followed by lower rents next year (consistent with regression-to-mean, out-migration, or supply response); no such effect appears in Democratic states.
- Republican states’ within-state housing-economy dynamics are more interconnected at the lag-1 level (9 feedback cycles vs 4).

**Where this is reported in the manuscript**

- S7 Appendix presents the full specification, all coefficients, the cycle inventory, and the cluster-permutation test.
- Three new figures visualise the result: Fig 5 shows the side-by-side directed lag-1 networks, Fig 6 shows the Republican-minus-Democratic coefficient-difference heatmap, and S7 Appendix contains a four-node Rent–Homelessness–HUD–Eviction substructure plot showing the cycle asymmetry.
- HousingMRF.R has been extended (Section 14) to reproduce the analysis end to end, including the cluster-permutation test.
- The Discussion section of the main manuscript contains a dedicated subsection (“Republican-only HUD–Homelessness feedback cycle”) that summarises the finding and explicitly frames it as exploratory.

**Why we report this as exploratory**

Lag-1 within-state directed effects are stronger evidence of causal direction than contemporaneous undirected partial correlations — they exploit the temporal ordering, which a contemporaneous GGM does not — but they are not definitive. The cluster-permutation p-value of 0.12 means the observed asymmetry would arise about 12% of the time under random party assignment; this is consistent with a real underlying signal but does not clear conventional significance thresholds. We have therefore framed the analysis consistently as exploratory throughout the manuscript and supporting materials.

We report the analysis because it is responsive to the reviewers’ implicit and explicit interest in feedback structure and political mechanism, and because the qualitative pattern (a Republican-only HUD–Homelessness feedback loop) is interpretable and worth flagging for further investigation by the housing-policy community even if the formal test is suggestive rather than conclusive.

**Summary of changes to the manuscript**

- Methods section completely rewritten: now contains “The Gaussian Graphical Model,” “Data Preprocessing,” “Temporal Structure,” “Estimation and Implementation,” and “Permutation Tests for Network Equality” subsections (R1#3, R2#1, R2#2, R2#3, R3#5).
- Conceptual framework subsection added to Introduction (R3#3).
- Causal language replaced with dependency language throughout (R1#1, R1#4i, R3#6).
- Per-capita rescaling for HUD funding implemented; headline edges revised accordingly (R2#3, R2#4, R3#4).
- Two-way fixed-effects residualised network reported as primary specification (R1#2, R2#4, R3#4, R3#5).
- PIT-count definition and limitations made explicit (R3#2).
- Rent-control and housing-supply claims tightened to associations rather than recommendations (R1#4ii, R3#8).
- New “Assumptions and Robustness” subsection: cluster bootstrap, FE residualisation, nonparanormal, FCI (R1#1, R1#4iii, R2#1, R2#4, R3#4, R3#6).
- Limitations section expanded to address ecological inference, governor-party-as-proxy, state-vs-metro scale (R1#4iii, R3#4, R3#7).
- Six new appendices (S1–S6) plus a new S7 Appendix containing the exploratory lag-1 directed temporal-network analysis: A (Data Pipeline), B (Temporal Diagnostics), C (Lagged & First-Differenced Sensitivity), D (Robustness Tables), E (Imputation Sensitivity), F (Political-Stratification Robustness), G (Lag-1 Directed Temporal Network and Feedback Cycles).
- New exploratory section in the Discussion (“Republican-only HUD–Homelessness feedback cycle”) summarising the lag-1 finding and six additional politically-asymmetric findings, with explicit framing as exploratory rather than confirmatory.
- Full R reproducibility script (HousingMRF.R) supplied as Supplementary Material, including Section 14 that reproduces the new cycle analysis end to end (R1#3vii).

We are grateful to the reviewers for the depth and care of their comments. We believe the revised manuscript is substantially improved as a result. Sincerely, The Authors

**New references added in this revision**

- Borkulo, C. D. van, Boschloo, L., Borsboom, D., Penninx, B. W. J. H., Waldorp, L. J., & Schoevers, R. A. (2023). Comparing network structures on three aspects: A permutation test. Psychological Methods.
- Burt, M. R. (2001). Homeless families, singles, and others: Findings from the 1996 National Survey of Homeless Assistance Providers and Clients. Housing Policy Debate, 12(4), 737–780.
- Culhane, D. P., Metraux, S., Park, J. M., Schretzman, M., & Valente, J. (2007). Testing a typology of family homelessness based on patterns of public shelter utilization. Housing Policy Debate, 18(1), 1–28.
- Epskamp, S., Borsboom, D., & Fried, E. I. (2018). Estimating psychological networks and their accuracy: A tutorial paper. Behavior Research Methods, 50(1), 195–212.
- Foygel, R., & Drton, M. (2010). Extended Bayesian Information Criteria for Gaussian Graphical Models. NeurIPS 23.
- Friedman, J., Hastie, T., & Tibshirani, R. (2008). Sparse inverse covariance estimation with the graphical lasso. Biostatistics, 9(3), 432–441.
- Glynn, C., Byrne, T. H., & Culhane, D. P. (2021). Inflection points in community-level homeless rates. Annals of Applied Statistics, 15(2).
- Hanratty, M. (2017). Do local economic conditions affect homelessness? Impact of area housing market factors… Housing Policy Debate, 27(4), 640–655.
- Hopper, K., Shinn, M., Laska, E., Meisner, M., & Wanderling, J. (2008). Estimating numbers of unsheltered homeless people through plant-capture and postcount survey methods. American Journal of Public Health, 98(8), 1438–1442.
- Koller, D., & Friedman, N. (2009). Probabilistic Graphical Models: Principles and Techniques. MIT Press.
- Lauritzen, S. L. (1996). Graphical Models. Oxford University Press.
- Lee, B. A., Tyler, K. A., & Wright, J. D. (2010). The new homelessness revisited. Annual Review of Sociology, 36, 501–521.
- Liu, H., Lafferty, J., & Wasserman, L. (2009). The nonparanormal: Semiparametric estimation of high-dimensional undirected graphs. JMLR, 10, 2295–2328.
- National Alliance to End Homelessness. (2020). Methodology of the Annual Homeless Assessment Report.
- Shinn, M. (2007). International homelessness: Policy, socio-cultural, and individual perspectives. Journal of Social Issues, 63(3), 657–677.
- Spirtes, P., Meek, C., & Richardson, T. (1999). An algorithm for causal inference in the presence of latent variables and selection bias. In Computation, Causation, and Discovery (pp. 211–252). MIT Press.
