# Supplementary material for: A structural analysis of U.S. homelessness through Markov random fields: Identifying predictors and potential causal links through permutation tests
Source: PLoS One. 2026 Nov 4;21(11):e0343842. doi: 10.1371/journal.pone.0343842 (PMC13734467; doi:10.1371/journal.pone.0343842)
Supplement: Supplementary file 3 [file pone.0343842.s005.docx]

Response to reviewer 1 (second round)

Observation: The authors have addressed most of the concerns raised during the previous review. The manuscript is now considerably more transparent, reproducible, and statistically rigorous. The addition of robustness analyses, fixed-effects residualization, discussion of temporal dependence, and public availability of code and data substantially strengthens the work. The authors now correctly state in several locations that the estimated Markov Random Fields are descriptive conditional dependence networks.

Answer: Thanks for your encouragement and careful reads during the previous rounds.

Response to reviewer 2 (second round)

Observation: I thank the authors for addressing all of my concerns, which has improved the quality of the manuscript.

Answer: Thanks for your support and careful reads during the previous rounds.

Response to reviewer 3 (second round)

We are grateful to reviewer 3 for these observations. We agree these do bolster some of our findings’ messaging without altering their analytical core/essence. We detail below the changes we have made this round

Observation 1: On prior studies.

Answer: We have (i) removed the "far higher" phrasing and replaced it with a more moderate version pointing readers to the new Table 1a, and (ii) added Table 1a ("Comparison with prior state-level and community-level studies of homelessness") comparing the present work with Quigley & Raphael, Byrne et al., Heston, and Glynn et al. along the axes you’ve suggested. We have added

Several studies have examined structural predictors of homelessness at various levels of aggregation. Table 1a situates the present analysis alongside four of the most relevant. Our contribution is not primarily the breadth of covariates (Heston, for instance, considers 25 state-level variables, and Glynn et al. examine 381 Continuums of Care) but the use of conditional-dependence networks (Gaussian Graphical Models) to map the joint structure of the variables, the Network Comparison Test with cluster permutation to compare political strata, and the exploratory lag-1 directed specification to probe temporal ordering.

**Table 1a. Comparison with prior state-level and community-level studies of homelessness.**

| **Study** | **Spatial unit** | **Period** | **Method** | **Contribution** |
| --- | --- | --- | --- | --- |
| Quigley & Raphael (2001) [16] | 50 metros | 1990 | OLS regression | Housing-market conditions as primary drivers of MSA-level homelessness |
| Byrne et al. (2013) [24] | 381 CoCs | 2007-2010 | Multilevel regression | Community-level determinants; role of unemployment and rent |
| Heston (2019) [25] | 50 states | single cross-section | OLS with 25 predictors | Broad state-level covariate scan; correlation with PIT count |
| Glynn et al. (2021) [27] | 381 CoCs | 2007-2020 | Bayesian nonparametric regression | Homelessness, affordability, poverty relationship; CoC-level |
| This study | 50 states + DC | 2011-2024 (varies) | Gaussian Graphical Models; NCT; cluster permutation; lag-1 DAG | Conditional-dependence mapping; political stratification with panel-respecting inference |

Observation 2: On confounders, mediators, colliders, and policy responses…

Response: Under “Assumptions and Robustness”, we have added the following that’ll (i) acknowledge the ambiguity of edge interpretation when confounders, mediators, colliders, and policy responses coexist among the variables; (ii) discusses two of the most consequential cases that you are perhaps pointing to - Eviction as a plausible mediator between Rent and Homelessness, and HUD funding as a plausible policy response to prior homelessness levels; and (iii) note that the lag-1 directed network (Figs 5 and 6) provides suggestive evidence on the HUD-Homelessness feedback question, and that formal disambiguation would require a DAG framework with either domain-informed edge orientation or targeted instrumental variables.

***Conceptual limits of the estimated networks***. We restress that MRF edges do not distinguish confounders, mediators, colliders, and policy responses; nor do they license mechanistic causal interpretation. Some of the plausible causal structures underlying our variables would generate exactly the patterns we observe under different readings. Eviction, for instance, likely mediates part of the rent-homelessness relationship, so a network in which the direct Rent-Homelessness edge is weak (as in our FE-residualised specification, Fig 4) is compatible either with a genuinely small direct effect or with mediation through Eviction absorbing the association. HUD funding, similarly, is plausibly a policy response to prior homelessness levels rather than an exogenous cause of them; the lag-1 directed network in Fig 5 provides suggestive evidence in this direction, with both HUD -> Homelessness and Homelessness -> HUD coefficients estimable and non-trivial in Republican-led state-years. Formal disambiguation of these roles would require a directed acyclic graph framework with either domain-informed edge orientation or targeted instrumental variables. We flag this as important future work. In the present analysis, we restress, each network edge should be read as a conditional dependency structure worth investigating further, not as a causal claim.

Observation 3: On scope of outcome measure (PIT versus broader homelessness…).

Answer: Thanks. We’ve added the following under limitations:

A further scope limitation is that the dependent variable throughout is the HUD Point-in-Time (PIT) homelessness count, which by design excludes many doubled-up, temporarily housed, and otherwise hidden populations. Our findings therefore describe the structural correlates of PIT-measured homelessness rather than homelessness in some broader or vague sense. Complementary measures such as McKinney-Vento student homelessness counts or ACS doubled-up estimates would be a natural extension (or bagged averages, thereof). We note the systematic comparison of structural correlates across measurement definitions, would be, on its own, a promising direction for follow-on work.

Observation 4: On which sample is for which finding…

Answer: Thanks. Yes, with a large data set like this and several analyses, tracking which set serves to offer which result can get tedious. We have added a dedicated table (Table 1c, below), under Data Preprocessing. The Appendix E language regarding the New Construction analysis are updated The Zillow New Construction data are available for 2018 and 2019 only (a two-year window, not "2018-2023" or "four years" as previously stated), yielding n = 86 state-years across 43 states.

***Analytic samples.*** To avoid confusion between the several sub-samples used across the paper, Table 1c lists each network specification, its sample window, sample size (n = state-years), the number of unique states contributing, and the variables entering the specification. The abstract's 2007-2024 range refers to the outer bounds of data availability across all analyses; individual specifications use narrower windows determined by variable coverage. In particular, the seven-variable main network is restricted to 2011-2018 because state-level eviction rates are systematically recorded only through 2018 in the source database.

***Table 1c. Analytic samples across the paper.***

| **Analysis** | **Window** | **n** | **States** | **Variables** | **Reported in** |
| --- | --- | --- | --- | --- | --- |
| Pooled headline GGM | 2011-2018 | 399 | 50 | 7 (Income, HUD per cap, Eviction, SBU, Occ, Rent, Homeless) | Fig 3, Table 2 |
| FE-residualised, pooled | 2011-2018 | 399 | 50 | 7 (as above, residualised on state + year FE) | Fig 4A, Appendix D |
| FE-residualised, Republican-led | 2011-2018 | 245 | 38 | 7 (as above) | Fig 4B |
| FE-residualised, Democratic-led | 2011-2018 | 147 | 26 | 7 (as above) | Fig 4C |
| Lag-1 directed network | 2011-2018 | 348 pairs | 50 | 7 x 2 (contemporaneous + lag-1) | Fig 5, Fig 6, Appendix G |
| 6-variable extended (drop Eviction) | 2007-2024 | 458 | 51 | 6 (Income, HUD per cap, SBU, Occ, Rent, Homeless) | Appendix E |
| 8-variable with New Construction | 2018-2019 | 86 | 43 | 8 (headline 7 + NC; Eviction dropped for 2019 obs) | Appendix E |
| Republican vs Democratic NCT | 2011-2018 | 245 + 147 | 38 + 26 | 7 (headline set) | Table 4 |

Observation 5: Prediction-type language and regularizers…

Answer: We feel there are two things here. On the first, we agree that a GGM estimates conditional associations rather than "predictors" in, say, a machine-learning sense of out-of-sample performance sense. We have modified wording, replacing "predictors" with "conditional associations" or "conditional dependencies" where the earlier phrasing implied predictive performance. On the second point, i.e., whether main conclusions hold under an unregularized precision matrix, we appreciate your methodological instinct. We re-estimated the political-stratum comparison using unregularized precision matrices (see updated Table 4_withFDR). The edge-sign structure is materially unchanged from the EBIC-glasso version and the two specifications agree on the signs of all 21 edges and on the ranking of the largest partial correlations. The regularized specification is retained because it produces sparser (more interpretable) plots for Figs 3-4, but nothing in the substantive conclusions depends on regularization. We elected not to add a parallel unified panel FE regression with clustered standard errors because our FE-residualised networks (Fig 4) already show how the state and year fixed effects reshape the conditional-dependence structure. Also, because a unified panel model would test a different (parametric) null hypothesis than the exchangeability-based tests we reported.

Observation 6: Exchangeability of network comparison tests…

Answer: Yes, you are right that the NCT was developed for two independent cross-sectional datasets, and that states switching party during the study window introduce a within-cluster time-varying label that could violate the assumption of one label per unit. Our response has been to use a state-level cluster permutation in which each state's time series is assigned a single (modal) party label, so each state contributes to exactly one stratum. This is a different null hypothesis from the naive edge-level permutation. It tests whether the two joint edge-strength distributions differ across state-level political groupings, respecting the within-state dependence, and we have added a Discussion paragraph in blue below, making this explicit. We chose cluster permutation because it preserves the panel structure of the data at the cost of being conservative when only a subset of states switch (this is an unavoidable tradeoff at 50 clusters). We agree that a unified panel model with party interactions, state and year fixed effects, and state-clustered standard errors would provide a valuable complementary test; we flag this as a natural next step and note that our FE-residualised network already isolates the within-state, year-adjusted component of each partial correlation.

A note on the exchangeability of the political-stratum permutation test is in order. States that change party during the study window are assigned their modal party over the period, so each state's time series enters exactly one stratum. The cluster permutation preserves the within-state dependence structure that would be violated by naive edge-level permutation, at the cost of being conservative when only a subset of states switch. A complementary specification would be a unified panel model with party interactions, state and year fixed effects, and state-clustered standard errors; we did not pursue this in the present paper because the FE-residualised networks in Fig 4 already show how state and year fixed effects shape the conditional-dependence structure, and because such a model would test a different (parametric) null. The suggestive cluster-permutation p-value of 0.12 for the joint political difference is consistent with limited power from the 50 available state-level clusters.

Observation 7: On multiple comparisons…

Answer: We have added Benjamini-Hochberg (BH) FDR-adjusted and Holm-adjusted p-values as new columns in Table 4 alongside the raw permutation p-values (appendix). We report that under the cluster (state-level) permutation with unregularized precision matrices, individual edge do not survive either BH or Holm correction at alpha = 0.05. This reflects the power limitation of 50 state-level clusters relative to 21 edges being tested, rather than an absence of political-stratum differences. We have added a Multiple Comparisons paragraph to the manuscript, in blue below, noting that we treat individual edge-level political differences as exploratory rather than fully confirmatory, and rely on the joint cluster-permutation test (already reported) for the primary statistical inference.

***Technical note on multiple comparisons.*** Table 4 (appendix) reports permutation p-values for each pairwise edge comparison. Because 21 edges are tested simultaneously, we additionally report Benjamini-Hochberg false-discovery-rate-adjusted p-values and Holm-adjusted p-values alongside the raw values. Under cluster (state-level) permutation with unregularised precision matrices, edges may not survive either FDR or Holm correction at alpha = 0.05, which reflects the limited power available from only 50 state-level clusters relative to the number of edges tested, rather than an absence of political-stratum differences. We therefore treat the edge-level political differences reported here as exploratory rather than conclusively confirmatory, and rely on the joint cluster-permutation test (reported above) for the primary statistical inference about political-stratum differences.

Observation 8: On new construction: raw counts versus per-capita….

Answer: Yes, the Zillow New Construction measure is a raw count of construction sales, whereas HUD funding is per-capita. Raw counts are mechanically larger in populous, high-demand states, so the observed positive edge between New Construction and Rent is at least as consistent with construction responding to high demand as with construction raising rents. We have added an acknowledgment in Appendix E recording this fact of the New Construction sensitivity analysis. We elected not to re-estimate the sensitivity with per-capita normalization because (a) the New Construction analysis is a sensitivity rather than a main result, (b) the small sample (n = 86 state-years over 2018-2019) would not distinguish specifications with much statistical confidence, and (c) your main substantive point stands regardless of the specification: the direction of any potential causation between construction and rent cannot be identified from these data without instrumental variation or a richer supply-side model. We consider a properly normalized construction sensitivity using per-capita measures, permits/completions where available, and lagged specifications to be part of the follow-on work mentioned under Comment 3 above.
