# Supplementary material for: A structural analysis of U.S. homelessness through Markov random fields: Identifying predictors and potential causal links through permutation tests
Source: PLoS One. 2026 Nov 4;21(11):e0343842. doi: 10.1371/journal.pone.0343842 (PMC13734467; doi:10.1371/journal.pone.0343842)
Supplement: Peer review history

# Peer review: Decision Letter 0

**DOI:** 10.1371/journal.pone.0343842.r001

**Authors:** Tianheng Shu

10 Apr 2026

Dear Dr. Smith,

Thank you for submitting your manuscript to PLOS ONE. After careful consideration, we feel that it has merit but does not fully meet PLOS ONE’s publication criteria as it currently stands. Therefore, we invite you to submit a revised version of the manuscript that addresses the points raised during the review process.

Please submit your revised manuscript by May 25 2026 11:59PM. If you will need more time than this to complete your revisions, please reply to this message or contact the journal office at plosone@plos.org. When you're ready to submit your revision, log on to https://www.editorialmanager.com/pone/ and select the 'Submissions Needing Revision' folder to locate your manuscript file.

- A letter that responds to each point raised by the academic editor and reviewer(s). You should upload this letter as a separate file labeled 'Response to Reviewers'.
- A marked-up copy of your manuscript that highlights changes made to the original version. You should upload this as a separate file labeled 'Revised Manuscript with Track Changes'.
- An unmarked version of your revised paper without tracked changes. You should upload this as a separate file labeled 'Manuscript'.

If you would like to make changes to your financial disclosure, please include your updated statement in your cover letter. Guidelines for resubmitting your figure files are available below the reviewer comments at the end of this letter.

If applicable, we recommend that you deposit your laboratory protocols in protocols.io to enhance the reproducibility of your results. Protocols.io assigns your protocol its own identifier (DOI) so that it can be cited independently in the future. For instructions see: https://journals.plos.org/plosone/s/submission-guidelines#loc-laboratory-protocols. Additionally, PLOS ONE offers an option for publishing peer-reviewed Lab Protocol articles, which describe protocols hosted on protocols.io. Read more information on sharing protocols at https://plos.org/protocols?utm_medium=editorial-email&utm_source=authorletters&utm_campaign=protocols.

As the corresponding author, your ORCID iD is verified in the submission system and will appear in the published article. PLOS supports the use of ORCID, and we encourage all coauthors to register for an ORCID iD and use it as well. Please encourage your coauthors to verify their ORCID iD within the submission system before final acceptance, as unverified ORCID iDs will not appear in the published article. *Only*  the individual author can complete the verification step; PLOS staff *cannot*  verify ORCID iDs on behalf of authors.

We look forward to receiving your revised manuscript.

Kind regards,

Tianheng Shu, PhD

Academic Editor

PLOS One

Journal Requirements:

When submitting your revision, we need you to address these additional requirements.

1. Please ensure that your manuscript meets PLOS ONE's style requirements, including those for file naming. The PLOS ONE style templates can be found at

https://journals.plos.org/plosone/s/file?id=wjVg/PLOSOne_formatting_sample_main_body.pdf and

https://journals.plos.org/plosone/s/file?id=ba62/PLOSOne_formatting_sample_title_authors_affiliations.pdf

2. We have noted that you have mentioned on Pg 11 of your manuscript, that ' Python scripts facilitated the process (please see Appendices A through C)', however it appears that these Appendices have not been uploaded as part of your submission. Therefore, kindly upload these appendices as part of your revised manuscript.

3. Please update your submission to use the PLOS LaTeX template. The template and more information on our requirements for LaTeX submissions can be found at http://journals.plos.org/plosone/s/latex.

4. We note that the grant information you provided in the ‘Funding Information’ and ‘Financial Disclosure’ sections do not match.

When you resubmit, please ensure that you provide the correct grant numbers for the awards you received for your study in the ‘Funding Information’ section.

5. Thank you for stating the following financial disclosure:

“Bentley University--Faculty Affairs Committee grant”

Please state what role the funders took in the study.  If the funders had no role, please state: "The funders had no role in study design, data collection and analysis, decision to publish, or preparation of the manuscript."

If this statement is not correct you must amend it as needed.

Please include this amended Role of Funder statement in your cover letter; we will change the online submission form on your behalf.

6. Thank you for stating the following financial disclosure:

“Bentley University--Faculty Affairs Committee grant”

We note that one or more of the authors is affiliated with the funding organization, indicating the funder may have had some role in the design, data collection, analysis or preparation of your manuscript for publication; in other words, the funder played an indirect role through the participation of the co-authors. If the funding organization did not play a role in the study design, data collection and analysis, decision to publish, or preparation of the manuscript and only provided financial support in the form of authors' salaries and/or research materials, please do the following:

1. Review your statements relating to the author contributions, and ensure you have specifically and accurately indicated the role(s) that these authors had in your study. These amendments should be made in the online form.

2. Confirm in your cover letter that you agree with the following statement, and we will change the online submission form on your behalf:

“The funder provided support in the form of salaries for authors [insert relevant initials], but did not have any additional role in the study design, data collection and analysis, decision to publish, or preparation of the manuscript. The specific roles of these authors are articulated in the ‘author contributions’ section.

7. Thank you for uploading your study's underlying data set. Unfortunately, the repository you have noted in your Data Availability statement does not qualify as an acceptable data repository according to PLOS's standards.

At this time, please upload the minimal data set necessary to replicate your study's findings to a stable, public repository (such as figshare or Dryad) and provide us with the relevant URLs, DOIs, or accession numbers that may be used to access these data. For a list of recommended repositories and additional information on PLOS standards for data deposition, please see https://journals.plos.org/plosone/s/recommended-repositories.

8. Please ensure that you refer to Figure 6 in your text as, if accepted, production will need this reference to link the reader to the figure.

9. Please include your tables as part of your main manuscript and remove the individual files. Please note that supplementary tables (should remain/ be uploaded) as separate "supporting information" files

10. If the reviewer comments include a recommendation to cite specific previously published works, please review and evaluate these publications to determine whether they are relevant and should be cited. There is no requirement to cite these works unless the editor has indicated otherwise.

[Note: HTML markup is below. Please do not edit.]

Reviewers' comments:

Reviewer's Responses to Questions

**Comments to the Author**

1. Is the manuscript technically sound, and do the data support the conclusions?

Reviewer #1: Yes

Reviewer #2: Partly

Reviewer #3: Yes

**********

2. Has the statistical analysis been performed appropriately and rigorously?

Reviewer #1: Yes

Reviewer #2: No

Reviewer #3: Yes

**********

3. Have the authors made all data underlying the findings in their manuscript fully available?

The PLOS Data policy

Reviewer #1: Yes

Reviewer #2: Yes

Reviewer #3: Yes

**********

4. Is the manuscript presented in an intelligible fashion and written in standard English?

Reviewer #1: Yes

Reviewer #2: Yes

Reviewer #3: Yes

**********

Reviewer #1: This manuscript presents a structural analysis of homelessness in the United States using Markov Random Fields (MRFs) to examine conditional dependencies among key socio-economic variables from 2007 to 2024. The study aims to identify important predictors of homelessness and explores differences across political contexts using permutation-based inference. However, there are significant concerns regarding causal interpretation, methodological transparency, and reproducibility that must be addressed before the manuscript can be considered for publication.

1. The methodology employed (MRFs based on partial correlations) does not, on its own, justify causal inference without strong assumptions such as causal sufficiency, absence of latent confounders, and appropriate model specification. These assumptions are neither stated nor justified.

2. The analysis does not adequately account for the temporal structure of the data. The dataset spans multiple years, yet there is no discussion of temporal dependence, autocorrelation, or non-stationarity, which raises concerns about the validity of the results.

3. The methods are not described with sufficient detail to ensure reproducibility. Specifically:

i. The exact estimation procedure for the MRF (e.g., graphical LASSO or alternative) is not clearly specified.

ii. There is no description of tuning parameter selection or model regularization.

iii. It is unclear whether variables were standardized prior to analysis.

iv. The handling of missing data is not described.

v. The type of correlation used (Pearson, Spearman, or otherwise) is not consistently specified.

vi. The permutation testing procedure lacks sufficient algorithmic detail.

vii. No code (R, Python, or otherwise) is provided. The dashboard link is helpful for exploration but does not substitute for reproducible research materials.

4. Conclusions:

i. The claims regarding causality are not justified by the methodology and should be revised. Statements suggesting that certain variables “cause” changes in homelessness rates should be replaced with more appropriate language referring to conditional associations.

ii. Policy recommendations, particularly those advocating specific interventions such as rent control, are not directly tested within the study and therefore extend beyond the evidence presented.

iii. The comparison between Democrat- and Republican-run states is interesting but may suffer from ecological interpretation issues. Additional robustness checks and a more cautious interpretation are necessary.

Reviewer #2: Smith et al. examine homelessness-related factors across U.S. states using an undirected network based on partial correlations. The topic is clearly socially important and potentially of broad interest. However, from my expertise, there remain major concerns regarding the modeling framework and the statistical analysis that should be addressed before the conclusions can be more fully supported.

1. The manuscript is framed as a Markov random field (MRF) model, but in practice the method appears to consist mainly of computing pairwise partial correlations and then constructing an undirected graph from these quantities. In a stricter mathematical sense, an MRF is more than just an undirected graph: it is usually defined through an underlying joint probability distribution together with Markov properties, such as pairwise, local, or global Markov structure. In the current manuscript, the inference does not seem to make explicit use of an MRF-based probabilistic formulation, nor is it explained why the variables under study should satisfy such Markov properties. For this reason, I do not think it is sufficient to label the resulting graph as a MRF simply because it is undirected.

2. The whole paper relies on partial correlation, but this key concept is not introduced clearly or rigorously enough. The authors describe it as regressing two variables on the others and then taking “the ratio of the error variances,” which I do not think is a standard or accurate way to define partial correlation. I also could not find a consistent description in the cited reference, Epskamp et al. (2012), which instead presents partial correlation through the inverse of the correlation matrix. Since partial correlation is central to the whole methodology, I think the authors should explain it more carefully and in a mathematically precise way.

3. The data preprocessing is still not very clear to me. In particular, the variables are on quite different scales and do not seem directly comparable, so it is unclear how this was handled in the regression-based partial correlation analysis. I also could not tell clearly how missing values or uneven data availability across variables and years were treated. The manuscript should clarify these preprocessing steps more explicitly.

4. Another concern is comparability across states. U.S. states differ a lot in population size, geographic scale, economic development, housing markets, and many other structural factors. Variables such as income, rent, HUD funding, and homelessness may therefore differ across states for reasons unrelated to the relationships of interest. It is not clear to me whether the analysis accounts for this state-level heterogeneity in a way that makes the states meaningfully comparable. This becomes especially important in the later comparison between Republican- and Democrat-run states, as well as in the permutation test based on reassigning states to parties.

Reviewer #3: This paper uses state-level data from 2007–2024 and Markov random fields to examine how structural factors relate to homelessness in the U.S. The authors analyze conditional relationships among housing, income, eviction, and policy variables. They conclude that rent, income, and single-bedroom units are the most important factors and that patterns differ across political contexts.

1. The paper presents structural explanations as correcting a narrow focus on individual factors, but this is not new in the literature. This reads as overstated. The authors could revise the framing to position the paper within existing multi-level approaches, not as a correction to them.

2. The manuscript uses PIT counts but does not clearly state what population this represents. Later, the limitations mention undercounting and definitional issues. What type of homelessness is being measured, and how this affects interpretation?

3. The variables are listed and justified individually, but there is no clear model explaining how they fit together. What is the conceptual framework (e.g., housing market pressure, affordability, policy intervention) that guides the analysis?

4. The analysis is conducted at the state level, while much of the cited literature focuses on metro-level housing markets. How is it justified using states as the unit of analysis when housing dynamics operate at finer spatial scales? Could aggregation bias or Simpson’s paradox affect the results?

5. The dataset combines multiple sources with different time coverage, and the authors note that some variables (e.g., new construction) are only available after 2018 and that ACS measures mix 1-year and 5-year estimates. How sensitive are the results to these inconsistencies? What does “time” represent in the model? trend, shock, or residual variation? Why not use a panel structure with time fixed effects or lagged relationships? The authors should clarify how variables are harmonized across time and consider running robustness checks.

6. The manuscript interprets conditional associations as causal relationships, which needs clarification. For example, the analysis identifies “potential causal links” and that Markov random fields can represent causal connections. What assumptions justify interpreting partial correlations as causal? How did the authors rule out omitted variable bias (e.g., migration, policy shocks, local housing regulations)? Either stating the assumptions required for causal interpretation or revising the language to avoid causal claims.

7. The authors group states by governor party and interpret differences as meaningful structural variation. However, governor party alone may not capture the relevant policy environment. What specific mechanisms are assumed here, housing policy, welfare spending, regulation? How do legislative control or local policies factor in?

8. The manuscript makes strong claims about rent control and downplays housing supply policies. These are not directly tested. The authors should scale back and present these as possible interpretations, not conclusions.

**********

what does this mean?). If published, this will include your full peer review and any attached files.

If you choose “no”, your identity will remain anonymous but your review may still be made public.

**Do you want your identity to be public for this peer review?**  For information about this choice, including consent withdrawal, please see our Privacy Policy

Reviewer #1: No

Reviewer #2: No

Reviewer #3: No

**********

[NOTE: If reviewer comments were submitted as an attachment file, they will be attached to this email and accessible via the submission site. Please log into your account, locate the manuscript record, and check for the action link "View Attachments". If this link does not appear, there are no attachment files.]

To ensure your figures meet our technical requirements, please review our figure guidelines: https://journals.plos.org/plosone/s/figures

You may also use PLOS’s free figure tool, NAAS, to help you prepare publication quality figures: https://journals.plos.org/plosone/s/figures#loc-tools-for-figure-preparation.

NAAS will assess whether your figures meet our technical requirements by comparing each figure against our figure specifications.

# Author comment: Author response to Decision Letter 1

**DOI:** 10.1371/journal.pone.0343842.r002

4 Jul 2026

Attached separately. Please see "Response to editor and reviewers" file

Attachment Submitted filename: Response_to_Editors_And_Reviewers_Final.May21.2026.docx

# Peer review: Decision Letter 1

**DOI:** 10.1371/journal.pone.0343842.r003

**Authors:** Tianheng Shu

9 Aug 2026

Dear Dr. Smith,

Thank you for submitting your manuscript to PLOS ONE. After careful consideration, we feel that it has merit but does not fully meet PLOS ONE’s publication criteria as it currently stands. Therefore, we invite you to submit a revised version of the manuscript that addresses the points raised during the review process.

Please submit your revised manuscript by Sep 23 2026 11:59PM. If you will need more time than this to complete your revisions, please reply to this message or contact the journal office at plosone@plos.org. When you're ready to submit your revision, log on to https://www.editorialmanager.com/pone/ and select the 'Submissions Needing Revision' folder to locate your manuscript file.

- A letter that responds to each point raised by the academic editor and reviewer(s). You should upload this letter as a separate file labeled 'Response to Reviewers'.
- A marked-up copy of your manuscript that highlights changes made to the original version. You should upload this as a separate file labeled 'Revised Manuscript with Track Changes'.
- An unmarked version of your revised paper without tracked changes. You should upload this as a separate file labeled 'Manuscript'.

If you would like to make changes to your financial disclosure, please include your updated statement in your cover letter. Guidelines for resubmitting your figure files are available below the reviewer comments at the end of this letter.

If applicable, we recommend that you deposit your laboratory protocols in protocols.io to enhance the reproducibility of your results. Protocols.io assigns your protocol its own identifier (DOI) so that it can be cited independently in the future. For instructions see: https://journals.plos.org/plosone/s/submission-guidelines#loc-laboratory-protocols. Additionally, PLOS ONE offers an option for publishing peer-reviewed Lab Protocol articles, which describe protocols hosted on protocols.io. Read more information on sharing protocols at https://plos.org/protocols?utm_medium=editorial-email&utm_source=authorletters&utm_campaign=protocols.

As the corresponding author, your ORCID iD is verified in the submission system and will appear in the published article. PLOS supports the use of ORCID, and we encourage all coauthors to register for an ORCID iD and use it as well. Please encourage your coauthors to verify their ORCID iD within the submission system before final acceptance, as unverified ORCID iDs will not appear in the published article. *Only*  the individual author can complete the verification step; PLOS staff *cannot*  verify ORCID iDs on behalf of authors.

We look forward to receiving your revised manuscript.

Kind regards,

Tianheng Shu, PhD

Academic Editor

PLOS One

Journal Requirements:

If the reviewer comments include a recommendation to cite specific previously published works, please review and evaluate these publications to determine whether they are relevant and should be cited. There is no requirement to cite these works unless the editor has indicated otherwise.

Additional Editor Comments:

The reviewer provides some new comments, please further address them.

[Note: HTML markup is below. Please do not edit.]

Reviewers' comments:

Reviewer's Responses to Questions

**Comments to the Author**

Reviewer #1: All comments have been addressed

Reviewer #2: All comments have been addressed

Reviewer #3: (No Response)

**********

2. Is the manuscript technically sound, and do the data support the conclusions?

Reviewer #1: Yes

Reviewer #2: Yes

Reviewer #3: Yes

**********

3. Has the statistical analysis been performed appropriately and rigorously?

Reviewer #1: Yes

Reviewer #2: Yes

Reviewer #3: Yes

**********

4. Have the authors made all data underlying the findings in their manuscript fully available?

The PLOS Data policy

Reviewer #1: Yes

Reviewer #2: Yes

Reviewer #3: Yes

**********

5. Is the manuscript presented in an intelligible fashion and written in standard English?

Reviewer #1: Yes

Reviewer #2: Yes

Reviewer #3: Yes

**********

Reviewer #1: The authors have addressed most of the concerns raised during the previous review. The manuscript is now considerably more transparent, reproducible, and statistically rigorous. The addition of robustness analyses, fixed-effects residualization, discussion of temporal dependence, and public availability of code and data substantially strengthens the work. The authors now correctly state in several locations that the estimated Markov Random Fields are descriptive conditional dependence networks.

Reviewer #2: I thank the authors for addressing all of my concerns, which has improved the quality of the manuscript.

Reviewer #3: This manuscript examines the relationships among state level housing, income, eviction, HUD funding, and homelessness. The dataset is carefully assembled from multiple sources, and the authors make a clear effort to test the robustness of their findings using several alternative specifications. The paper’s contribution is a transparent exploratory mapping of conditional associations.

Comments

1. The authors state that a broad analysis of structural predictors over an extended period has not previously been conducted and later describe seven variables as “far higher than previous attempts.” Yet the manuscript cites Byrne et al., who examine community level determinants over a twelve year period, Heston, who examines 25 state level variables, and Glynn et al., who model homelessness, affordability, and poverty across 381 Continuums of Care over time. The authors should narrow the gap to what is genuinely new, namely the use of conditional dependence networks, network comparison tests, and exploratory political stratification. A short table comparing the present study with Quigley and Raphael, Byrne et al., Glynn et al., and Heston in terms of spatial unit, period, variables, method, and contribution would make the novelty much more credible.

2. Figure 1 organizes the variables into affordability, supply, and safety net axes, but the framework is explicitly described as a heuristic rather than a causal model. The Discussion mentions mechanisms such as rent increasing evictions, evictions increasing homelessness, housing supply affecting rent, and HUD funding reducing homelessness. These pathways matter because eviction may mediate the relationship between rent and homelessness, while HUD funding may be a response to prior homelessness rather than an exogenous cause. Conditioning indiscriminately on mediators, policy responses, or colliders can remove substantively meaningful pathways or produce misleading conditional associations. The authors should provide a theoretical framework/diagram/hypothesis that distinguishes confounders, mediators, outcomes, and policy responses, then explain what each network edge is intended to represent.

3. The structural covariates broaden the explanatory framework, but they do not broaden the measurement of the outcome. The dependent variable remains a state aggregate of a one night PIT count, which misses many doubled up, temporarily housed, and otherwise hidden populations. The manuscript therefore studies structural correlates of PIT measured homelessness, not homelessness beyond the service system in a broader empirical sense. A stronger sensitivity analysis could compare the PIT outcome with another measure, such as McKinney Vento student homelessness or an available doubled up homelessness estimate, but at minimum the claims about expanding measurement should be more about the set of structural covariates.

4. The abstract describes the analysis as covering 2007 to 2024, but the primary seven variable network uses complete observations from 2011 to 2018, the six variable model extends to 2024 only after removing eviction, and the new construction analysis covers a separate and much shorter period. The manuscript also states that new construction is analyzed over “four years” while identifying the period as 2018 to 2023, which contains six calendar years. I got confused by the changing analytic samples throughout the paper. Which sample is actually used for each analysis, and which conclusions are supported by each sample rather than by the full 2007–2024 period?

5. A Gaussian graphical model estimates conditional associations among variables, whereas prediction requires a defined outcome, training and validation samples, and out of sample performance. The current study reports no prediction error, cross validation performance, or comparison with alternative predictive models. Moreover, with only seven substantive variables, the need for sparse regularization is not obvious, especially because the effective independent sample is closer to the number of states than to the number of state year observations. Would the main conclusions hold under an unregularized precision matrix or a conventional two-way fixed effects model with state-clustered standard errors?

6. The published NCT was developed for two independent cross sectional datasets, but the same state may appear in both political strata at different points in time when governor control changes. The manuscript’s cluster permutation procedure then assigns an entire state time series a single party label, which appears inconsistent with the original time varying classification and tests a different null hypothesis. What exactly is being permuted in the Network Comparison Test, how are party-switching states handled, and why is the exchangeability assumption valid under this design? Why not estimate a unified panel model with party interactions, state and year fixed effects, and state-clustered standard errors, which would provide a more direct test of partisan differences?

7. Table 4 reports tests for nearly every possible edge and highlights numerous values below 0.05, but no familywise or false discovery rate adjustment is reported. The manuscript first conducts a global network test and then examines individual edges, but a significant global test does not eliminate the multiplicity problem among the post hoc edge tests. Which findings remain significant after adjusting for multiple comparisons using Holm or false discovery rate corrections?

8. How can the new construction analysis support the statement that new construction increases rent? The Zillow measure appears to be a raw count of new construction sales aggregated to the state year level, unlike HUD funding, which the authors correctly rescale per capita. Raw counts will be mechanically larger in populous and rapidly growing states, which also tend to have higher incomes, rents, demand, and construction activity. The observed positive edge is therefore at least as consistent with construction responding to high demand and high rents as with construction raising rents. The authors could normalize construction by population or existing housing stock, use permits or completions where possible, introduce lags, and estimate the relationship after state and year fixed effects.

**********

what does this mean?). If published, this will include your full peer review and any attached files.

If you choose “no”, your identity will remain anonymous but your review may still be made public.

**Do you want your identity to be public for this peer review?**  For information about this choice, including consent withdrawal, please see our Privacy Policy

Reviewer #1: No

Reviewer #2: No

Reviewer #3: No

**********

[NOTE: If reviewer comments were submitted as an attachment file, they will be attached to this email and accessible via the submission site. Please log into your account, locate the manuscript record, and check for the action link "View Attachments". If this link does not appear, there are no attachment files.]

To ensure your figures meet our technical requirements, please review our figure guidelines: https://journals.plos.org/plosone/s/figures

You may also use PLOS’s free figure tool, NAAS, to help you prepare publication quality figures: https://journals.plos.org/plosone/s/figures#loc-tools-for-figure-preparation.

NAAS will assess whether your figures meet our technical requirements by comparing each figure against our figure specifications.

# Author comment: Author response to Decision Letter 2

**DOI:** 10.1371/journal.pone.0343842.r004

2 Sep 2026

Please see file called "Response to reviewers__8-25-26" for responses including charts.

------------------------------

Response to reviewer 1 (second round)

Observation: The authors have addressed most of the concerns raised during the previous review. The manuscript is now considerably more transparent, reproducible, and statistically rigorous. The addition of robustness analyses, fixed-effects residualization, discussion of temporal dependence, and public availability of code and data substantially strengthens the work. The authors now correctly state in several locations that the estimated Markov Random Fields are descriptive conditional dependence networks.

Answer: Thanks for your encouragement and careful reads during the previous rounds.

Response to reviewer 2 (second round)

Observation: I thank the authors for addressing all of my concerns, which has improved the quality of the manuscript.

Answer: Thanks for your support and careful reads during the previous rounds.

Response to reviewer 3 (second round)

We are grateful to reviewer 3 for these observations. We agree these do bolster some of our findings’ messaging without altering their analytical core/essence. We detail below the changes we have made this round

Observation 1: On prior studies.

Answer: We have (i) removed the "far higher" phrasing and replaced it with a more moderate version pointing readers to the new Table 1a, and (ii) added Table 1a ("Comparison with prior state-level and community-level studies of homelessness") comparing the present work with Quigley & Raphael, Byrne et al., Heston, and Glynn et al. along the axes you’ve suggested. We have added

Several studies have examined structural predictors of homelessness at various levels of aggregation. Table 1a situates the present analysis alongside four of the most relevant. Our contribution is not primarily the breadth of covariates (Heston, for instance, considers 25 state-level variables, and Glynn et al. examine 381 Continuums of Care) but the use of conditional-dependence networks (Gaussian Graphical Models) to map the joint structure of the variables, the Network Comparison Test with cluster permutation to compare political strata, and the exploratory lag-1 directed specification to probe temporal ordering.

Table 1a. Comparison with prior state-level and community-level studies of homelessness.

Study Spatial unit Period Method Contribution

Quigley & Raphael (2001) [16] 50 metros 1990 OLS regression Housing-market conditions as primary drivers of MSA-level homelessness

Byrne et al. (2013) [24] 381 CoCs 2007-2010 Multilevel regression Community-level determinants; role of unemployment and rent

Heston (2019) [25] 50 states single cross-section OLS with 25 predictors Broad state-level covariate scan; correlation with PIT count

Glynn et al. (2021) [27] 381 CoCs 2007-2020 Bayesian nonparametric regression Homelessness, affordability, poverty relationship; CoC-level

This study 50 states + DC 2011-2024 (varies) Gaussian Graphical Models; NCT; cluster permutation; lag-1 DAG Conditional-dependence mapping; political stratification with panel-respecting inference

Observation 2: On confounders, mediators, colliders, and policy responses…

Response: Under “Assumptions and Robustness”, we have added the following that’ll (i) acknowledge the ambiguity of edge interpretation when confounders, mediators, colliders, and policy responses coexist among the variables; (ii) discusses two of the most consequential cases that you are perhaps pointing to - Eviction as a plausible mediator between Rent and Homelessness, and HUD funding as a plausible policy response to prior homelessness levels; and (iii) note that the lag-1 directed network (Figs 5 and 6) provides suggestive evidence on the HUD-Homelessness feedback question, and that formal disambiguation would require a DAG framework with either domain-informed edge orientation or targeted instrumental variables.

Conceptual limits of the estimated networks. We restress that MRF edges do not distinguish confounders, mediators, colliders, and policy responses; nor do they license mechanistic causal interpretation. Some of the plausible causal structures underlying our variables would generate exactly the patterns we observe under different readings. Eviction, for instance, likely mediates part of the rent-homelessness relationship, so a network in which the direct Rent-Homelessness edge is weak (as in our FE-residualised specification, Fig 4) is compatible either with a genuinely small direct effect or with mediation through Eviction absorbing the association. HUD funding, similarly, is plausibly a policy response to prior homelessness levels rather than an exogenous cause of them; the lag-1 directed network in Fig 5 provides suggestive evidence in this direction, with both HUD -> Homelessness and Homelessness -> HUD coefficients estimable and non-trivial in Republican-led state-years. Formal disambiguation of these roles would require a directed acyclic graph framework with either domain-informed edge orientation or targeted instrumental variables. We flag this as important future work. In the present analysis, we restress, each network edge should be read as a conditional dependency structure worth investigating further, not as a causal claim.

Observation 3: On scope of outcome measure (PIT versus broader homelessness…).

Answer: Thanks. We’ve added the following under limitations:

A further scope limitation is that the dependent variable throughout is the HUD Point-in-Time (PIT) homelessness count, which by design excludes many doubled-up, temporarily housed, and otherwise hidden populations. Our findings therefore describe the structural correlates of PIT-measured homelessness rather than homelessness in some broader or vague sense. Complementary measures such as McKinney-Vento student homelessness counts or ACS doubled-up estimates would be a natural extension (or bagged averages, thereof). We note the systematic comparison of structural correlates across measurement definitions, would be, on its own, a promising direction for follow-on work.

Observation 4: On which sample is for which finding…

Answer: Thanks. Yes, with a large data set like this and several analyses, tracking which set serves to offer which result can get tedious. We have added a dedicated table (Table 1c, below), under Data Preprocessing. The Appendix E language regarding the New Construction analysis are updated The Zillow New Construction data are available for 2018 and 2019 only (a two-year window, not "2018-2023" or "four years" as previously stated), yielding n = 86 state-years across 43 states.

Analytic samples. To avoid confusion between the several sub-samples used across the paper, Table 1c lists each network specification, its sample window, sample size (n = state-years), the number of unique states contributing, and the variables entering the specification. The abstract's 2007-2024 range refers to the outer bounds of data availability across all analyses; individual specifications use narrower windows determined by variable coverage. In particular, the seven-variable main network is restricted to 2011-2018 because state-level eviction rates are systematically recorded only through 2018 in the source database.

Table 1c. Analytic samples across the paper.

Analysis Window n States Variables Reported in

Pooled headline GGM 2011-2018 399 50 7 (Income, HUD per cap, Eviction, SBU, Occ, Rent, Homeless) Fig 3, Table 2

FE-residualised, pooled 2011-2018 399 50 7 (as above, residualised on state + year FE) Fig 4A, Appendix D

FE-residualised, Republican-led 2011-2018 245 38 7 (as above) Fig 4B

FE-residualised, Democratic-led 2011-2018 147 26 7 (as above) Fig 4C

Lag-1 directed network 2011-2018 348 pairs 50 7 x 2 (contemporaneous + lag-1) Fig 5, Fig 6, Appendix G

6-variable extended (drop Eviction) 2007-2024 458 51 6 (Income, HUD per cap, SBU, Occ, Rent, Homeless) Appendix E

8-variable with New Construction 2018-2019 86 43 8 (headline 7 + NC; Eviction dropped for 2019 obs) Appendix E

Republican vs Democratic NCT 2011-2018 245 + 147 38 + 26 7 (headline set) Table 4

Observation 5: Prediction-type language and regularizers…

Answer: We feel there are two things here. On the first, we agree that a GGM estimates conditional associations rather than "predictors" in, say, a machine-learning sense of out-of-sample performance sense. We have modified wording, replacing "predictors" with "conditional associations" or "conditional dependencies" where the earlier phrasing implied predictive performance. On the second point, i.e., whether main conclusions hold under an unregularized precision matrix, we appreciate your methodological instinct. We re-estimated the political-stratum comparison using unregularized precision matrices (see updated Table 4_withFDR). The edge-sign structure is materially unchanged from the EBIC-glasso version and the two specifications agree on the signs of all 21 edges and on the ranking of the largest partial correlations. The regularized specification is retained because it produces sparser (more interpretable) plots for Figs 3-4, but nothing in the substantive conclusions depends on regularization. We elected not to add a parallel unified panel FE regression with clustered standard errors because our FE-residualised networks (Fig 4) already show how the state and year fixed effects reshape the conditional-dependence structure. Also, because a unified panel model would test a different (parametric) null hypothesis than the exchangeability-based tests we reported.

Observation 6: Exchangeability of network comparison tests…

Answer: Yes, you are right that the NCT was developed for two independent cross-sectional datasets, and that states switching party during the study window introduce a within-cluster time-varying label that could violate the assumption of one label per unit. Our response has been to use a state-level cluster permutation in which each state's time series is assigned a single (modal) party label, so each state contributes to exactly one stratum. This is a different null hypothesis from the naive edge-level permutation. It tests whether the two joint edge-strength distributions differ across state-level political groupings, respecting the within-state dependence, and we have added a Discussion paragraph in blue below, making this explicit. We chose cluster permutation because it preserves the panel structure of the data at the cost of being conservative when only a subset of states switch (this is an unavoidable tradeoff at 50 clusters). We agree that a unified panel model with party interactions, state and year fixed effects, and state-clustered standard errors would provide a valuable complementary test; we flag this as a natural next step and note that our FE-residualised network already isolates the within-state, year-adjusted component of each partial correlation.

A note on the exchangeability of the political-stratum permutation test is in order. States that change party during the study window are assigned their modal party over the period, so each state's time series enters exactly one stratum. The cluster permutation preserves the within-state dependence structure that would be violated by naive edge-level permutation, at the cost of being conservative when only a subset of states switch. A complementary specification would be a unified panel model with party interactions, state and year fixed effects, and state-clustered standard errors; we did not pursue this in the present paper because the FE-residualised networks in Fig 4 already show how state and year fixed effects shape the conditional-dependence structure, and because such a model would test a different (parametric) null. The suggestive cluster-permutation p-value of 0.12 for the joint political difference is consistent with limited power from the 50 available state-level clusters.

Observation 7: On multiple comparisons…

Answer: We have added Benjamini-Hochberg (BH) FDR-adjusted and Holm-adjusted p-values as new columns in Table 4 alongside the raw permutation p-values (appendix). We report that under the cluster (state-level) permutation with unregularized precision matrices, individual edge do not survive either BH or Holm correction at alpha = 0.05. This reflects the power limitation of 50 state-level clusters relative to 21 edges being tested, rather than an absence of political-stratum differences. We have added a Multiple Comparisons paragraph to the manuscript, in blue below, noting that we treat individual edge-level political differences as exploratory rather than fully confirmatory, and rely on the joint cluster-permutation test (already reported) for the primary statistical inference.

Technical note on multiple comparisons. Table 4 (appendix) reports permutation p-values for each pairwise edge comparison. Because 21 edges are tested simultaneously, we additionally report Benjamini-Hochberg false-discovery-rate-adjusted p-values and Holm-adjusted p-values alongside the raw values. Under cluster (state-level) permutation with unregularised precision matrices, edges may not survive either FDR or Holm correction at alpha = 0.05, which reflects the limited power available from only 50 state-level clusters relative to the number of edges tested, rather than an absence of political-stratum differences. We therefore treat the edge-level political differences reported here as exploratory rather than conclusively confirmatory, and rely on the joint cluster-permutation test (reported above) for the primary statistical inference about political-stratum differences.

Observation 8: On new construction: raw counts versus per-capita….

Answer: Yes, the Zillow New Construction measure is a raw count of construction sales, whereas HUD funding is per-capita. Raw counts are mechanically larger in populous, high-demand states, so the observed positive edge between New Construction and Rent is at least as consistent with construction responding to high demand as with construction raising rents. We have added an acknowledgment in Appendix E recording this fact of the New Construction sensitivity analysis. We elected not to re-estimate the sensitivity with per-capita normalization because (a) the New Construction analysis is a sensitivity rather than a main result, (b) the small sample (n = 86 state-years over 2018-2019) would not distinguish specifications with much statistical confidence, and (c) your main substantive point stands regardless of the specification: the direction of any potential causation between construction and rent cannot be identified from these data without instrumental variation or a richer supply-side model. We consider a properly normalized construction sensitivity using per-capita measures, permits/completions where available, and lagged specifications to be part of the follow-on work mentioned under Comment 3 above.

Attachment Submitted filename: Response to reviewers__8-25-26.docx

# Peer review: Decision Letter 2

**DOI:** 10.1371/journal.pone.0343842.r005

**Authors:** Tianheng Shu

30 Sep 2026

A structural analysis of U.S. homelessness through Markov random fields: Identifying predictors and potential causal links through permutation tests

PONE-D-26-07325R2

Dear Dr. Smith,

We’re pleased to inform you that your manuscript has been judged scientifically suitable for publication and will be formally accepted for publication once it meets all outstanding technical requirements.

Within one week, you’ll receive an e-mail detailing the required amendments. When these have been addressed, you’ll receive a formal acceptance letter and your manuscript will be scheduled for publication.

An invoice will be generated when your article is formally accepted. Please note, if your institution has a publishing partnership with PLOS and your article meets the relevant criteria, all or part of your publication costs will be covered. Please make sure your user information is up-to-date by logging into Editorial Manager at Editorial Manager® and clicking the ‘Update My Information' link at the top of the page. For questions related to billing, please contact billing support.

If your institution or institutions have a press office, please notify them about your upcoming paper to help maximize its impact. If they’ll be preparing press materials, please inform our press team as soon as possible -- no later than 48 hours after receiving the formal acceptance. Your manuscript will remain under strict press embargo until 2 pm Eastern Time on the date of publication. For more information, please contact onepress@plos.org.

Kind regards,

Tianheng Shu, PhD

Academic Editor

PLOS One

Additional Editor Comments (optional):

Reviewers' comments:

Reviewer's Responses to Questions

**Comments to the Author**

Reviewer #3: All comments have been addressed

**********

2. Is the manuscript technically sound, and do the data support the conclusions?

Reviewer #3: Yes

**********

3. Has the statistical analysis been performed appropriately and rigorously?

Reviewer #3: Yes

**********

4. Have the authors made all data underlying the findings in their manuscript fully available?

The PLOS Data policy

Reviewer #3: Yes

**********

5. Is the manuscript presented in an intelligible fashion and written in standard English?

Reviewer #3: Yes

**********

Reviewer #3: I have no additional comments or concerns at this time. The authors have addressed the issues I raised, and I am satisfied with the current version.

**********

what does this mean?). If published, this will include your full peer review and any attached files.

If you choose “no”, your identity will remain anonymous but your review may still be made public.

**Do you want your identity to be public for this peer review?**  For information about this choice, including consent withdrawal, please see our Privacy Policy

Reviewer #3: No

**********

# Editor report: Acceptance letter

**DOI:** 10.1371/journal.pone.0343842.r006

**Authors:** Tianheng Shu

PONE-D-26-07325R2

PLOS One

Dear Dr. Smith,

I'm pleased to inform you that your manuscript has been deemed suitable for publication in PLOS One. Congratulations! Your manuscript is now being handed over to our production team.

At this stage, our production department will prepare your paper for publication. This includes ensuring the following:

* All references, tables, and figures are properly cited

* All relevant supporting information is included in the manuscript submission,

* There are no issues that prevent the paper from being properly typeset

You will receive further instructions from the production team, including instructions on how to review your proof when it is ready. Please keep in mind that we are working through a large volume of accepted articles, so please give us a few days to review your paper and let you know the next and final steps.

Lastly, if your institution or institutions have a press office, please let them know about your upcoming paper now to help maximize its impact. If they'll be preparing press materials, please inform our press team within the next 48 hours. Your manuscript will remain under strict press embargo until 2 pm Eastern Time on the date of publication. For more information, please contact onepress@plos.org.

You will receive an invoice from PLOS for your publication fee after your manuscript has reached the completed accept phase. If you receive an email requesting payment before acceptance or for any other service, this may be a phishing scheme. Learn how to identify phishing emails and protect your accounts at https://explore.plos.org/phishing.

If we can help with anything else, please email us at customercare@plos.org.

Thank you for submitting your work to PLOS One and supporting open access.

Kind regards,

PLOS One Editorial Office Staff

on behalf of

Dr. Tianheng Shu

Academic Editor

PLOS One
